# Reliability of EEG Measures of Interaction: A Paradigm Shift Is Needed to Fight the Reproducibility Crisis

**DOI:** 10.3389/fnhum.2017.00441

**Published:** 2017-08-30

**Authors:** Yvonne Höller, Andreas Uhl, Arne Bathke, Aljoscha Thomschewski, Kevin Butz, Raffaele Nardone, Jürgen Fell, Eugen Trinka

**Affiliations:** ^1^Department of Neurology, Christian Doppler Medical Centre and Centre for Cognitive Neuroscience, Paracelsus Medical University Salzburg, Austria; ^2^Department of Computer Sciences, Paris Lodron University Salzburg, Austria; ^3^Department of Mathematics, Paris Lodron University Salzburg, Austria; ^4^Spinal Cord Injury and Tissue Regeneration Center, Paracelsus Medical University Salzburg, Austria; ^5^Department of Neurology, Franz Tappeiner Hospital Merano, Italy; ^6^Department of Epileptology, University of Bonn Bonn, Germany

**Keywords:** reliability, reproducibility, connectivity, brain networks, MVAR model, TLE, MCI

## Abstract

Measures of interaction (*connectivity*) of the EEG are at the forefront of current neuroscientific research. Unfortunately, test-retest reliability can be very low, depending on the measure and its estimation, the EEG-frequency of interest, the length of the signal, and the population under investigation. In addition, artifacts can hamper the continuity of the EEG signal, and in some clinical situations it is impractical to exclude artifacts. We aimed to examine factors that moderate test-retest reliability of measures of interaction. The study involved 40 patients with a range of neurological diseases and memory impairments (age median: 60; range 21–76; 40% female; 22 mild cognitive impairment, 5 subjective cognitive complaints, 13 temporal lobe epilepsy), and 20 healthy controls (age median: 61.5; range 23–74; 70% female). We calculated 14 measures of interaction based on the multivariate autoregressive model from two EEG-recordings separated by 2 weeks. We characterized test-retest reliability by correlating the measures between the two EEG-recordings for variations of data length, data discontinuity, artifact exclusion, model order, and frequency over all combinations of channels and all frequencies, individually for each subject, yielding a correlation coefficient for each participant. Excluding artifacts had strong effects on reliability of some measures, such as classical, real valued coherence (~0.1 before, ~0.9 after artifact exclusion). Full frequency directed transfer function was highly reliable and robust against artifacts. Variation of data length decreased reliability in relation to poor adjustment of model order and signal length. Variation of discontinuity had no effect, but reliabilities were different between model orders, frequency ranges, and patient groups depending on the measure. Pathology did not interact with variation of signal length or discontinuity. Our results emphasize the importance of documenting reliability, which may vary considerably between measures of interaction. We recommend careful selection of measures of interaction in accordance with the properties of the data. When only short data segments are available and when the signal length varies strongly across subjects after exclusion of artifacts, reliability becomes an issue. Finally, measures which show high reliability irrespective of the presence of artifacts could be extremely useful in clinical situations when exclusion of artifacts is impractical.

## 1. Introduction

Measures of interaction are usually known as *connectivity*, despite the criticism that the latter term is rather speculative (see also Horwitz, 2003; Rockland, 2015). While the term connectivity suggests that the measures characterize a physical connection, in most cases a statistical measure of interdependence is applied to signals recorded from the brain, which is not directly related to physical connections. A paradigm shift in the sense of re-thinking this concept of connectivity is highly warranted. Moreover, in order to estimate and interpret these statistical properties, some common problems need to be considered.

It was suggested that statistical weaknesses are the source of the so-called reproducibility crisis (Baker, 2016). In view of the importance of reliability of biomarkers for clinical trials and any measurement in general (Lachin, 2004), a detrimental problem is poor reproducibility of brain-network metrics (Welton et al., 2015). We propose that a further paradigm shift in neuroscience is needed in order to address the reproducibility crisis which has reached the research on brain networks.

Reproducibility in terms of test-retest reliability of resting state networks from magnetic resonance imaging was shown to be affected by the choice of the frequency band and the length of the time-series (Andellini et al., 2015). Graph metrics of non-directed functional networks derived from magnetoencephalography yielded an average intraclass-correlation coefficient of 0.60–0.65, with higher test-retest reliability in lower frequency networks compared to beta- and gamma frequency ranges (Deuker et al., 2009; Jin et al., 2011).

In addition to frequency, trial number and signal-to-noise ratio affect test-retest reliability of electroencephalographic (EEG) interactions (Miskovic and Keil, 2015). Most importantly, type of measure and the type of network characteristics exhibit varying test-retest reliability; phase-dependent measures show lower reliability than absolute power and classical coherence over 30 days (Cannon et al., 2012). Long term follow-ups of up to 2 years revealed intra-class correlation coefficients of 0.68–0.80 for global interactions and of 0.12–0.73 for graph measures (Hardmeier et al., 2014). Test-retest reliability in the sense of correlation between measures obtained from EEG recordings separated by 2 weeks for several directed measures of interaction varies largely between measures; some measures demonstrate high reliability with an average rho above 0.9 while others fall below 0.5 (Höller et al., 2017).

Measures can be categorized into directed and undirected measures, as being frequency dependent or frequency independent, linear or nonlinear, with the latter relying on information theory (Greenblatt et al., 2012). A common approach which is widely used for estimation of both directed and undirected, as well as frequency dependent and frequency independent measures of linear interaction is the multivariate autoregressive model (MVAR) (Marple, 1987; Greenblatt et al., 2012):

(1) $$\begin{array}{r} Y\left(n\right)=\overset{p}{\underset{k\;=1}{\sum }}A\left(k\right)Y\left(n-k\right)+U\left(n\right) \end{array}$$

where $Y\left(n\right)={\left[y_{1}\left(n\right),\ldots ,y_{M}\left(n\right)\right]}^{T}$ is a vector holding the values of the *M* channels at time *n*, *p* is the model order, *A*(*k*) are *M* × *M* coefficient matrices in which the element *a*_*ij*_(*k*) describes the dependence of *y*_*i*_(*n*) on *y*_*j*_(*n* − *k*) and *U*(*n*) is the innovation process, which is assumed to be composed of white and uncorrelated noise. Hence, the model with *p* coefficient matrices *A*(*k*) is estimated on a given number *N* of subsequent time samples. Naturally, the number of data samples should be larger than the number of estimates (Kus et al., 2004). For the multivariate autoregressive model we have to estimate *M*^2^ · *p* coefficients and we have *M* · *N* data samples.

It was suggested that *N*/(*M* · *p*) should be larger than 10 (Schlögl and Supp, 2006). However, whether this is a hard rule or to which extent it can be violated is unclear. The effect of the number of samples *N* with different estimation algorithms for estimation of the model coefficients was tested by Schloegl et al. (Schlögl, 2006) with a model order of 6 and *M* = 5. Thus, *N* was required to be at least 30 and ideally above 300. The authors reported varying numbers of *N* from 50 to 400. The results showed that indeed, with increasing N the mean square prediction error of the model decreased. However, even with *N* = 100 the mean square prediction error was <2, which was quite good in comparison to lower *N*. The study by Schloegl et al. (Schlögl, 2006) was based on artificially generated samples, in order to validate the predicted against the estimated values. The simulation may not reflect non-stationarity like artifacts or effects of drowsiness that occur when the EEG—like in the clinical setting—is being recorded over 20 min. After all, even if the estimation of the model is valid, the result can still suffer from low reliability, because of the non-stationary nature of the EEG (Hatz et al., 2015a).

Therefore, in addition to signal length, disruptions of the time-series can severely affect the result. Such disruptions may be introduced by artifacts, which are common in EEG studies, but also by pathological activity in patient populations like spikes in patients with epilepsy, unless these pathological events are the events of interest that should be modeled. Artifacts such as muscle activity or eye movements are typically excluded from the analysis. It was shown that the automated artifact analysis showed higher reliability over time than visual artifact analysis, which is likely attributed to the subjective nature of visual assessment (Hatz et al., 2015b).

However, excluding artifacts is not always a good option. First, in clinical applications, there may be instances of time which are unique and which need to be assessed regardless of the presence of artifacts. This is applicable to the onset of a seizure in patients with epilepsy, or specific moments of enhanced vigilance in chronically ill patients in a unresponsive wakefulness syndrome. Moreover, we hypothesize that excluding artifacts may additionally affect the model estimation. Leaving out the problematic segments of data causes discontinuity of the time series. Discontinuity of the time series can be a problem when applying the MVAR model. The MVAR model looks *k* = 1…*p* steps into the past. If there is an artifact in this period, this causes a gap in the EEG. When the model is applied to the concatenated EEG with excluded artifactual epochs, these gaps can cause troubles. We hypothesize further, that the size of these gaps could play a role.

In the present study, we aimed to characterize factors that affect test-retest reliability of measures of interaction derived from the EEG. We hypothesized that several factors contribute to the reliability:

1. length of the signal,
2. discontinuity of the signal,
3. frequency resolution,
4. and model order.

We systematically assessed the test-retest reliability of a set of interaction measures based on the multivariate autoregressive model over two EEG recordings in order to assess effects of the hypothesized moderators. The chosen datasets were resting-EEG recordings separated by 2 weeks from patients with mild cognitive impairment (MCI), subjective cognitive complaints (SCC), temporal lobe epilepsy (TLE), and healthy controls (HC). Thereby, in the present paper we extend our previous findings on variability between measures and between neurological subgroups (Höller et al., 2017) by examining the listed potential moderators of reliability. A better understanding of these factors could pave the way for a methodological paradigm shift in brain network research.

## 2. Methods

### 2.1. Ethics

The study was approved by the local Ethics Committee (Ethics Commission Salzburg/Ethikkommission Land Salzburg; number 415-E/1429) and was designed according to the Declaration of Helsinki. Written informed consent was obtained from all participants. Healthy participants were remunerated for their expenditure of time.

### 2.2. Subjects

We recruited a total sample of 70 participants at the Department of Neurology, Paracelsus Medical University Salzburg, Austria, from May 2012 to December 2015 within a larger study focused on memory disorders. After exclusion of participants who did not undergo both EEG-examinations (two TLEr, one TLEl, three HC) and whose EEG was of poor quality (one SCC, one TLEl, two HC) 60 participants remained for this analysis. Poor quality of the EEG was defined as less than 4 s in at least one of the two recordings after excluding segments of 500 ms according to the automatic data inspection (see Section 2.4).

The same data was used in a recent study on difference in test-retest reliability between patient populations (Höller et al., 2017). The data stems from a study in which we were interested in memory impairments, so that we report standard mini-mental state examinations in patients recruited from the memory clinic (MCI and SCC subgroups) and the Montreal cognitive assessment results for HCs, which was employed in order to have a sensitive measure that should disclose clinically relevant memory problems in the healthy subgroup. In this original study, patients with TLE were recruited regardless of memory complaints, so that no memory-screening was implemented. More detailed information can be retrieved from Table S1. The patients with MCI scored quite high on the MMSE, which might be attributed to the poor sensitivity of the MMSE in contrast to a full neuropsychological test battery. Indeed, the diagnosis was not exclusively based on the MMSE but on an extensive neuropsychological and neurological examination.

Patients with amnestic MCI or SCC were recruited in the memory outpatient clinic of the Department of Neurology, Paracelsus Medical University Salzburg, Austria. We defined patients with amnestic MCI according to level three and patients with SCC according to level two of the global deterioration scale for ageing and dementia described by Reisberg et al. (1982) and Gauthier et al. (2006). Diagnosis was based on multimodal neurological assessment, including imaging (high resolution 3T magnetic resonance tomography, and single photon emission computed tomography), and neuropsychological testing. We excluded patients whose memory complaints/impairment could be explained better by inflammatory, vascular, metabolic, or traumatic background, or by major depression, psychosis, or any pharmacological therapy.

Patients with refractory unilateral TLE were recruited in the epilepsy outpatient clinic of the Department of Neurology, Paracelsus Medical University Salzburg, Austria. Diagnosis was based on multimodal neurological assessment, including imaging (high resolution 3T magnetic resonance tomography, and single photon emission computed tomography), neuropsychological testing, and video-EEG examination for up to 5 days. We excluded patients with progressive lesions or immunological causes of epilepsy. Table S2 provides detailed information about the patients with TLE, including information of whether seizures occurred within 24 h before or after the EEG-recording took place (column “seizure”) and assessment of the EEG by a board certified neurophysiologist (column “findings”).

The sample of healthy participants was recruited amongst the students of the Paris Lodron University of Salzburg, Austria, as well as amongst senior citizens associations in order to match for sex and age. Healthy participants were free of a history for neurological or psychiatric diseases and were not receiving any psychoactive medication.

Table S3 lists the medication of all included participants. Medication was recorded on the first examination day. Since the two examination days were set independently of visits to the doctor, it is assumed that medication remains stable over the 2 weeks separating the two EEG recordings.

### 2.3. Data registration

EEG was recorded in a quiet room. Participants were instructed to close their eyes and stay awake. Recordings lasted for 2–3 min. We used a BrainCap with a 10–20 system and a BrainAmp (Brain Products GmbH, Germany) 16-bit ADC amplifier. The sampling rate was 500 Hz. Of the 32 recorded channels, one was used to monitor the lower vertical electrooculogram and one was used to measure electrocardiographic activity. Two were positioned at the earlobes for re-referencing purposes to remove the bias of the original reference, which was placed at FCz. Data analysis was conducted for data collected from the remaining 27 electrodes F3, F4, C3, C4, P3, P4, O1, O2, F7, F8, T7, T8, P7, P8, Fz, Cz, Pz, FC1, FC2, CP1, CP2, FC5, FC6, CP5, CP6, TP9, and TP10. Impedances were kept below 10 kΩ.

The two EEG sessions were arranged to take place at the same time of the day. For most participants, this requirement was met by performing EEG within the same time-range around noon, after lunch (1 pm.). This means that we aimed to keep the time difference between the two recordings below 3 h. For three participants (HC, SCC, TLEl) the time difference was approximately 4 h, for two patients (MCI, TLEr) the time difference was 6 h, and for one HC the time difference was 11 h.

Table S4 lists the results of a clinical evaluation of the EEGs of all participants included in this study.

### 2.4. Data preparation

Data was pre-processed with Brain Vision Analyzer (Version 1.05.0005, Brain Products GmbH). In order to re-reference all channels, a new reference was built by averaging the signal of both earlobe electrodes. Butterworth Zero Phase Filters were used for a high-pass filter from 1 Hz (time constant 0.1592 s, 48 dB/oct) and an additional notch filter (50 Hz) was applied.

An automatic artifact detection was carried out. Please note that the automation of this procedure ensures objectivity, which means at the same time that it is reproducible. Nevertheless, the nature and number of artifacts surely depends on the specific recording and participant. Maximal allowed voltage step per sampling point was 50 μV (values which exceeded this threshold were marked within a range of ±100 ms); maximal allowed absolute difference on an interval of 200 ms was 200 μV and lowest allowed absolute difference during an interval of 100 ms was 0.5 μV (values which exceeded this were marked with a surrounding of ±500 ms). The result of this artifact detection was reviewed visually in order to determine whether the automated detection yielded reasonable results and whether poor data quality was due to noise on the reference electrodes, which led to exclusion of the dataset.

The preprocessed data was exported into a generic data format and imported to Matlab® (release R2016b, The Mathworks, Massachusetts, USA).

### 2.5. Data length

In order to provide the same amount of data for each participant, data was shortened to the shortest available length across participants. The shortest length was 123.5 s. Thus, a total of 123.5·500· 27 = 1, 667, 250 samples were available for estimation of up to 27^2^ · 250 = 182, 250 coefficients for the model order 250. By doing so, *N*/(*M* · *p*) = 9.15 was in the upper part of the range of 1.67−13.33 as presented in Schlögl (2006), where a ratio >3 yielded a mean square prediction error <2 and robust results across different model estimators.

### 2.6. Data discontinuity and data length variation

**Figure 1:**
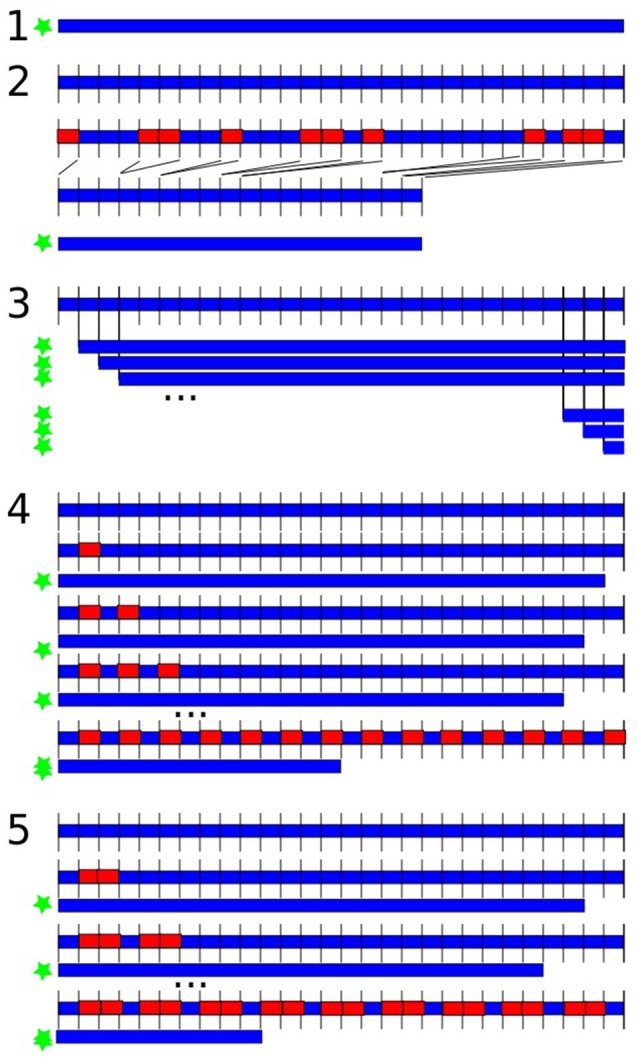
Variations of data length and discontinuity. The blue shape is the data, the red rectangles represent left-out data, the green star indicates which versions were submitted to feature extraction. (1) Analysis over the whole dataset; (2) excluding artifacts in 500 ms segments; (3) repeated feature estimation by shortening 500 ms at the beginning in each repetition; (4) data was segmented into 500 ms segments; repeated feature estimation by increasingly leaving out every second segment; (5) data was segmented into 500 ms segments; repeated feature estimation by increasingly leaving out every second and third segment.

Feature extraction was performed in 5 variants, illustrated in Figure 1:

1. **Complete data:** over the whole dataset (all 123.5 s); thus, the data was continuous but included artifacts.
2. **Artifact-free data:** data was segmented into 500 ms segments (250 sampling points); if the segment overlapped with a marked artifact, it was excluded from further analysis for all channels; the remaining segments were concatenated; thus, the data was discontinuous without artifacts.
3. **Length variation:** the estimation of the features was repeated by increasingly cutting 500 ms at the beginning of the data at each repetition; thus, the data was continuous with artifacts and we varied data length.
4. **0.5 s cut-outs:** data was segmented into 500 ms segments (250 sampling points); in loops the estimation of the features was repeated by increasingly leaving out every second segment; thus data was discontinuous with artifacts, we varied length and discontinuity.
5. **1 s cut-outs:** data was segmented into 500 ms segments (250 sampling points); in loops the estimation of the features was repeated by increasingly leaving out two segments after every third segment; thus data was discontinuous with artifacts, we varied length and discontinuity with larger gaps compared to the previous variant.

### 2.7. Frequency effects

We were interested in reliability of classical frequency ranges delta (1–4 Hz), theta (5–7 Hz), alpha (8–13 Hz), beta (14–30 Hz), gamma (31–80 Hz), and high gamma (81–125 Hz) and the effect of averaging within these frequency ranges on reliability.

When statistically determining and evaluating the reliability of the measures of interaction, we calculated reliability in three variants:

- **Frequency averaging:** reliabilities were calculated for all variants of discontinuity (1–5) when values were averaged in classical frequency ranges.
- **Frequency averaging/no averaging vs. model order and artifacts:** reliabilities were calculated for each model order from 1 to 250 and the complete data vs. artifact-free data scenarios, once when values were averaged and once when values were not averaged in classical frequency ranges before calculation of reliability.
- **Reliability within frequency ranges:** we calculated reliability for every frequency range separately, without averaging across frequencies, on the artifact-free data.

### 2.8. Feature extraction

We estimated a set of measures of interaction between all of the 27 selected channels. The estimation was performed for each of the participants. The measures were calculated based on the multivariate autoregressive model with the functions mvfreqz.m and mvar.m from the BioSig toolbox (Schlögl and Brunner, 2008) with model order from 1 to a maximum of 250, that is, equaling the length of the segmented data and enabling us to model at least one oscillation for each of the examined frequencies. In order to estimate the multivariate autoregressive model we used partial correlation estimation with unbiased covariance estimates (Marple, 1987), which was found to be the most accurate estimation method according to Schlögl (2006). The model was then transformed from the time-domain into the *z*-domain and the *f*-domain, which accordingly yield two transfer functions. The multivariate parameters in the frequency domain that can be derived from these transfer functions were computed for 1 Hz frequency steps between 1 and 125 Hz. The measures of interest were the following:

- **Spectrum:** This contains the auto- and the cross-spectrum, which is the Fourier transform of the cross-covariance function (Murthy, 1963).
- **Direct causality:** Direct causality was developed by Kaminski et al. (2001) to overcome the problem that the directed transfer function does not distinguish between direct and indirect information flows. Direct causality is the only measure that is not computed for each frequency.
- **Transfer function:** This transfer function is related to the non-normalized directed transfer function (Eichler, 2006).
- **Transfer function polynomial:** This is the frequency transform of a polynomial describing the transfer function. The absolute of the squared transfer function polynomial is the non-normalized partial directed coherence (Eichler, 2006).
- **Real valued coherence:** By considering the real part of the complex-valued coherence (Nolte et al., 2004), the result is an ordinary coherence (Schlögl and Brunner, 2008). We will refer to it as coherence.
- **Complex coherence:** By considering the imaginary part of the complex-valued coherence (Nolte et al., 2004), we get complex coherence.
- **Partial coherence:** This is the partial coherence, calculated with an alternative method as provided in the biosig-toolbox. Partial coherence, also known as Gersch causality, was first designed to identify epileptic foci by Gersch and Goddard (1970). The authors proposed that one channel is said to drive the other channels if the first channel explains or accounts for the linear relation between the other two. The real part of the partial coherence was used.
- **Partial directed coherence:** Partial directed coherence as an extended concept of partialized coherence, is a measure of the relative strength of the direct interaction between pairs of regions (Baccalá and Sameshima, 2001).
- **Partial directed coherence factor:** The partial directed coherence factor (Baccalá and Sameshima, 2001) is an intermediate step between partial coherence and partial directed coherence. It adds directionality to partial coherence, but includes instantaneous causality, which is undesirable when examining processes that evolve over time such as an epileptic seizure (Schuster and Kalliauer, 2009).
- **Generalized partial directed coherence:** The major advantage of generalized partial directed coherence (Baccalá et al., 2007) over partial directed coherence is its robustness against scaling differences between the signals (Taxidis et al., 2010).
- **Directed transfer function:** Like directed coherence, directed transfer function represents information that flows from one region to another over many possible alternative pathways (Kaminskí and Blinowska, 1991).
- **Direct directed transfer function:** The direct directed transfer function extends the concept of directed transfer function by distinguishing between direct and indirect causal relations of signals (Korzeniewska et al., 2003). As such, the concepts of partial coherence and directed transfer function are combined.
- **Full frequency directed transfer function:** The difference between the directed transfer function and the full frequency directed transfer function (Korzeniewska et al., 2003) is that the directed transfer function is normalized by the total frequency content of the considered frequency band, while the full frequency directed transfer function is normalized with respect to all the frequencies in the predefined frequency interval. As such, the full frequency directed transfer function priorizes those frequencies which contribute the most to the power of the signal (van Mierlo et al., 2011).
- **Geweke's Granger Causality:** This is a modified version of Geweke's Granger Causality (Geweke, 1982), concretely the bivariate version as in Bressler et al. (2007).

### 2.9. Measuring test-retest reliability

We decided not to use the parametric intra-class-correlation to measure the test-retest reliability, but to perform a non-parametric Spearman correlation (such as in Fein et al., 1983; Gasser et al., 1985; Salinsky et al., 1991) because we did not want to impose a model assuming a linear relation between measurements. We measured reliability by Spearman rank correlation between the two times of registration for each measure of interaction and for each of the 60 participants, across the Cartesian product of all frequency × electrode × electrode combinations (or electrode × electrode combinations for direct causality). Thus, for each participant and each of the variations of data discontinuity, data length, model order, frequency averaging, and each individual frequency band we obtained one correlation coefficient.

The results were reported descriptively because the number of moderators and measures would result in a multiple comparisons problem, the correction of which would have caused a very low power of the study. Thus, the distribution of the correlation coefficients was assessed descriptively with boxplots over the variation of the moderators of interest. The boxplots represent the range between the first and the third quartile as a box alongside with the median (red line in the middle of the box), and the whiskers are drawn to ±2.7σ, that is 99.3% coverage and extended to the adjacent value, which is the most extreme data value that is not an outlier. Outliers are represented as red crosses and defined as values that are greater than *q*_3_+1.5 · (*q*_3_−*q*_1_) where *q*_*i*_ is the *i*th quartile.

## 3. Results

### 3.1. Sample

Table 1 gives an overview of the demographic characteristics of patients included in the subgroups.

**Table 1:** Sample overview.

**Group**	***N***	**Age**	***w* (%)**	***r* (%)**	**MMSE/MOCA**	**MAT**	**MOS**	**ZAN**	**BDI**
MCI	22	68.5 (48–76)	11 (50)	21 (95)	28.5 (25–30)	110 (80–125)	100 (80–125)	97.5 (80–135)	7 (0–21)
SCC	5	57.0 (52–74)	2 (40)	5 (100)	28.5 (27–30)	110 (100–115)	95 (90–130)	105 (95–125)	14 (4–21)
TLEr	6	33.5 (21–51)	3 (50)	5 (93)	–	97.5 (80–110)	95 (85–105)	87.5 (80–95)	9 (0–27)
TLEl	7	55.0 (36–66)	6 (86)	7 (100)	–	95 (70–105)	85 (65–115)	95 (80–110)	10 (0–20)
HC	20	61.5 (23–74)	14 (70)	18 (90)	28 (26–30)	115 (85–130)	120(75–135)	115 (90–140)	3 (0–15)

*N, Number; w, number of women; r, number of right-handed participants; MMSE, mini mental state examination; employed only in patients with MCI and SCC; MOCA, Montreal cognitive assessment—employed only in HC subgroup; Wechsler subscales MAT, matrices; MOS, mosaics; ZAN, repeat numbers; BDI, Beck Depression Inventory; age and neuropsychological values are given as median with (range); MCI, mild cognitive impairment; SCC, subjective cognitive complaints; TLEr/TLE l, right/left lateralized temporal lobe epilepsy; HC, healthy controls*.

### 3.2. Effect of artifacts

First, we compared test-retest reliability calculated over the whole dataset (complete data, that is, 123.5 s) with the artifact-free data, that is, the dataset when artifacts were excluded. The complete data variant included artifacts but could be assumed to be continuous, while the artifact-free data variant is discontinuous because of the excluded artifacts. For this purpose, measures were averaged over frequency ranges before calculation of reliabilities.

**Figure 2:**
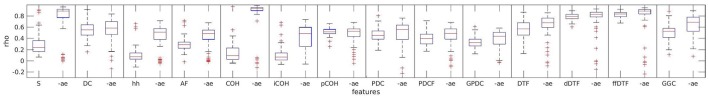
Effect of artifacts and artifact exclusion on test-retest reliability of the assessed measures. For each measure two boxplots indicate the complete data and artifact-free data variant, respectively. “*-ae”* indicates the boxplot of the respective measure when artifacts were excluded. S, spectrum; DC, direct dausality; hh, transfer function polynomial; AF, transfer function; COH, real valued coherence; iCOH, imaginary part of complex valued coherence; pCOH2, partial coherence; PDC, partial directed coherence; PDCF, partial directed coherence factor; GPDC, generalized partial directed coherence; DTF, directed transfer function; dDTF, direct directed transfer function; ffDTF, full frequency directed transfer function; GGC, Geweke's Granger causality.

As we can see from Figure 2, for most measures, excluding artifacts increased test-retest reliability. This effect was very strong for spectrum and coherence. For measures such as direct directed transfer function and full frequency directed transfer function test-retest reliability was high in both cases, with and without artifacts.

### 3.3. Effect of artifact-free data length

Table 2 shows the correlation values of a Spearman correlation between the test-retest reliabilities and the number of artifact-free segments, averaged across the two sessions. We can see that the relations vary considerably between measures. Bonferroni correction requires *p*-values to be below 0.0036, which yields significant positive correlation for transfer function, partial coherence, generalized partial directed coherence, directed transfer function and full frequency directed transfer function. These measures show a higher reliability with increasing number of artifact-free segments.

**Table 2:** Spearman correlation coefficients for the relationship between test-retest reliability based on artifact-free data and included number of artifact-free segments.

**Measure**	**rho**	***p*-value**
Spectrum	0.36	0.005
Direct causality	−0.16	0.23
Transfer function	0.45	**0.0003**
Transfer function polynomial	0.29	0.02
Real valued coherence	0.30	0.02
Complex coherence	0.28	0.03
Partial coherence	0.53	**0.00001**
Partial directed coherence	0.12	0.36
Partial directed coherence factor	0.10	0.44
Generalized partial directed coherence	0.43	**0.0007**
Directed transfer function	−0.01	0.95
Direct directed transfer function	0.50	**0.00004**
Full frequency directed transfer function	0.37	**0.0032**
Geweke's Granger causality	−0.04	0.78

*Number of artifact-free segments was averaged across two sessions. P-values are uncorrected. Bold font indicates significance at the Bonferroni-corrected level p < 0.0036*.

### 3.4. Effects of discontinuity vs. length

We present the effects of discontinuity alongside with the modification of data length, in order to demonstrate descriptively which of the two effects is larger. We found that the measures exhibit different modulation patterns, so that we picked three measures which seem to be exemplary for the three types of modulations.

**Figure 3:**
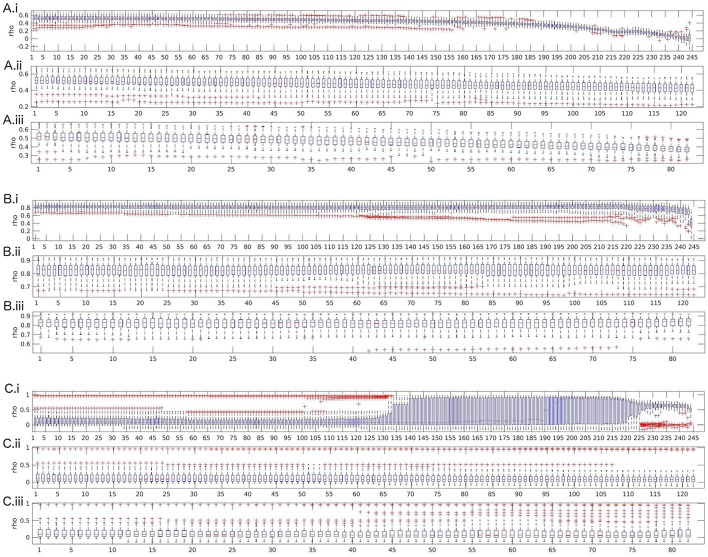
Boxplots of test-retest reliabilities for partial coherence **(A)**, full frequency directed transfer function **(B)**, and real-valued coherence **(C)**. **(i)** Length variation by successively shortening data by segments of size 500 ms (250 samples). **(ii)** 0.5 s cut-outs: successively leaving out every second segment of size 500 ms (250 samples). **(iii)** 1 s cut-outs: successively leaving out every second segment of size 1,000 ms (500 samples). The x-axis represents the number of removed segments.

Figure 3 shows the effects of length and discontinuity variation on reliability for partial coherence, full frequency directed transfer function, and real-valued coherence.

Partial coherence shows an expected pattern of strong and monotonic decrease of reliability beginning quite early over the course of leaving out segments, with first signs visible around 70 left-out segments and a strong decrease after 160 segments. The other two cut-out variants show the same effect, visible as a decrease in reliability which is almost parallel to the decrease of reliability over variation of data length. Thus, discontinuity does not additionally reduce reliability, the main moderator is data length.

The second possible pattern of reliability changes is demonstrated in the example of full frequency directed transfer function. For full frequency directed transfer function, shortening of the data decreases reliability after removing more than approximately 220 segments. That is, only when the data length is shorter than about 13 s, reliability is affected. Indeed, this corresponds approximately to the signal length when the ratio between number of samples and coefficients to estimate drops below 1 (13 · 500)/(27 · 250) = 0.96). The outliers change after removing more than 120 segments, indicating that for single subjects the effect of data length may be stronger than for others. The other two cut-out variants (middle and bottom subplot) show no strong variation. Overall, full frequency directed transfer function seems to be quite reliable as long as a few seconds are left for calculation.

For real valued coherence the effect is somewhat surprising. Variability increases after more than about 125 segments were left out, that is, when about 61 s remain, and reaches a maximum when more than 140 segments were left out. After leaving out more than 190 segments, the mean reliability across subjects rises from reliabilities around 0.1 to values close to 1. The variance becomes smaller along with a slight decrease of reliability again toward the end of the variation, that is, when more than 225 segments were removed, that is, when about 11 s remain for analysis. For coherence, discontinuity has no remarkable effect on the anyway low reliability values. Only a few single outliers show higher reliability when leaving more segments out, which might be an analogous increase in variability across subjects such as when shortening the data.

The figures for all other measures are included in the Supplementary Material. A similar pattern as for real-valued coherence, that is, increase of variability and reliability when reducing the data length by 130 to 200 segments and decrease toward the very end, can be found for spectrum, transfer function (but with a short and low increase of reliability, only), complex coherence, and Geweke's Granger causality. A similar pattern as for full frequency directed transfer function, that is, a decrease when data length becomes very short, can be found for direct causality, partial directed coherence, partial directed coherence factor, directed transfer function, and direct directed transfer function. A similar pattern as for partial coherence with a very strong decrease, visible also for the other two variants of variation, can be found for transfer function polynomial and generalized partial directed coherence.

### 3.5. Model order and frequency averaging

The effects of model order and frequency averaging are very small in contrast to the effect of artifact exclusion. In order to demonstrate this we present figures including the variation of model order, frequency averaging and exclusion of artifacts.

Two observations can be made across all measures. Most measures show a tendency toward lower reliability when no frequency averaging is performed. However, when excluding artifacts affects reliability of a measure, this effect is more prominent than the effect of frequency averaging. Basically, we can observe two different behaviors among the measures.

**Figure 4:**
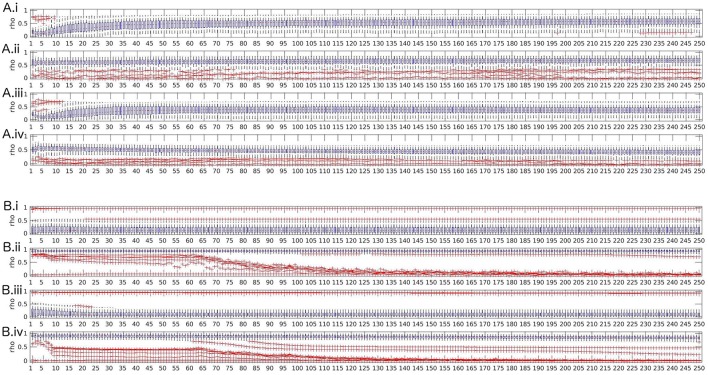
Boxplots of test-retest reliabilities vs. model order for for directed transfer function **(A)** and real valued coherence **(B)**. **(i)** top row: complete data (with artifacts), with frequency averaging; **(ii)** second row: artifact-free data, with frequency averaging; **(iii)** third row: complete data without frequency averaging; **(iv)** bottom row: artifact-free data, without frequency averaging.

The most frequent behavior was found for transfer function, partial coherence, partial directed coherence, generalized partial directed coherence, partial directed coherence factor, Geweke's Granger causality and all variants of directed transfer function (direct and full frequency), where reliability decreases for model orders 1–5 and then increases up to model order 30 when artifacts are included; for excluded artifacts, there is only little change in the lower model orders, but without frequency averaging there is a small decrease of reliability over the whole range of increasing model orders. Figure 4A illustrates this pattern with the exemplary measure directed transfer function.

Spectrum, real-valued coherence, and complex coherence exhibit almost no effects of model order, despite a slight decrease of reliability over model orders 1–20 when artifacts are included and no frequency averaging was performed (see Figure 4B for real-valued coherence as an example). The Figures for the other measures are given in the Supplementary Material.

In summary, we suggest that the model order that is most suitable for obtaining reliable values is high, but should allow that the ratio *N*/(*M* · *p*) is large, as well. Model orders below 5 are unstable for some measures, but when the model order approached half of the sampling rate, the reliability dropped down, as well, which might be an effect of the reduced ratio between available samples *N* for model estimation and number of parameters to estimate *M* · *p* (model order times channels).

### 3.6. Reliability within frequency bands

In order to document whether the classical frequency bands differed between each other with respect to reliability, we calculated reliability for every frequency range separately, without averaging across frequencies, on the artifact-free data.

**Figure 5:**
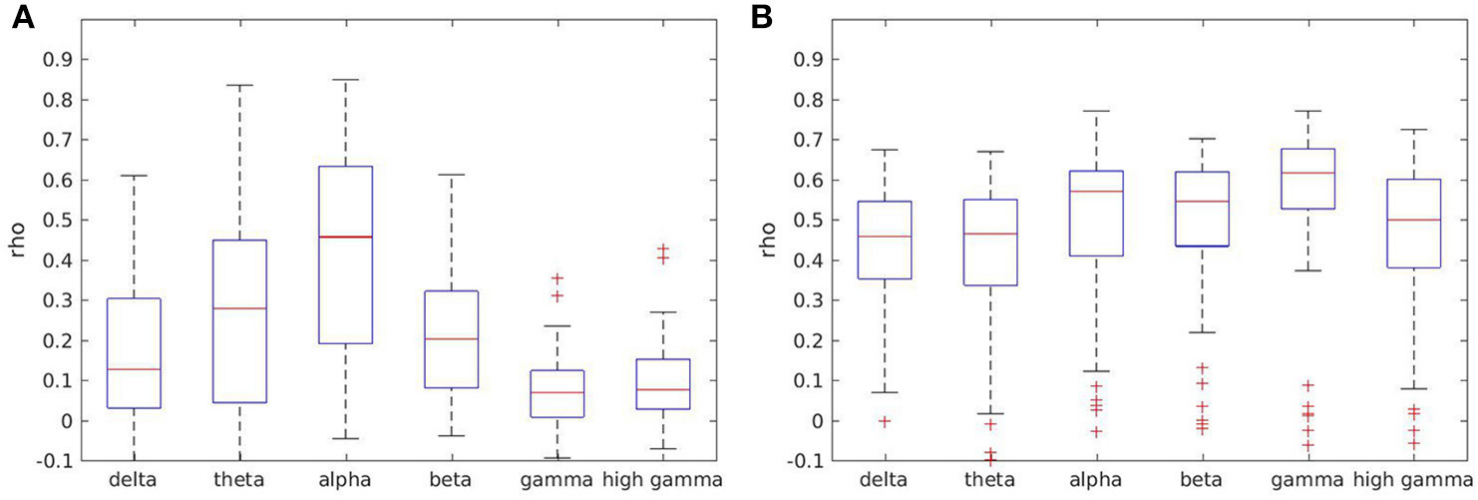
Boxplots of test-retest reliabilities for coherence **(A)** and full frequency directed transfer function **(B)** in separated frequency ranges.

Most measures showed higher reliability for lower frequencies theta and alpha, and lower reliability for gamma ranges. However, reliability in the delta range was low for some measures such as coherence, as well (see Figure 5A). For other measures such as full frequency directed transfer function this trend could not be found, with a quite similar distribution across frequency ranges (see Figure 5B).

Boxplots for all other measures except direct causality, which is not frequency dependent, are given in the Supplementary Material.

### 3.7. Relation between pathology and data length dependent reliability

**Figure 6:**
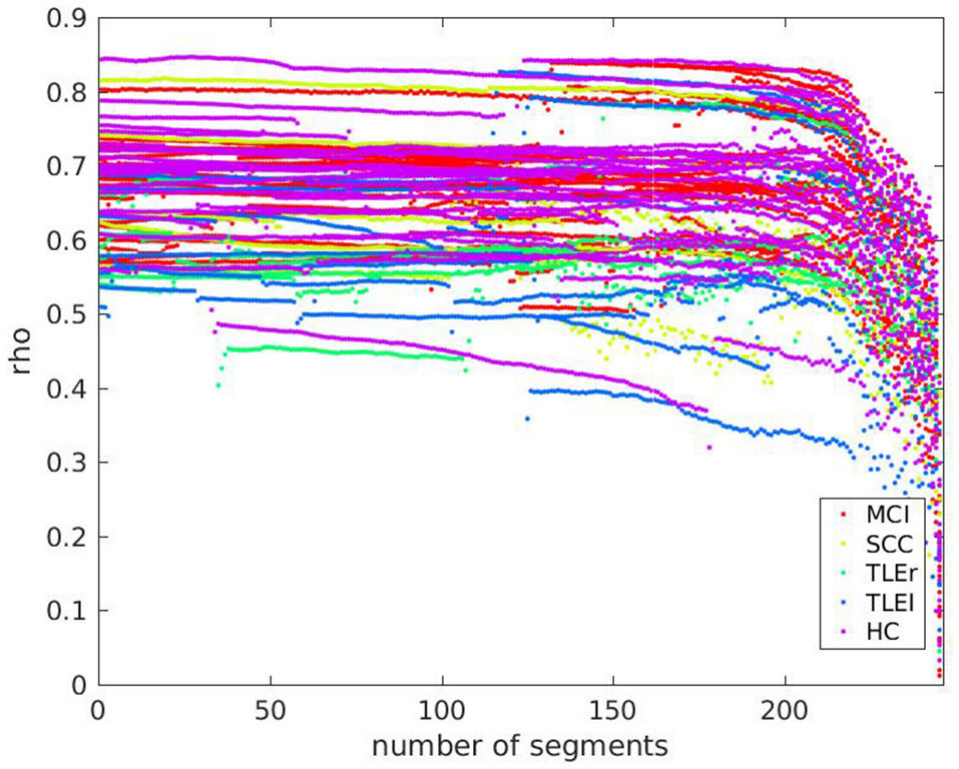
Scatter plot of test-retest reliabilities for full frequency directed transfer function vs. number of excluded segments of size 500 ms. The x-axis represents the number of segments that were cut out from the end of the signal. Dots represent values of individual participants. Colors indicate groups MCI, mild cognitive impairment; SCC, subjective cognitive complaints; TLEr, temporal lobe epilepsy with focus on the right hemisphere; TLEl, temporal lobe epilepsy with focus on the left hemisphere; HC, healthy controls.

Since the variations of data disruption by leaving out segments (cut-out variations 4 and 5) did not show an effect on reliability, we present here only the effects of data length in relation to pathology. We prepared scatter plots representing the course of reliability over the length of the signal used for calculation of measures and colored the dots according to the neurological populations. Basically, the scatter plots represent what can be seen in the box-plots from Section 3.4. As an example, we include the scatter plot of full frequency directed transfer function in Figure 6. The dots represent the pathological groups, so that we can see whether there is a differential pattern across groups. Some of the patients with TLE can be found on the lower range of the distribution. However, this observation does not interact with signal length.

**Figure 7:**
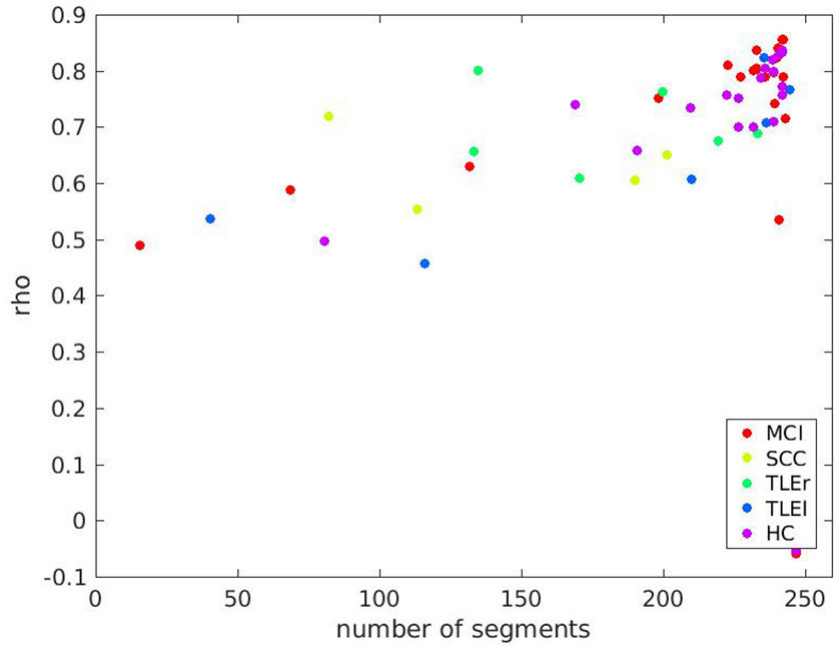
Scatter plot of test-retest reliabilities for full frequency transfer function vs. number of clean segments of size 500 ms. Calculation of test-retest reliabilities was based on segments without artifacts, only. The x-axis represents the average number of segments across two sessions included in the analysis. Colors indicate groups MCI, mild cognitive impairment; SCC, subjective cognitive complaints; TLEr, temporal lobe epilepsy with focus on the right hemisphere; TLEl, temporal lobe epilepsy with focus on the left hemisphere; HC, healthy controls.

A scatter plot for relation between reliability based on clean trials with number of clean trials for full frequency directed transfer function as given in Figure 7 confirms the linear trend as statistically characterized in Table 2. However, the relation between reliability and trial numbers is quite parallel across groups.

Scatter plots for all other measures are shown in the Supplementary Material. In sum, there are differences between groups so that we suggest that studies comparing different groups of participants by some measure of interaction should always report alongside an estimate of reliability. This procedure should ensure that the reported group differences are not merely due to fluctuations that are stronger in the one or the other group.

## 4. Discussion

What affects test-retest reliability of measures of interaction? As suggested by previous publications (David et al., 2004; Braun et al., 2012) our results confirm that the most important factor is the measure itself. Depending on this choice, other factors may or may not play a role. A qualitative overview of how the assessed moderators affect reliability of the examined measures of interaction is summarized in Table 3.

**Table 3:** Qualitative overview of relevance of moderators for reliability of measures.

**Measure**	**~ Reliability**	**Artifacts**	**Data length**	**Discontinuity**	**Model order**	**Frequency**	**Pathology**
Spectrum	0.2–0.9	+	+	−	−	+	−
Direct causality	0.6	−	−	−	+		−
Transfer function	0.3−0.5	+	+	−	+	o	−
Transfer function polynomial	0.1−0.5	+	o	−	+	o	+
Real valued coherence	0.1−1	+	o	−	+	+	o
Complex coherence	0.1−0.5	+	o	−	−	+	o
Partial coherence	0.5	−	+	−	+	o	+
PDC	0.5	−	−	−	o	−	o
PDC factor	0.4	−	−	−	+	−	−
Generalized PDC	0.4	−	+	−	+	−	+
DTF	0.7	o	−	−	+	−	−
Direct DTF	0.8	−	+	−	+	−	+
Full frequency DTF	0.9	−	+	−	o	−	+
Geweke's Granger causality	0.5−0.7	+	−	−	+	−	−

*PDC, Partial directed coherence; DTF, directed transfer function; +, highly relevant; o, moderate; −, low relevance*.

In comparison to related work, the varying reliability between measures is highly plausible. Albeit other studies did not examine exactly the same measures, it is worth to mention that Hardmeier et al. (2014) reported test-retest reliability between 0.12 and 0.80 using the phase locking index, depending on which frequency band was examined and depending on the level of integration (local/global). A similar variance in reliability was reported only in our own recent study (Höller et al., 2017); but it should be noted that no other study on reliability involved a comparably large set of measures and factors that affect reliability (Deuker et al., 2009; Jin et al., 2011; Miskovic and Keil, 2015).

We found quite stable and high reliability for variants of the directed transfer function, especially the full frequency directed transfer function. Indeed, for many applications full frequency directed transfer function could be considered a good choice, but there are some issues that should be considered when selecting it. For example, the reliability drops when the signals get shorter. This is most likely an effect of the poor adjustment between signal length and model order, since we kept the model order constant when varying signal length. Thus, it should be first examined in future studies whether a lower model order in designs with shorter EEG segments, for instance, event-related designs, would lead again to a high reliability of full frequency directed transfer function.

### 4.1. Data length

As for fMRI (Altmann et al., 2016), we would expect that longer signals lead to higher reliabilities, which was true for most but not for all of the examined measures. The highly reliable measures direct and full frequency directed transfer function drop in reliability when the signals become very short (<13 s). This corresponds approximately to the signal length when the ratio between number of samples and coefficients to estimate drops below 1 (13 · 500)/(27 · 250) = 0.96) so that the estimation of the model results in an underdetermined problem. In contrast, reliability of coherence is higher with shorter segments, which seems plausible since coherence does actually not depend on model order. Chu et al. (2012) found that stable networks, that is, reliably replicable networks could be identified with the cross-spectrum and real valued coherence across signal lengths of 100 s. Measures such as coherence can be estimated on short signal lengths such as individual trials of cognitive tasks, with reliabilities well above those of phase-dependent measures (Miskovic and Keil, 2015).

By definition, a long signal is not required for non-directed measures such as coherence, because they do not take the past of the signal into account. Therefore, signal length is more important for directed measures, because estimation of the model relies on a sufficient number of samples. A tradeoff between model order and signal length needs to be considered when short signals are of interest. In contrast, for non-directed measures a shorter signal is better, because a longer signal increases the chance for a large variation of the signal over the course of the recording, which in turn, might result in overall unreliable results.

### 4.2. Artifacts

In general, reliability of most directed measures was more robust against artifacts than reliability of non directed measures such as coherence or spectrum. We could speculate that directed measures are robust against artifacts, because artifacts occur at irregular points in time. Thus, they do only contribute as noise to the model estimation, but not alter the result *per se*. In contrast, the estimation of power always includes also irregular activity such as artifacts, heavily influencing measures such as coherence and spectrum which strongly reflect power characteristics of the signal. Another potential explanation is that directed measures may show a tendency toward the mean, and therefore exhibit less fluctuation, which in turn makes them less affected by artifacts. However, both of these explanations cannot fully explain our results since for example Geweke's Granger causality showed considerable increase of reliability when artifacts were excluded. Future simulation studies could help to characterize the relation between directionality of measures, reliability, artifacts, and signal length.

We could speculate that the way artifacts are identified and removed could seriously affect those measures of interaction that are more susceptible to artifacts. Various methods are available for artifact correction, but it seems that exclusion of artifacts is still the best choice for analysis of connectivity (van Diessen et al., 2015). Especially the use of independent component analysis may introduce spurious similarity between the signals due to the back-transformation with exclusion of noisy signals. This makes it extremely interesting, that some of the assessed measures are robust against artifacts in terms of reliability.

### 4.3. Frequency bands

Depending on the measure of interest, some frequency bands are less reliable. Some measures show higher reliability in the frequency ranges around the alpha range and lower reliability in the delta and gamma range. Our data suggest that averaging the measures within specified frequency ranges increases reliability for some measures.

Previous studies documented a high variance between subjects along with high test-retest reliability within subjects over long time ranges of absolute power in traditional frequency bands (Gasser et al., 1985; Dustman et al., 1999; Grandy et al., 2013; Näpflin et al., 2016). Hatz et al. (2015b) reported test-retest reliability across three annual EEG recordings with intraclass correlation coefficients. Lower frequencies (delta, theta) were slightly less reliable than high frequencies (alpha, beta). Our results for measures such as coherence resemble very much the reliability of phase locking indices as reported by Hardmeier et al., with lower reliability in theta and beta frequency band and higher reliability along with higher variability in the alpha range (Hardmeier et al., 2014). The potential effect of the frequency band needs to be taken into account when choosing the measure of interaction for the analysis and when results in different frequency ranges are compared to each other. Moreover, averaging within frequency ranges is highly recommended because it seems to lead to more reliable results.

The full-frequency directed transfer function is more robust against frequency effects, because the normalization technique included in the formulation of this measure takes into account the variation between the sub-bands. The interaction within each frequency sub-band is normalized by the content of all sub-bands (Korzeniewska et al., 2003). This method prioritizes the sub-band which contributes most band-power to the signal. It is a nice coincidence that the alpha range is not only the sub-band contributing mostly to the power of the signal, but in addition it is also the most reliable sub-band. Therefore, the reliability of all sub-bands benefits from the high reliability in the alpha range.

### 4.4. Pathology

So-called microstates are an effective way of differentiating subnetwork activity by accounting for stationarity and thus, increasing the reliability of the detected networks (Khanna et al., 2014). Indeed, only when accounting for stationarity within subnetworks, the difference between the stable group of patients with mild cognitive impairment and progressive group with Alzheimer's disease became evident (Hatz et al., 2015a). While measures of interaction are frequently implemented as biomarkers in studies involving clinical populations, the reliability of the results is only rarely reported. We replicated the previously documented differences of reliability between patients with temporal lobe epilepsy and healthy controls (Höller et al., 2017), which was also dependent on the measure of interaction. In the present work we could show that this effect did not interact with data length. However, we addressed no other interactions of the potential moderators with pathology, because this was already addressed in a previous study. As reported previously, the differences in reliability between pathological groups are specific to regions and frequency ranges, and these differences vary across measures (Höller et al., 2017).

Differences between patient groups may be inconsistent across studies due to poor reliability of measures of interactions rather than the differences between the patients themselves. Future research should consider this aspect when selecting the measures of interest. For example, we found that measures that are highly reliable such as the full frequency directed transfer function differ in reliability between the examined populations. That is, when comparing populations by means of this measure it should be considered that differences could also be due to a difference in reliability between the groups.

Nevertheless, when searching for differences between pathological groups, it might not always be advantageous to select the most reliable measure, if this measure is insensitive to specific characteristics of the investigated patients. Moreover, even if coherence is highly sensitive to differences between neurological populations and even if it is reliable at short-term intervals, this measure is highly affected by volume conduction, which reduces the neurobiological significance of the revealed interactions. On the other hand, the directed measures are supposed to unravel the direction of information flow between the assessed regions—in the sense of a time-lagged similarity of the signals. This allows to examine the propagation dynamics of events of interest, such as epileptic seizures or interictal epileptiform events. Nevertheless, seizures are typically EEG recordings that are highly contaminated by artifacts, which poses specific demands on the choice of the measure, highlighting the advantage of measures such as direct and full frequency directed transfer function, which were found to be quite robust against artifacts in the signal.

### 4.5. Model order

Most studies in the literature use a fixed model order as we did for discontinuity variation. We varied model order across the scenarios of frequency averaging and artifact exclusion. Varying model order alongside with discontinuity variation was not done for reasons of computational complexity and because we could already see that the major effect is not discontinuity *per se* but the measure of interest, signal length and the exclusion of artifacts. Model order can be defined by several criteria, but it was recommended to choose the maximum possible model order (Schlögl and Supp, 2006), which is exactly what we have implemented in the present paper when we varied discontinuity. Another possibility is to determine the optimal model order according to the Akaike information criterion (Kaminski et al., 2001; Babiloni et al., 2005). However, the estimation of the MVAR model is strongly related to model order with respect to the oscillations that can be represented by the model order. Obviously, a lower model order emphasizes higher frequencies, because the model is not able to capture the full length of slow oscillations. In contrast, a high model order might cover the whole range of frequencies of interest, but the model order is limited by the number of data samples that are needed to estimate the model. A high model order most likely negatively impacts reliability when the number of to be estimated parameters exceeds the available samples.

### 4.6. Limitations

A potential confounder for reliability is volume conduction. Volume conduction is *per-se* highly reliable, since the structural properties of the brain stay the same unless a brain lesion, surgical intervention, or severe atrophy cause changes at a larger scale. Thus, measures of interaction that are susceptible to volume conduction might result in artificially high reliability. Obviously, volume conduction in the EEG is problematic when assessing brain networks (Christodoulakis et al., 2015). Volume conduction and activity at the reference can lead to artificial high coherence values. Imaginary coherency (Nolte et al., 2004) and partial coherence (Gersch and Goddard, 1970) solve the problem only to some extent. Directional measures, namely directed coherence (Saito and Harashima, 1981) and directed transfer function (Kaminskí and Blinowska, 1991), partial directed coherence (Baccalá and Sameshima, 2001), direct directed transfer function (Korzeniewska et al., 2003), full frequency directed transfer function (Korzeniewska et al., 2003), and generalized partial directed coherence (Baccalá et al., 2007) are considered to support the estimation of propagating networks, but can not fully avoid the bias of volume conduction. Volume conduction should be quite stable across time, so that we can assume that those measures being more sensible to volume conduction may yield higher reliability than e.g., imaginary coherence, which is being claimed to be less affected by volume conduction (Nolte et al., 2004). As a phase-dependent measure, imaginary coherence makes strong assumptions about stationarity of the signal, which may, in turn, be the reason why reliability of these measures is so poor. Nevertheless, correlation of the phase locking index with inter-electrode distance yielded considerable correlation coefficients of −0.19 for the unweighted, and −0.4 for the weighted variant of this measure in high-density EEG (Hardmeier et al., 2014). This correlation may partly explain the high test-retest reliability values reported in this study in terms of intraclass correlation coefficients of up to 0.79. That is, in high-density EEG volume conduction contributes significantly to measures of interaction and, most likely, to their reliability, even if they are phase-dependent. The situation might be a bit different when looking at magnetoencephalography, which should be less affected by volume conduction effects. Mutual information between wavelet coefficients yielded graph theoretical characteristics that were highly reliable (Deuker et al., 2009).

The volume conduction problem could be solved by assessing signals at the source level, that is, by implementing blind source separation techniques (Gomez-Herrero et al., 2008). However, source space brings different problems along such as the non-existence of a unique solution and still, even in source space the problems of field spread and volume conduction are not completely solved (van Diessen et al., 2015).

Somewhat related to volume conduction, also the choice of the reference might be a factor that should be considered. We chose to re-reference against linked-earlobes, since the common average reference is unlikely to approximate a zero sum reference in low-density EEG recordings as in the present study (Schiff, 2005; Nunez and Srinivasan, 2006). There is no unique recommendation for the choice of the reference. To what extent the choice of classical or more sophisticated references such as infinity reference, Laplacian, or mitigation of the influence of neural activity in the common average reference are related to estimation of connectivity is unclear (van Diessen et al., 2015), and the effect of the reference on reliability needs to be determined in future studies.

Moreover, the subsamples drawn from neurological populations were quite small, so that conclusions for the individual populations are limited. In addition, the group of healthy controls was drawn from students, as well as elderly people that attended the University 50+ programme, an offer of the University of Salzburg for people with the desire to attend University classes for selected topics of interest. It is possible that this interest in learning and the high education of the control group caused a general bias.

It is of interest that even with a very reliable measure such as the full frequency directed transfer function and a long data segment, there are participants with a very low reliability. The outliers in the bottom right corner of Figure 7 include participants from almost all groups. The reason for this poor reliability in this sample of participants remains unclear. However, we suppose that factors that we did not control for might play a role, such as duration and quality of sleep in the night before the recording, amount of mind wandering during rest, consumption of caffeine or tobacco, etc. Future studies addressing these factors may explain outliers such as those found in the present study.

A further aspect that deserves more attention in the future is the interval between the EEG measurements. The interval implemented in this study was 2 weeks, that matches exactly one phase of the female cycle of 4 weeks. It is of interest whether future studies could systematically examine sex-differences with respect to test-retest reliability, since it is known that the menstrual cycle affects resting state alpha frequency Broetzner et al. (2014). Moreover, the typical length of neurorehabilitative therapies or follow-up examinations on medication acting upon the central nervous system should be considered. Two weeks might seem a bit short in view of the longer-term effects of therapeutical programs.

Finally, task related electroencephalographic recordings are suggested to be more reliable than resting-state recordings, since the cognitive activity during a resting state cannot be controlled sufficiently (McEvoy et al., 2000; van Diessen et al., 2015). Therefore, future studies should examine the reliability of measures of interaction during cognitive tasks.

In sum, this work addressed a considerable number of potential moderators of test-retest reliability. The results were reported descriptively because the number of moderators and measures would have caused a multiple comparisons problem, which was unfeasible to be corrected. Nevertheless, there are several other putative moderators that leave room for further investigations in future studies.

## 5. Conclusions

In this study we could demonstrate that in addition to the choice of the measure, signal length affects reliability of measures of interaction, while discontinuity of the signal has no effect. Exclusion of artifacts is relevant for reliability of most non-directed measures such as coherence, but not for most directed measures such as direct, full frequency, or classical directed transfer function. Similarly, differences between reliability within frequency ranges is moderated by the choice of the measure. As shown previously, reliabilities differ between patients with different neurological conditions, but these differences do not interact with discontinuity or signal length, nor with artifact exclusion. Model order is relevant for most measures within the examined range of model orders. Choosing a high model order is recommendable, but the design should be limited by calculating the ratio between available samples and to-be estimated parameters is highly recommended. We suggest that the future of brain network research should be guided by a paradigm shift in order to fight the reproducibility crisis. Scientists should argue the choice of measures of interaction to be used for a specific study by considering the factors that affect reliability.

## Author contributions

YH performed the analysis and wrote the manuscript. AU, AB, and JF supervised the work in technical and statistical respects and contributed ideas to how the analysis should be performed and how the results should be presented. AT and KB performed data acquisition. KB performed data preprocessing. AT contributed the initial idea that led to this work. ET and RN supervised the work in clinical respects.

### Conflict of interest statement

The authors declare that the research was conducted in the absence of any commercial or financial relationships that could be construed as a potential conflict of interest.
