# Supplementary material for: Reliability of EEG Measures of Interaction: A Paradigm Shift Is Needed to Fight the Reproducibility Crisis
Source: Front Hum Neurosci. 2017 Aug 30;11:441. doi: 10.3389/fnhum.2017.00441 (PMC5582168; doi:10.3389/fnhum.2017.00441)
Supplement: Supplementary file 1 [file DataSheet1.PDF]

Supplementary Material: Reliability of EEG measures of interaction: a paradigm shift is needed to fight the reproducibility crisis

Yvonne Höller [1*], Andreas Uhl [2], Arne Bathke [3], Aljoscha Thomschewski [1,4], Kevin Butz [1,4], Raffaele Nardone [1,4,5], Jürgen Fell [4], and Eugen Trinka [1,4]

*Correspondence:
Yvonne Höller
y.hoeller@salk.at

Table S1 **Demographic data and clinical findings on the hippocampus from structural MRI**

nr	group	age	hand	sex	MRI
1	MCI	74	r	f	left: mild hippocampal atrophy
2	MCI	73	r	m	bilateral hippocampal atrophy
3	MCI	71	r	f	bilateral mild/moderate hippocampal atrophy
4	MCI	63	r	m	normal
5	MCI	76	r	m	bilateral moderate atrophy, left>right
6	MCI	72	r	m	bilateral severe atrophy
7	MCI	61	r	m	bilateral moderate atrophy, left>right
8	MCI	64	r	m	normal
9	MCI	72	r	f	normal
10	MCI	48	r	m	normal
11	MCI	62	r	m	left: hippocampal malrotation
12	MCI	60	r	f	normal
13	MCI	64	r	m	normal
14	MCI	70	r	f	mild bilateral hippocampal atrophy
15	MCI	65	r	m	normal
16	MCI	51	r	f	normal
17	MCI	74	r	f	left: mild atrophy
18	MCI	71	r	m	bilateral atrophy right>left
19	MCI	50	r	m	normal
20	MCI	71	r	m	n.a.
21	MCI	68	r	f	normal
22	MCI	56	r/l	f	n.a.
23	SCC	56	r	f	left: moderate hippocampal atrophy
24	SCC	69	r	m	normal
25	SCC	57	r	m	bilateral minor hippocampal atrophy
26	SCC	74	r	m	bilateral minor hippocampal atrophy, right>left
27	SCC	52	r	f	normal
28	TLEr	51	r	m	right: hippocampal sclerosis
29	TLEr	20	l	m	left: mild hippocampal sclerosis
30	TLEr	49	r	f	left: hippocampal malrotation
31	TLEr	37	r	f	left: mild hippocampal sclerosis
32	TLEl	65	r	f	left: large tumor
33	TLEl	54	r	f	left: hippocampal sclerosis
34	TLEl	59	r	m	right: hippocampal atrophy
35	TLEl	47	r	f	normal
36	TLEr	28	r	f	right: hippocampal sclerosis
37	TLEl	57	r	f	normal
38	TLEl	38	r	f	normal
39	TLEl	36	r	f	left: severe hippocampal atrophy; right: mild hippocampal atrophy
40	TLEr	27	r	m	oligodendroglioma grade II, right mesial

Continued on next page

nr	group	age	hand	sex	MRI
41	HC	67	r	f	bilateral mild hippocampal atrophy
42	HC	32	r	f	normal
43	HC	66	r	m	bilateral mild hippocampal atrophy, left>right
44	HC	61	r	m	bilateral mild hippocampal atrophy
45	HC	52	r	f	normal
46	HC	66	r	f	left: hippocampal malrotation
47	HC	74	r	m	normal
48	HC	24	r	m	bilateral mild hippocampal atrophy; right>left
49	HC	33	r	m	normal
50	HC	67	r	f	normal
51	HC	45	r	f	normal
52	HC	62	r	f	normal
53	HC	26	r	m	normal
54	HC	23	r	f	normal
55	HC	72	r/l	f	bilateral mild hippocampal atrophy
56	HC	64	r	f	left: mild hippocampal malrotation
57	HC	60	r	f	bilateral hippocampal atrophy, severe cortical atrophy
58	HC	58	r	f	left: mild hippocampal malrotation
59	HC	74	l	f	n.a.
60	HC	64	r	f	normal

m=male; f=female; hand=handedness; r=right; l=left; n.a. = information not available
MCI= mild cognitive impairment; SCC=subjective cognitive complaints; TLEr=right-lateralized temporal lobe epilepsy; TLEl=left lateralized TLE; HC=healthy controls

Table S2 **Details about the patients with temporal lobe epilepsy included in this study.**

nr	side	loc	type	seizure
28	r	mesial	focal S/C	no
29	r	mesial	focal S/C, FTSG	n.a.
30	r	nd	focal S/C, FTSG	no
31	r	mesial	focal C, FTSG	n.a.
32	l	mesial	focal S/C, FTSG	no
33	l	mesial	focal S	yes
34	l	nd	focal C, FTSG	no
35	l	anterior mesial	focal S/C, FTSG	n.a.
36	r	nd	focal S	no
37	l	nd	focal C	no
38	l	mesial	focal C, FTSG	no
39	l	anterior	FTSG	no
40	r	mesial	focal C, FTSG	no

nr= number; m=male; f=female; side= side of TLE; r=right; l=left;
hand= handedness; loc= localization; type= seizure type
seizure=seizures within 24h before/after EEG;
n.a. = information not available; nd= not defined
S = simple (without loss of consciousness);
C= complex (with loss of consciousness)
FTSG= focally triggered secondary generalized tonic-clonic seizure

Table S3 **Self-reported medications of participants.**

nr	group	general	anti-epileptic drugs	psycho-pharmacological drugs
1	MCI	Simvastin 40mg 1; Enahexal Comp 1; Calcivit 2xweek	0	0
2	MCI	Cerebokan 80mg 1-0-1	0	0
3	MCI	Niften mite 1-0-1	0	0
4	MCI	Bezastad 200mg 1-0-0, Ascalan 4mg 1/2-1/2-1/2, Iterium 1mg 1-0-0, Amlodipin 5mg 1-0-1, Nomexor 5mg 1-0-0, Candasaromp 16/12.5mg 1-0-0	0	0
5	MCI	Concor 1/2-0-1/2; Metformin 850mg; Simvastatin 80mg 1/2; Alna 0.4mg; Marcomar, Furadantin 1-0-1	0	0
6	MCI	0	0	0
7	MCI	Simvastatin 20mg every 2 days	0	0
8	MCI	0	0	0
9	MCI	Sintrom 3/4, Sotacor 1, Mencord plus 1, Doxazosin 1	0	0
10	MCI	Lisinopril 20/25mg	0	0
11	MCI	Thrombo AS 1, Co Renitec 1x, Glucophage 1x	0	0
12	MCI	Ibandronacid	0	0
13	MCI	Spiriva 1x, Foster 2x, Thrombo AS 1x, Amlodipin 1x	0	0
14	MCI	Euthyrox 0.75	0	Sifrol 0.35
15	MCI	Concor 2.5mg 1-0-0	0	0
16	MCI	Concor 2.5mg 1-0-1	0	0
17	MCI	Sirdalud 4 mg 1x abends, Voltaren 50mg rapid, Cerebokan 80mg 1-0-1	0	0

Continued on next page

nr	group	general	anti-epileptic drugs	psycho-pharmacological drugs
18	MCI	Amlodipin 5mg, Simvastatin 20mg, Thrombo AS, Methohexal 47.5mg, Cerebokan 80mg	0	Sanoten 10mg
19	MCI	0	0	0
20	MCI	Diovan 1x, Co-Diovan 1x	0	0
21	MCI	Acecomb 1x, Atorvastatin 1x, Cerebokan 2x	0	0
22	MCI	0	0	0
23	SCC	Euthyrox 100mg 1/2-0-3/4; folic acid, b-vitamins	0	Johanicum
24	SCC	Acecomb semi 1x	0	0
25	SCC	0	0	0
26	SCC	Carvediol Hexal 25mg 0-0-1, Diamicron Mr 30mg 2-0-0, Lisinopril Int 20mg 1-0-0, Lisinopril Hct 25mg 1-0-0, Metformin Rtp 850mg 1-1-1, Simvastatin 30mg 0-0-1, Zanipril 10/20mg 0-0-1	0	0
27	SCC	Voltaren 150mg when necessary	0	0
28	TLEr	0	Keppra 2x	0
29	TLEr	0	Vimpa 200mg 1-1, Lamotrigin 100mg 1-1, Lamotrigin 50mg 0-1	0
30	TLEr	0	Trileptal 600mg 1/2-1/2-1	0
31	TLEr	Ibumetin forte 400mg when necessary	Keppra 1000mg 1-0-1, Vimpat 100mg 1-0-1	0
32	TLEl	Euthyrax 1-0-0	Keppra 100mg/500mg jew. 1-0-1, Lamictal 100mg 1-1, Vimpat 100mg 1-1	0
33	TLEl	Thrombostad 100mg	Keppra 3000mg, Lamotrigin 174mg	Trittico 100mg
34	TLEl	0	Keppra 2-0-2	Lyrica 150mg 1-0-2, Lyrica 75mg 1-0-0
35	TLEl	0	Keppra 1 1/2-0- 1 1/2, Lamictal 125-150mg, Vimpat 200mg 1-1	0

Continued on next page

nr	group	general	anti-epileptic drugs	psycho-pharmacological drugs
36	TLEr	Folsan 1-0-0	Keppra 1000mg 1-0-1, Keppra 500mg 0-0-1, Gerolamic 200mg 1-0-1, Gerolamic 100mg 1-0-1	0
37	TLEl	0	Levetiracetam 500mg 2-0-2	0
38	TLEl	0	Keppra 1000mg 1-1,	Nootrop 600mg 1 1/2- 1 1/2
39	TLEl	Mexalen 500mg 1-1-1	Levebon 500mg 2-0-2; Fycompa 2mg 0-0-1;	Halcion 0.25mg 0-0-0-1
40	TLEr	0	Zonegran 150mg	Cannabis
41	HC	Omeprazol 20mg, Sintrom 1/2, Nomexor 1, Ramipril 1, Bezafibrat, Thyrex 1	0	0
42	HC	oral contraception	0	0
43	HC	Losartan, Losartan HCT, Torasemid, Jodthyrox, Thrombostad	0	0
44	HC	0	0	0
45	HC	Thyrex	0	0
46	HC	Dorzastad 1-0-1, Parkemed when necessary 0-4	0	0
47	HC	Thrombostad 1/2,	0	0
48	HC	0	0	0
49	HC	0	0	0
50	HC	0	0	0
51	HC	n.a.		
52	HC	Euthyrox 100mg 1-0-0, Nomexor 1/2-0-0, Zanipril 0-0-1	0	0
53	HC	0	0	0
54	HC	Euthyrox 75mg	0	0
55	HC	n.a.		
56	HC	Urbason 1-1-1	0	0
57	HC	Lisinopril 2x 1/2, Simvastatin 0-0-1	0	0
58	HC	Thyrex 50mg	0	0
59	HC	Co-Diavan 80mg+12.5mg 1-0-0, Rivacor 10mg 1-0-0, Thrombostad 0-1-0, Allostad 1-0-0, Ezetrol 1-0-0, Pentoxi 400mg	0	0

Continued on next page

nr	group	general	anti-epileptic drugs	psycho-pharmacological drugs
60	HC	Diabetex 500mg 2-0-2, Thyrex 100mg 1/2-0-0, Galvus 50mg 1-0-1	0	0

MCI= mild cognitive impairment; SCC=subjective cognitive complaints; TLEr=right-lateralized temporal lobe epilepsy; TLEl=left lateralized TLE; HC=healthy controls

Table S4 **Clinical evaluation of the EEGs of all participants included in this study.**

nr	group	EEG1 awake	base	clinical	EEG2 awake	base	clinical
1	MCI	yes	10	no	yes	10	no
2	MCI	yes	10	no	yes	10	no
3	MCI	yes	13	no	yes	13	no
4	MCI	yes	11	no	yes	11	no
5	MCI	yes	10	no	yes	10	FS δ T8
6	MCI	yes	10	no	yes	10	FS θ F4
7	MCI	wake N1; vertexwaves; alpha-dropout	13	no	wake N1; vertexwaves; alpha-dropout	13	no
8	MCI	wake-N1	9	no	yes	9	no
9	MCI	wake-N1; alpha-dropout	10	FS θ F7	yes	10	FS θ F7
10	MCI	yes	10	FS δ P8, P7	yes	10	FS δ P8, P7
11	MCI	yes	10	FS δ F7-T7, T8	yes	10	FS δ F7-T7, T8
12	MCI	yes	10	no	yes	10	no
13	MCI	yes	11	no	yes	11	no
14	MCI	yes; alpha-dropout	11	FS θ F7	yes; alpha-dropout	11	FS θ F7
15	MCI	yes; alpha-dropout	9	no	yes; alpha-dropout	9	no
16	MCI	yes	10	FS δ T7, T8	yes	10	FS δ T7, T8
17	MCI	yes	10	FS θ F7, F8	yes	10	FS θ F7, F8
18	MCI	yes; alpha-dropout	11	no	yes; alpha-dropout	11	no
19	MCI	yes; alpha-dropout	9	FIRDA	yes; alpha-dropout	9	FIRDA
20	MCI	yes	10	no	yes	10	no
21	MCI	yes; alpha-dropout	11	FS δ-θ F7-T7, F8	yes; alpha-dropout	11	FS δ-θ F7-T7, F8
22	MCI	yes	12	FS δ F7, F8	yes	12	FS δ F7, F8
23	SCC	yes	11	-	yes	11	-
24	SCC	yes	10	-	yes	10	-
25	SCC	yes	11	FS δ-θ F7-T7, F8	yes	11	FS δ-θ F7-T7, F8
26	SCC	yes	13	FS θ F8	yes	13	FS θ F8
27	SCC	yes; alpha-dropout	10	FS θ T8	yes	10	FS θ T8
28	TLEr	yes	10	no	yes	10	no
29	TLEr	yes	10	FS θ F4-F8	yes	10	FS θ F4-F8

Continued on next page

nr	group	EEG1			EEG2		
		awake	base	clinical	awake	base	clinical
30	TLEr	yes	9	no	yes	9	no
31	TLEr	yes	10	repetitive sharp-waves F8-T8: 1.5-2/s	yes	10	no
32	TLEl	yes	10	no	yes	10	no
33	TLEl	yes	10	no	yes	10	no
34	TLEl	yes	13	no	yes	13	no
35	TLEl	yes	10	no	yes	10	no
36	TLEr	yes	10	FS δ F8	yes	10	FS δ F8
37	TLEl	yes	9	no	yes	9	breach T7
38	TLEl	yes	9	no	yes	9	no
39	TLEl	wake-N1	11	no	yes	11	no
40	TLEr	yes	12	no	wake-N1	12	no
41	HC	yes	10	no	yes	10	no
42	HC	yes	10	no	yes	10	no
43	HC	yes	10	no	yes	10	no
45	HC	yes	10	no	yes	10	no
45	HC	wake-N1	13	no	yes	13	no
46	HC	yes	9	no	wake-N1	9	no
47	HC	yes	10	no	yes	10	no
48	HC	yes	11	no	yes	11	no
49	HC	yes	11	no	yes	11	no
50	HC	yes	11	no	yes	11	no
51	HC	yes	10	no	yes	10	no
52	HC	wake N1; vertexwaves; alpha-dropout	12	FS δ-θ T7, T8	yes	12	FS δ-θ T7, T8
53	HC	yes	10	no	wake N1; vertexwaves; alpha-dropout	10	no
54	HC	yes	10	no	yes	10	no
55	HC	yes	11	FS θ T8	yes	11	FS θ T8
56	HC	wake; alpha-dropout	13	FS δ-θ T7, T8	yes	13	FS δ-θ T7, T8; ictal patter 3s T8
57	HC	yes	10	no	yes	10	no
58	HC	yes	10	no	yes	10	no
59	HC	yes	9	FS θ F7-T7	yes	9	FS θ F7-T7
60	HC	yes	11	FS δ-θ T7	yes	11	FS δ-θ T7

Continued on next page

**nr**	**group**	**EEG1**			**EEG2**		
		**awake**	**base**	**clinical**	**awake**	**base**	**clinical**

EEG1/2= results from clinical evaluation of the first and second EEG recording; MCI= mild cognitive impairment; SCC=subjective cognitive complaints; TLEr=right lateralized temporal lobe epilepsy; TLEl=left lateralized TLE; HC=healthy controls; awake = wakefulness/ sleep signs or stage; FS = focal slowing; FIRDA = frontal intermitted rhythmic delta activity

1 TEST-RETEST RELIABILITY AFFECTED BY SEGMENT LENGTH AND DISCONTINUITY

The effect of the variation of segment length and discontinuity on test-retest reliability of the assessed measures of interaction is shown in Figures S1 to S11. These data are based on the measures with frequencies averaged in the 6 selected classical frequency bands delta (1-4 Hz), theta (5-7 Hz), alpha (8-13 Hz), beta (14-30 Hz), gamma (31-80 Hz), and high gamma (81-125 Hz) before calculation of reliabilities.

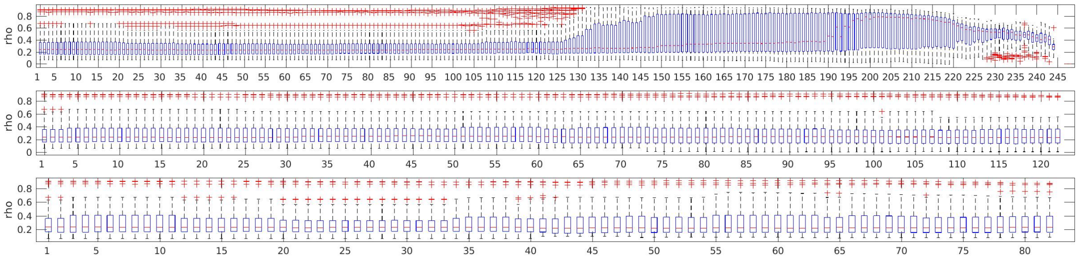

Figure S1. Boxplots of test-retest reliabilities for spectrum. Top: successively shortening data by segments of size 500msec (i.e., 250 samples). Middle: successively leaving out every second segment of size 500msec (i.e., 250 samples). Bottom: successively leaving out every second segment of size 1000msec (i.e., 500 samples). The x-axis represents the number of retained segments.

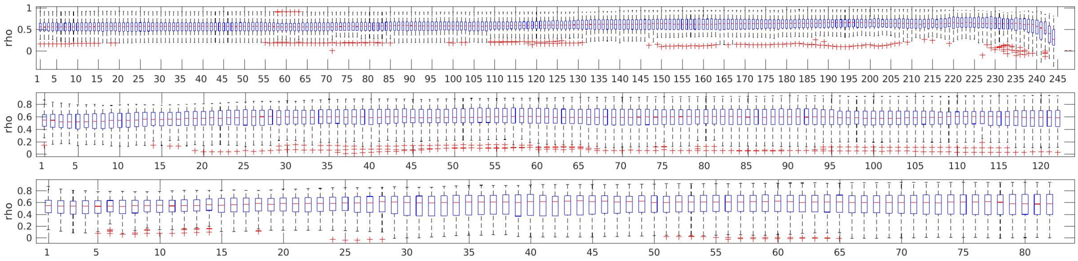

Figure S2. Boxplots of test-retest reliabilities for direct causality. Top: successively shortening data by segments of size 500msec (i.e., 250 samples). Middle: successively leaving out every second segment of size 500msec (i.e., 250 samples). Bottom: successively leaving out every second segment of size 1000msec (i.e., 500 samples). The x-axis represents the number of retained segments.

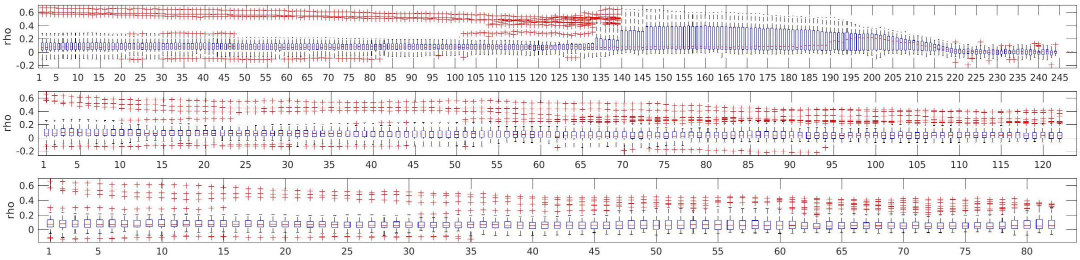

Figure S3. Boxplots of test-retest reliabilities for transfer function. Top: successively shortening data by segments of size 500msec (i.e., 250 samples). Middle: successively leaving out every second segment of size 500msec (i.e., 250 samples). Bottom: successively leaving out every second segment of size 1000msec (i.e., 500 samples). The x-axis represents the number of retained segments.

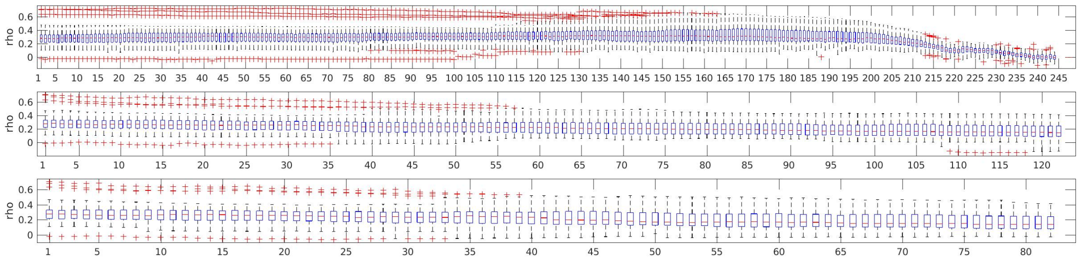

Figure S4. Boxplots of test-retest reliabilities for transfer function polynomial. Top: successively shortening data by segments of size 500msec (i.e., 250 samples). Middle: successively leaving out every second segment of size 500msec (i.e., 250 samples). Bottom: successively leaving out every second segment of size 1000msec (i.e., 500 samples). The x-axis represents the number of retained segments.

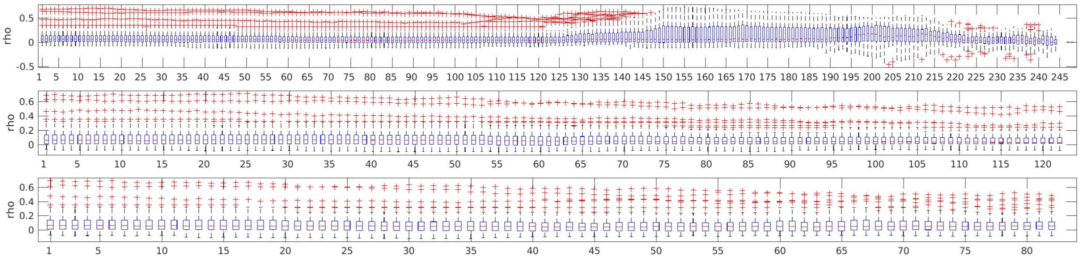

Figure S5. Boxplots of test-retest reliabilities for complex coherence. Top: successively shortening data by segments of size 500msec (i.e., 250 samples). Middle: successively leaving out every second segment of size 500msec (i.e., 250 samples). Bottom: successively leaving out every second segment of size 1000msec (i.e., 500 samples). The x-axis represents the number of retained segments.

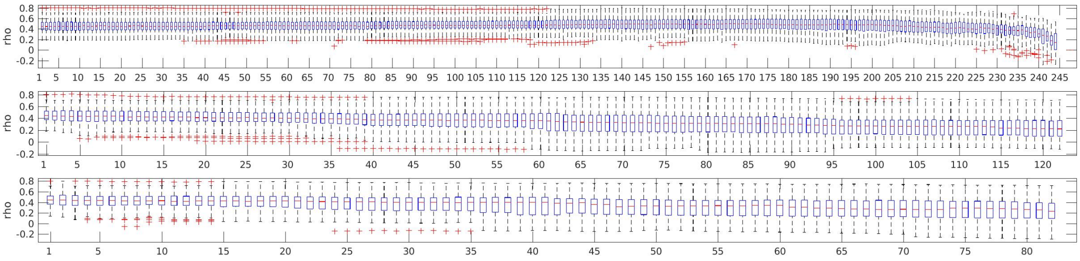

Figure S6. Boxplots of test-retest reliabilities for partial directed coherence. Top: successively shortening data by segments of size 500msec (i.e., 250 samples). Middle: successively leaving out every second segment of size 500msec (i.e., 250 samples). Bottom: successively leaving out every second segment of size 1000msec (i.e., 500 samples). The x-axis represents the number of retained segments.

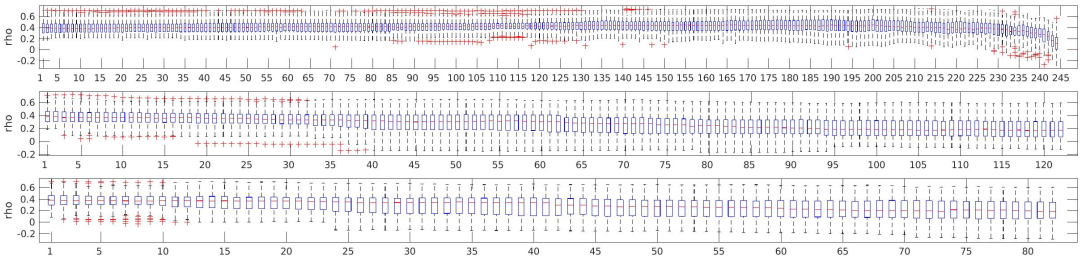

Figure S7. Boxplots of test-retest reliabilities for partial directed coherence factor. Top: successively shortening data by segments of size 500msec (i.e., 250 samples). Middle: successively leaving out every second segment of size 500msec (i.e., 250 samples). Bottom: successively leaving out every second segment of size 1000msec (i.e., 500 samples). The x-axis represents the number of retained segments.

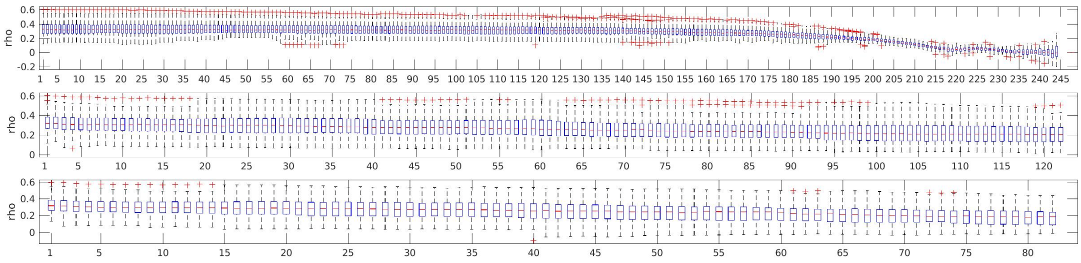

Figure S8. Boxplots of test-retest reliabilities for generalized partial directed coherence. Top: successively shortening data by segments of size 500msec (i.e., 250 samples). Middle: successively leaving out every second segment of size 500msec (i.e., 250 samples). Bottom: successively leaving out every second segment of size 1000msec (i.e., 500 samples). The x-axis represents the number of retained segments.

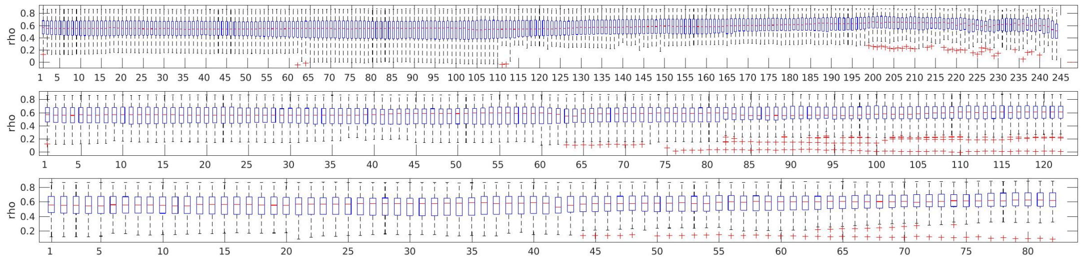

Figure S9. Boxplots of test-retest reliabilities for directed transfer function. Top: successively shortening data by segments of size 500msec (i.e., 250 samples). Middle: successively leaving out every second segment of size 500msec (i.e., 250 samples). Bottom: successively leaving out every second segment of size 1000msec (i.e., 500 samples). The x-axis represents the number of retained segments.

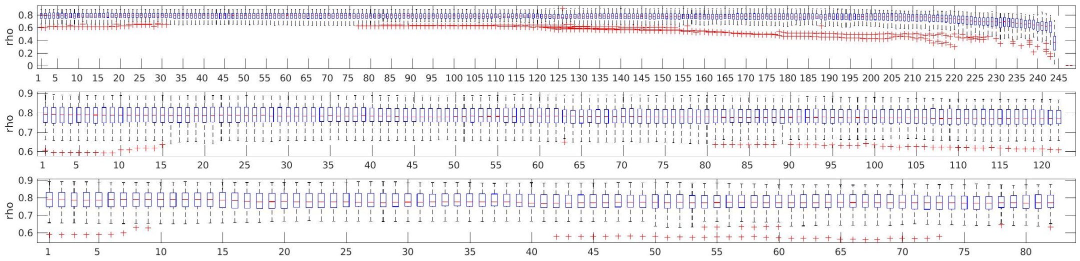

Figure S10. Boxplots of test-retest reliabilities for direct directed transfer function. Top: successively shortening data by segments of size 500msec (i.e., 250 samples). Middle: successively leaving out every second segment of size 500msec (i.e., 250 samples). Bottom: successively leaving out every second segment of size 1000msec (i.e., 500 samples). The x-axis represents the number of retained segments.

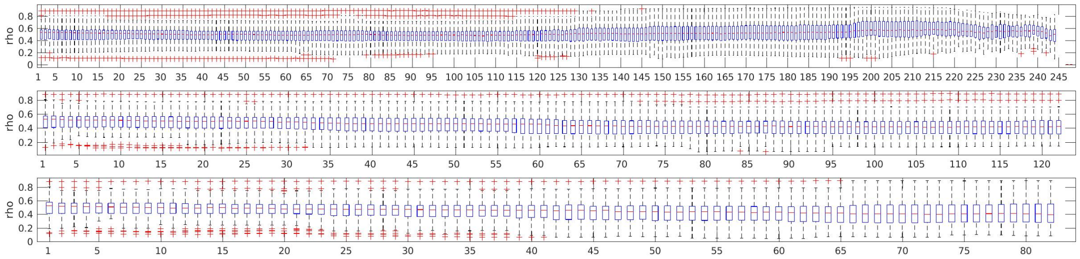

Figure S11. Boxplots of test-retest reliabilities for Geweke's Granger causality. Top: successively shortening data by segments of size 500msec (i.e., 250 samples). Middle: successively leaving out every second segment of size 500msec (i.e., 250 samples). Bottom: successively leaving out every second segment of size 1000msec (i.e., 500 samples). The x-axis represents the number of retained segments.

2 MODEL ORDER AND FREQUENCY AVERAGING

The effect of the variation of model order and frequency averaging on test-retest reliability of the assessed measures of interaction is shown in Figures S12 to S23. These data are based on the measures with or without averaging the values in the 6 selected classical frequency bands delta (1-4 Hz), theta (5-7 Hz), alpha (8-13 Hz), beta (14-30 Hz), gamma (31-80 Hz), and high gamma (81-125 Hz) before calculation of reliabilities.

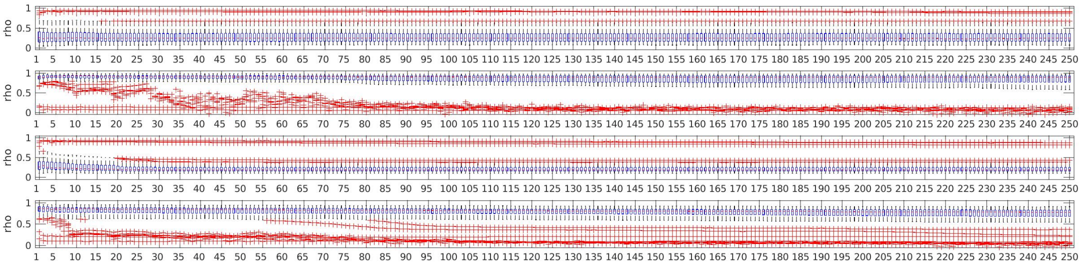

Figure S12. Boxplots of test-retest reliabilities for spectrum vs. model order for i) top row: with artefacts, with frequency averaging; ii) second row: without artefacts, with frequency averaging; iii) third row: with artefacts without frequency averaging; iv) bottom row: without artefacts, without frequency averaging.

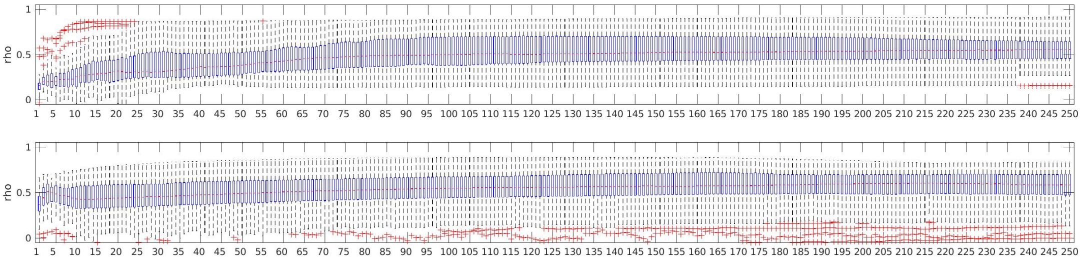

Figure S13. Boxplots of test-retest reliabilities for direct causality vs. model order for i) top row: with artefacts, with frequency averaging; ii) second row: without artefacts, with frequency averaging; iii) third row: with artefacts without frequency averaging; iv) bottom row: without artefacts, without frequency averaging.

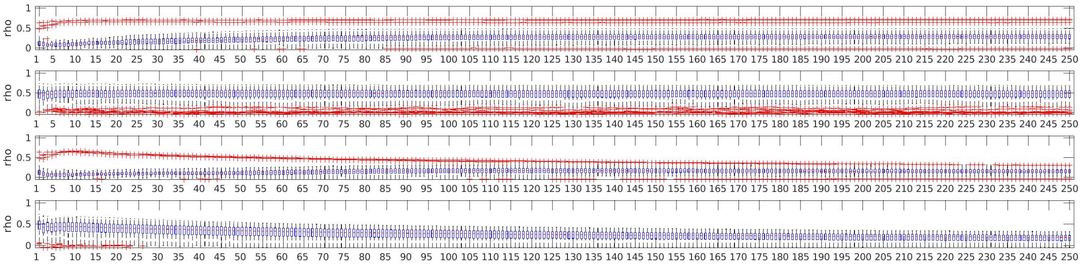

Figure S14. Boxplots of test-retest reliabilities for transfer function vs. model order for i) top row: with artefacts, with frequency averaging; ii) second row: without artefacts, with frequency averaging; iii) third row: with artefacts without frequency averaging; iv) bottom row: without artefacts, without frequency averaging.

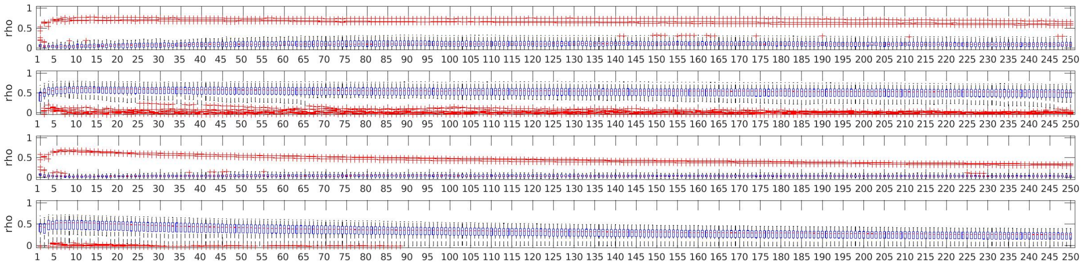

Figure S15. Boxplots of test-retest reliabilities for transfer function polynomial vs. model order for i) top row: with artefacts, with frequency averaging; ii) second row: without artefacts, with frequency averaging; iii) third row: with artefacts without frequency averaging; iv) bottom row: without artefacts, without frequency averaging.

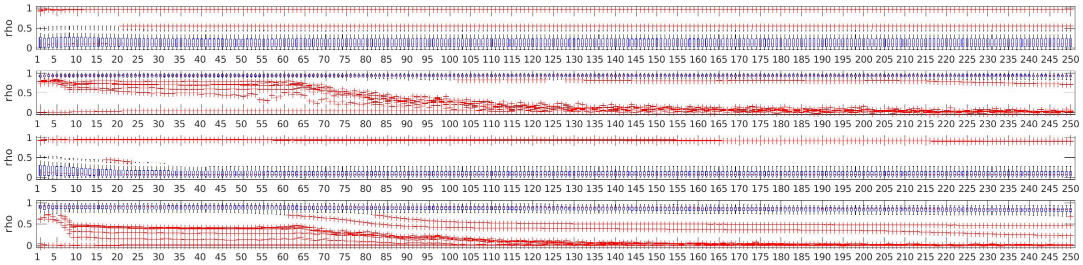

Figure S16. Boxplots of test-retest reliabilities for complex coherence vs. model order for i) top row: with artefacts, with frequency averaging; ii) second row: without artefacts, with frequency averaging; iii) third row: with artefacts without frequency averaging; iv) bottom row: without artefacts, without frequency averaging.

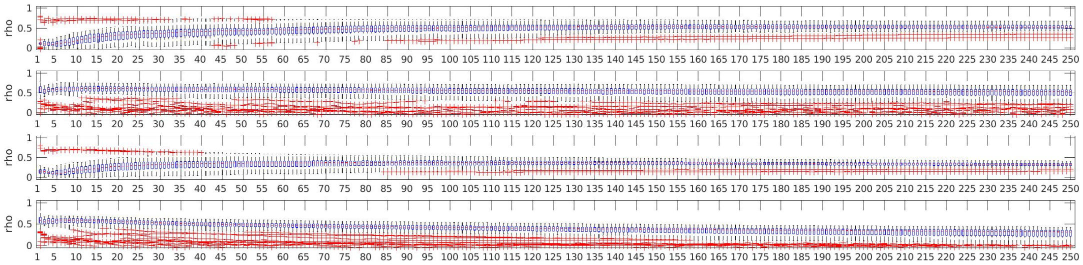

Figure S17. Boxplots of test-retest reliabilities for partial coherence vs. model order for i) top row: with artefacts, with frequency averaging; ii) second row: without artefacts, with frequency averaging; iii) third row: with artefacts without frequency averaging; iv) bottom row: without artefacts, without frequency averaging.

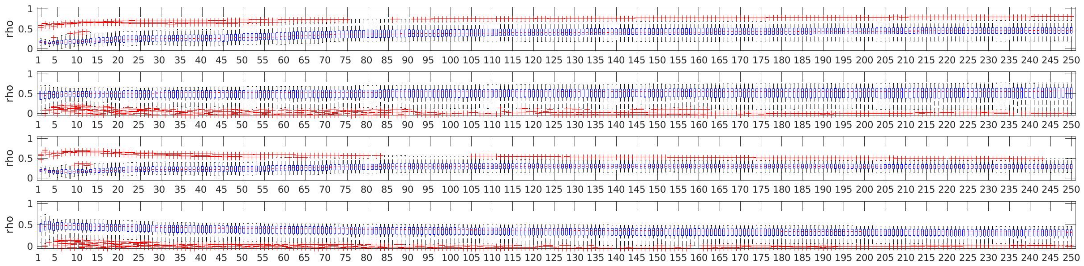

Figure S18. Boxplots of test-retest reliabilities for partial directed coherence vs. model order for i) top row: with artefacts, with frequency averaging; ii) second row: without artefacts, with frequency averaging; iii) third row: with artefacts without frequency averaging; iv) bottom row: without artefacts, without frequency averaging.

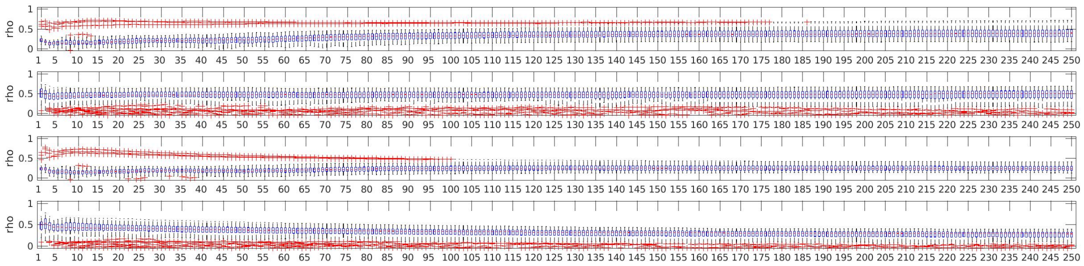

Figure S19. Boxplots of test-retest reliabilities for partial directed coherence factor. vs. model order for i) top row: with artefacts, with frequency averaging; ii) second row: without artefacts, with frequency averaging; iii) third row: with artefacts without frequency averaging; iv) bottom row: without artefacts, without frequency averaging.

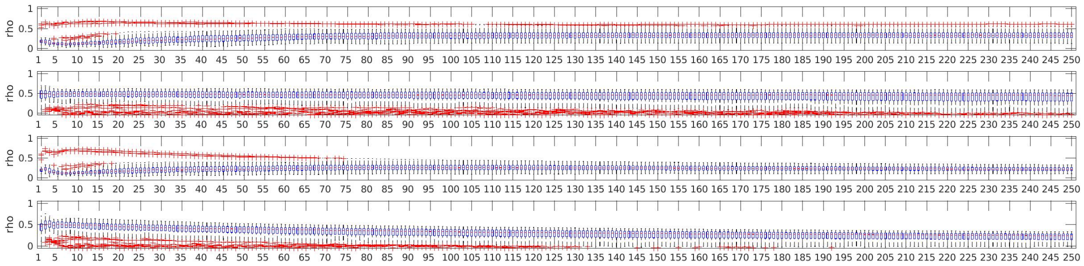

Figure S20. Boxplots of test-retest reliabilities for generalized partial directed coherence vs. model order for i) top row: with artefacts, with frequency averaging; ii) second row: without artefacts, with frequency averaging; iii) third row: with artefacts without frequency averaging; iv) bottom row: without artefacts, without frequency averaging.

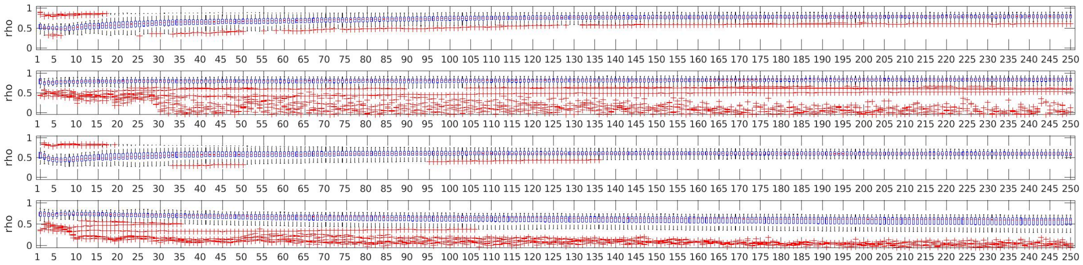

Figure S21. Boxplots of test-retest reliabilities for direct directed transfer function vs. model order for i) top row: with artefacts, with frequency averaging; ii) second row: without artefacts, with frequency averaging; iii) third row: with artefacts without frequency averaging; iv) bottom row: without artefacts, without frequency averaging.

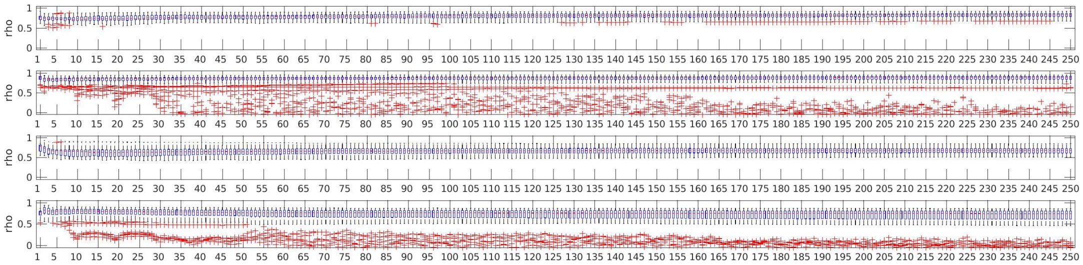

Figure S22. Boxplots of test-retest reliabilities for full frequency directed transfer function vs. model order for i) top row: with artefacts, with frequency averaging; ii) second row: without artefacts, with frequency averaging; iii) third row: with artefacts without frequency averaging; iv) bottom row: without artefacts, without frequency averaging.

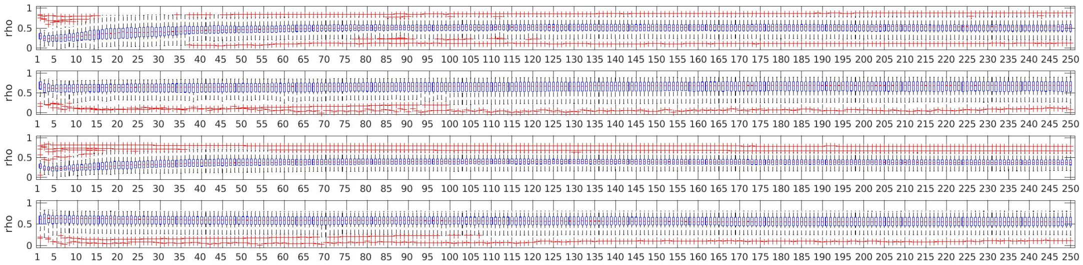

Figure S23. Boxplots of test-retest reliabilities for Geweke's Granger causality vs. model order for i) top row: with artefacts, with frequency averaging; ii) second row: without artefacts, with frequency averaging; iii) third row: with artefacts without frequency averaging; iv) bottom row: without artefacts, without frequency averaging.

3 RELIABILITY WITHIN FREQUENCY BANDS

In order to document whether the classical frequency bands differed between each other with respect to reliability we calculated reliabilities within each frequency range, without frequency averaging. We show these results for classical frequency ranges delta (1-4 Hz), theta (5-7 Hz), alpha (8-13 Hz), beta (14-30 Hz), gamma (31-80 Hz), and high gamma (81-125 Hz) in Figures S24 to S34.

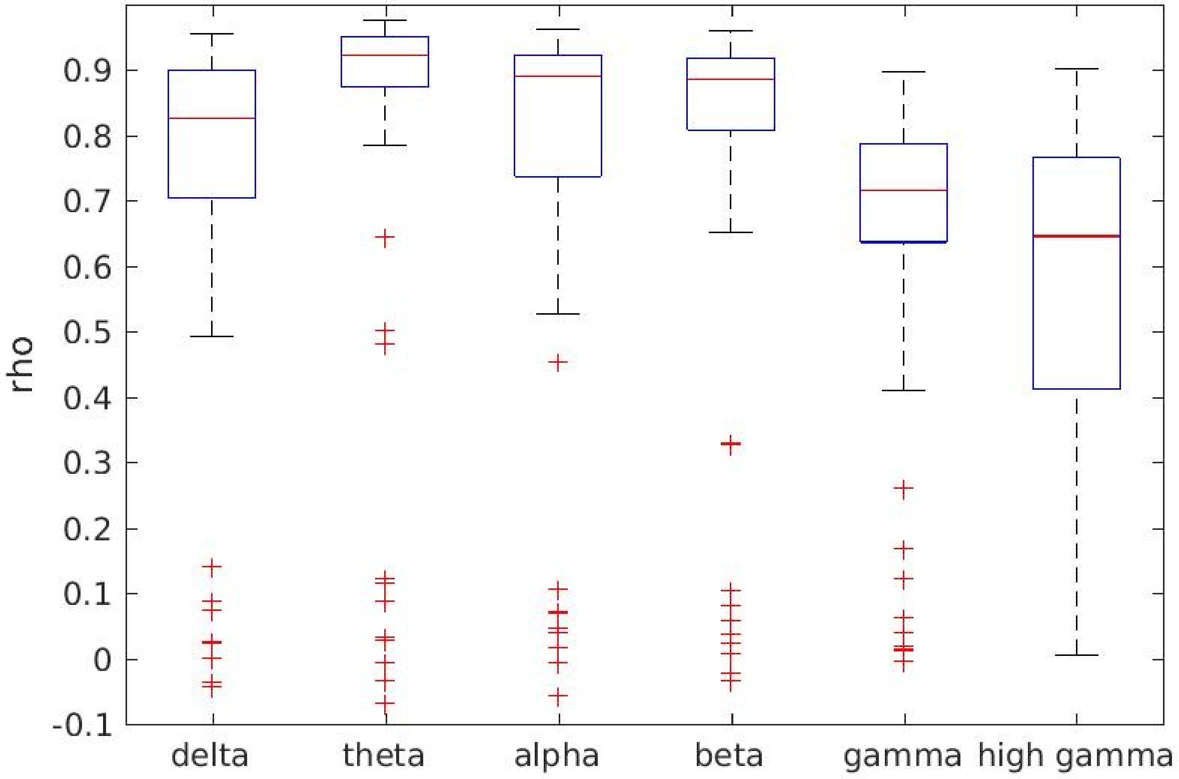

Figure S24. Boxplots of test-retest reliabilities for spectrum in separated frequency ranges.

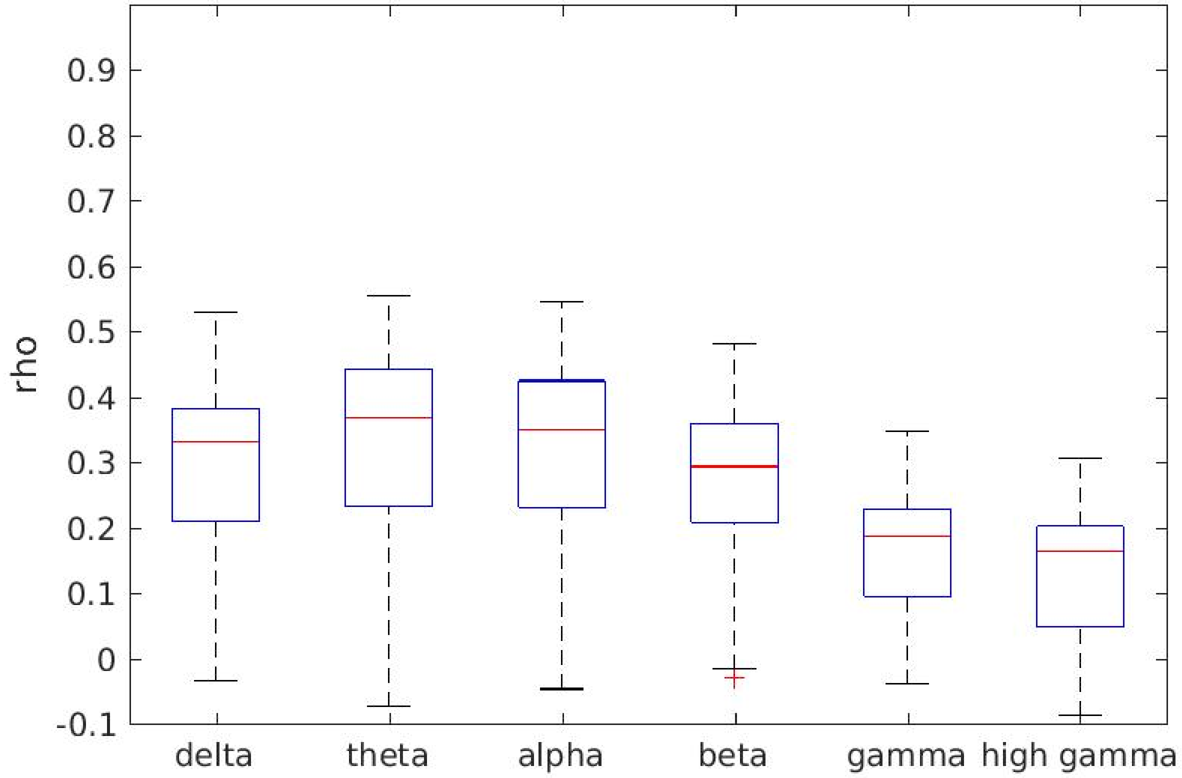

Figure S25. Boxplots of test-retest reliabilities for transfer function in separated frequency ranges.

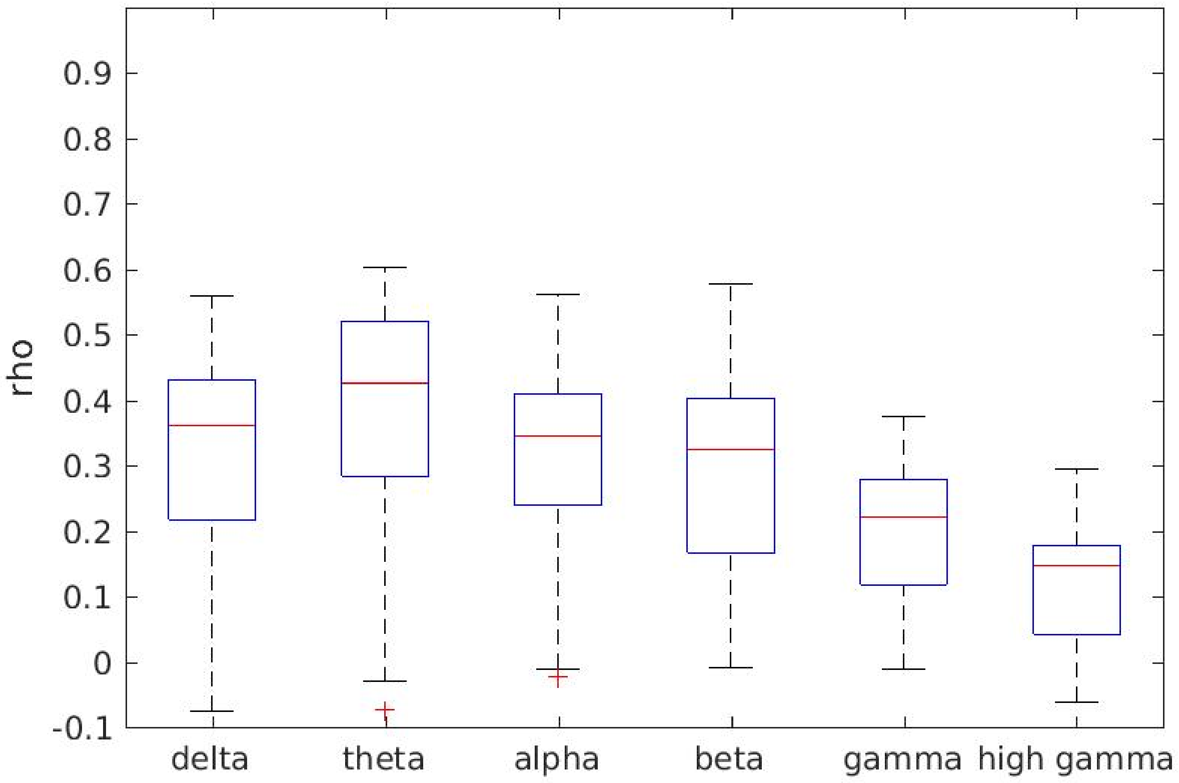

Figure S26. Boxplots of test-retest reliabilities for transfer function polynomial in separated frequency ranges.

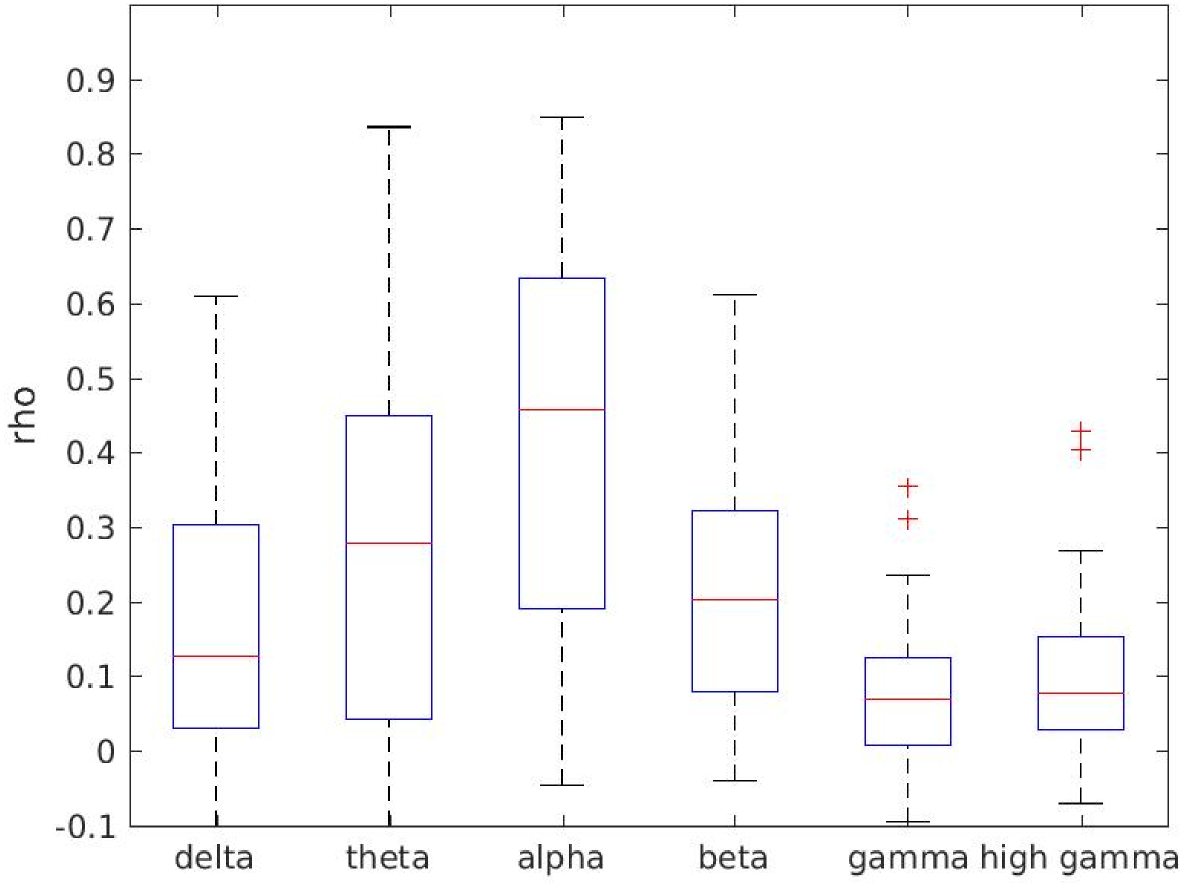

Figure S27. Boxplots of test-retest reliabilities for complex coherence in separated frequency ranges.

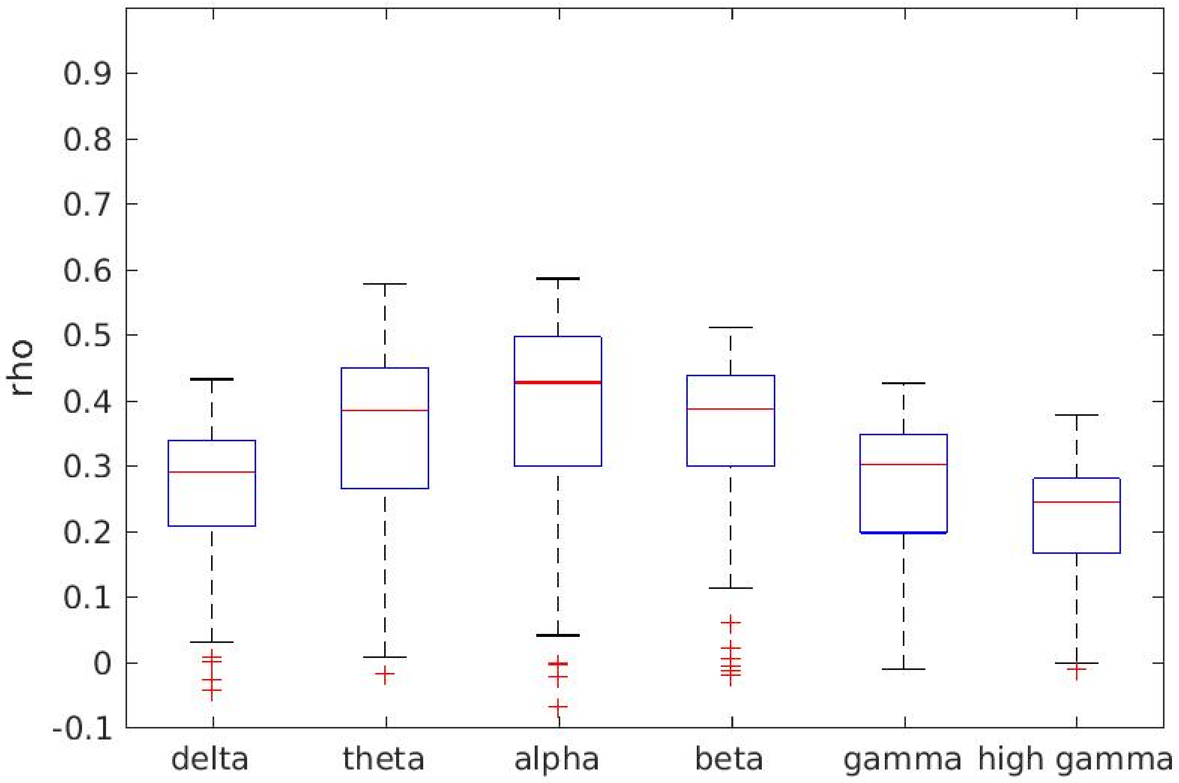

Figure S28. Boxplots of test-retest reliabilities for partial coherence in separated frequency ranges.

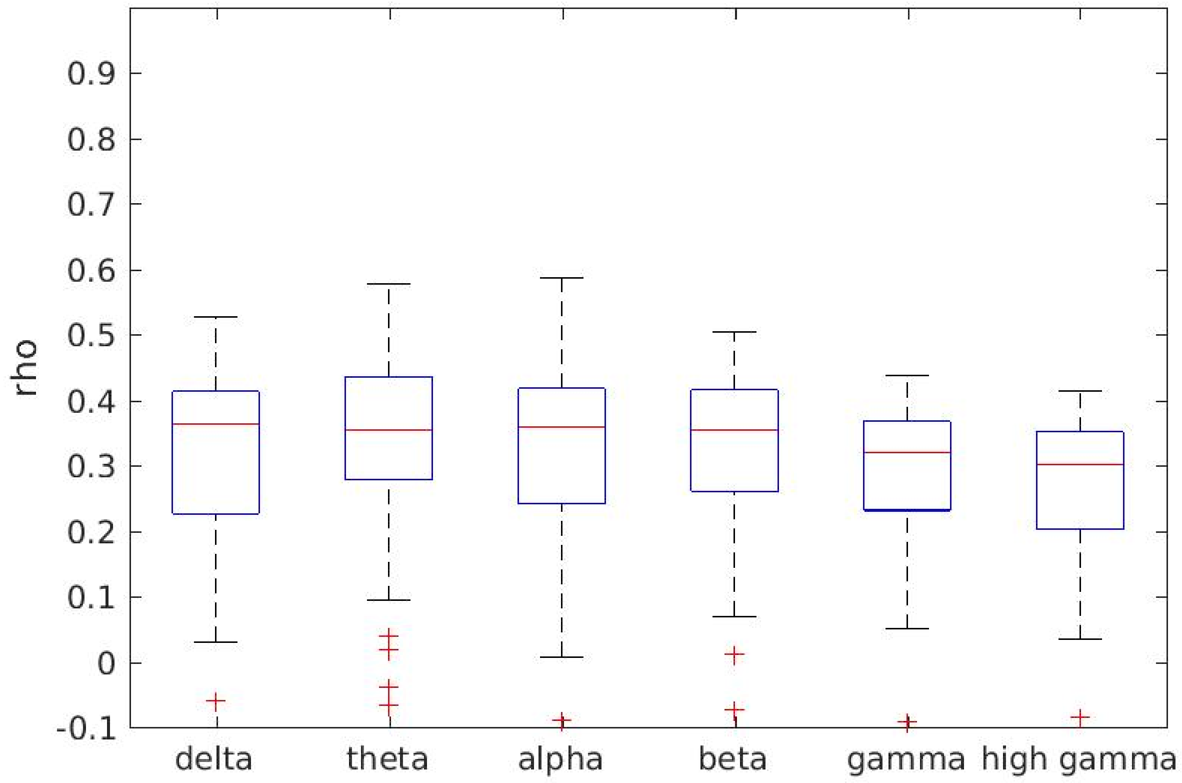

Figure S29. Boxplots of test-retest reliabilities for partial directed coherence in separated frequency ranges.

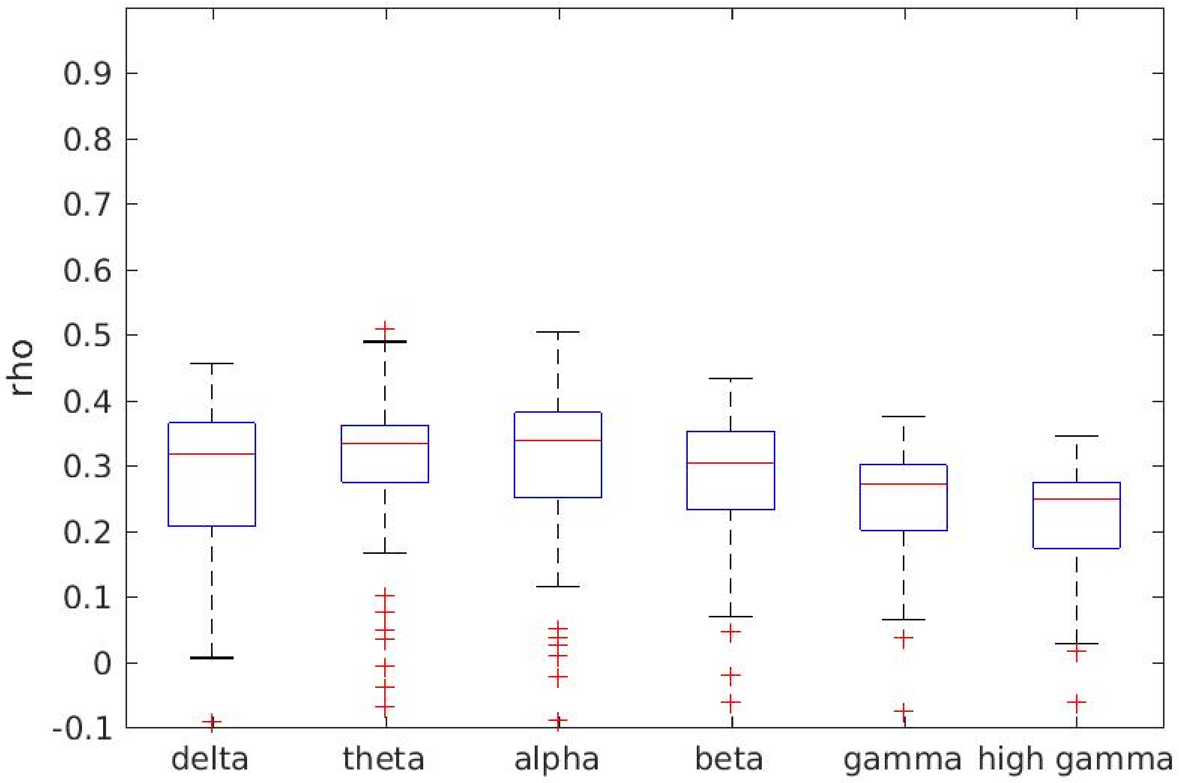

Figure S30. Boxplots of test-retest reliabilities for partial directed coherence factor in separated frequency ranges.

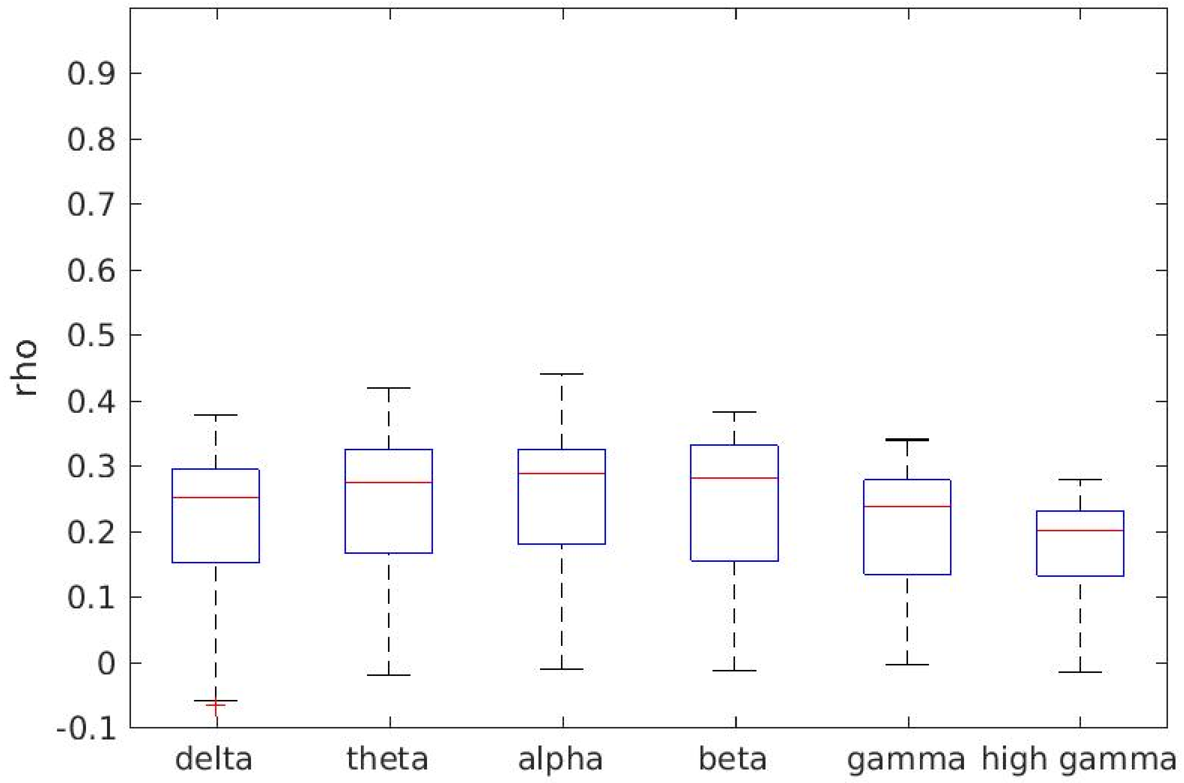

Figure S31. Boxplots of test-retest reliabilities for generalized partial directed coherence in separated frequency ranges.

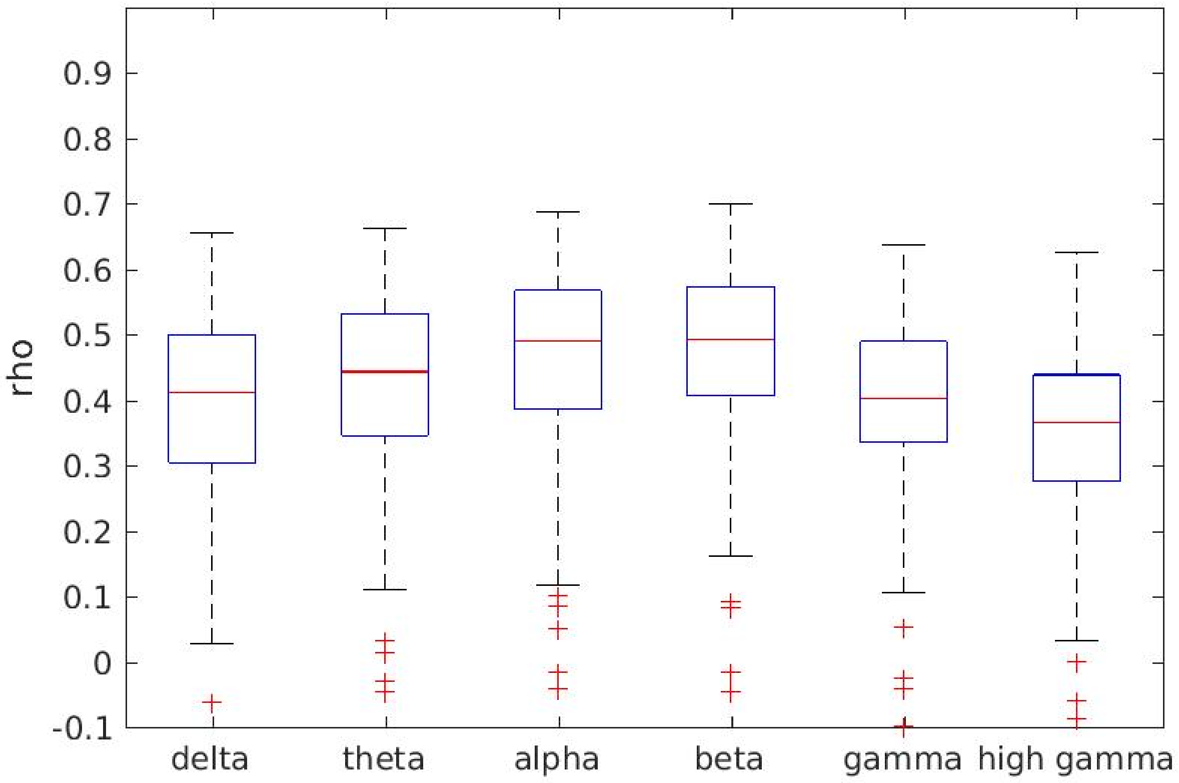

Figure S32. Boxplots of test-retest reliabilities for directed transfer function in separated frequency ranges.

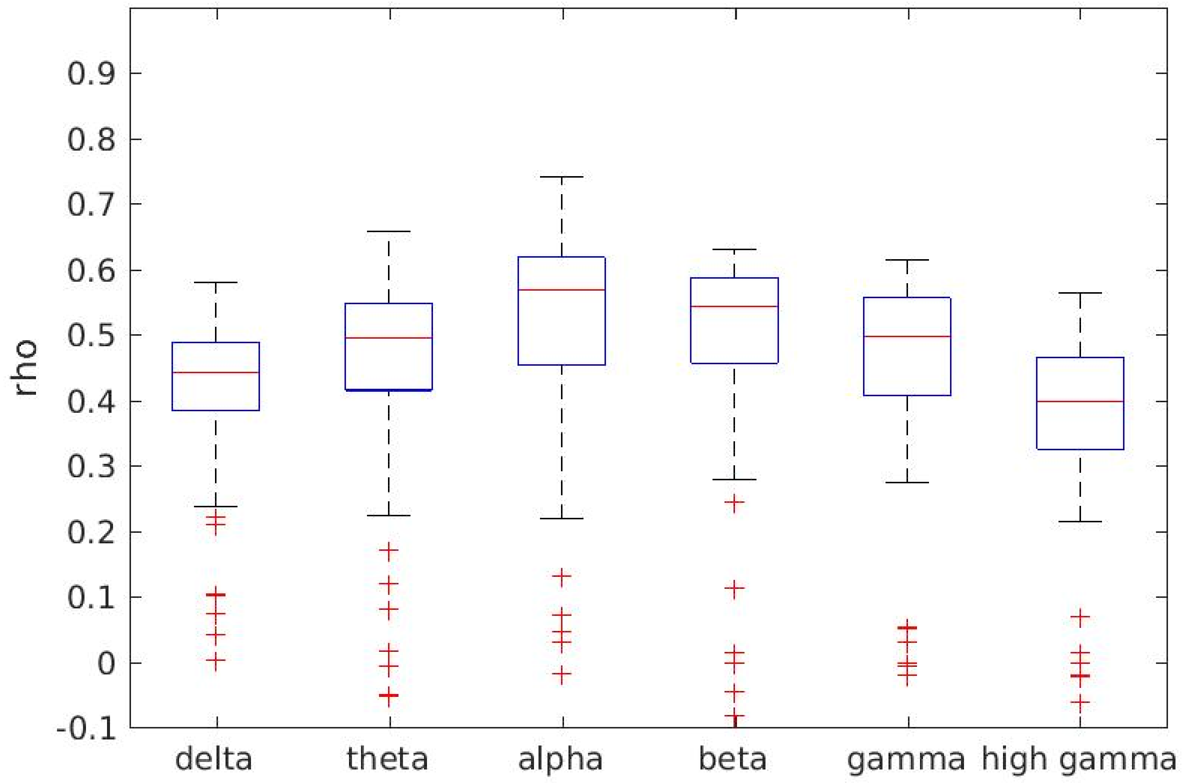

Figure S33. Boxplots of test-retest reliabilities for direct directed transfer function in separated frequency ranges.

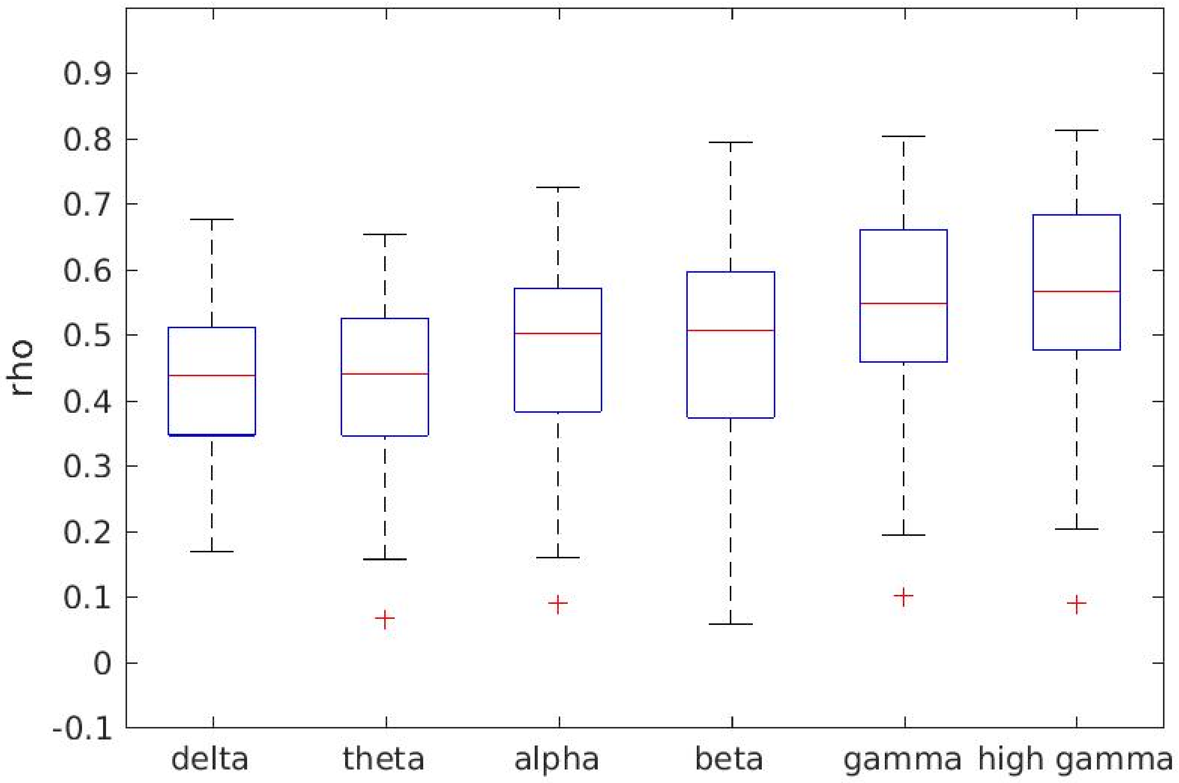

Figure S34. Boxplots of test-retest reliabilities for Geweke's Granger causality in separated frequency ranges.

4 RELATION BETWEEN PATHOLOGY AND DATA LENGTH

We prepared scatter plots representing the course of reliability over the length of the signal used for calculation of measures and coloured the dots according to the neurological populations in Figures S35 to S47. Moreover, we prepared scatter plots representing the relationship between number of artefact-free trials and reliability calculated with artefact-free trials, only, by using dots coloured according to the neurological populations in Figures S48 to S61

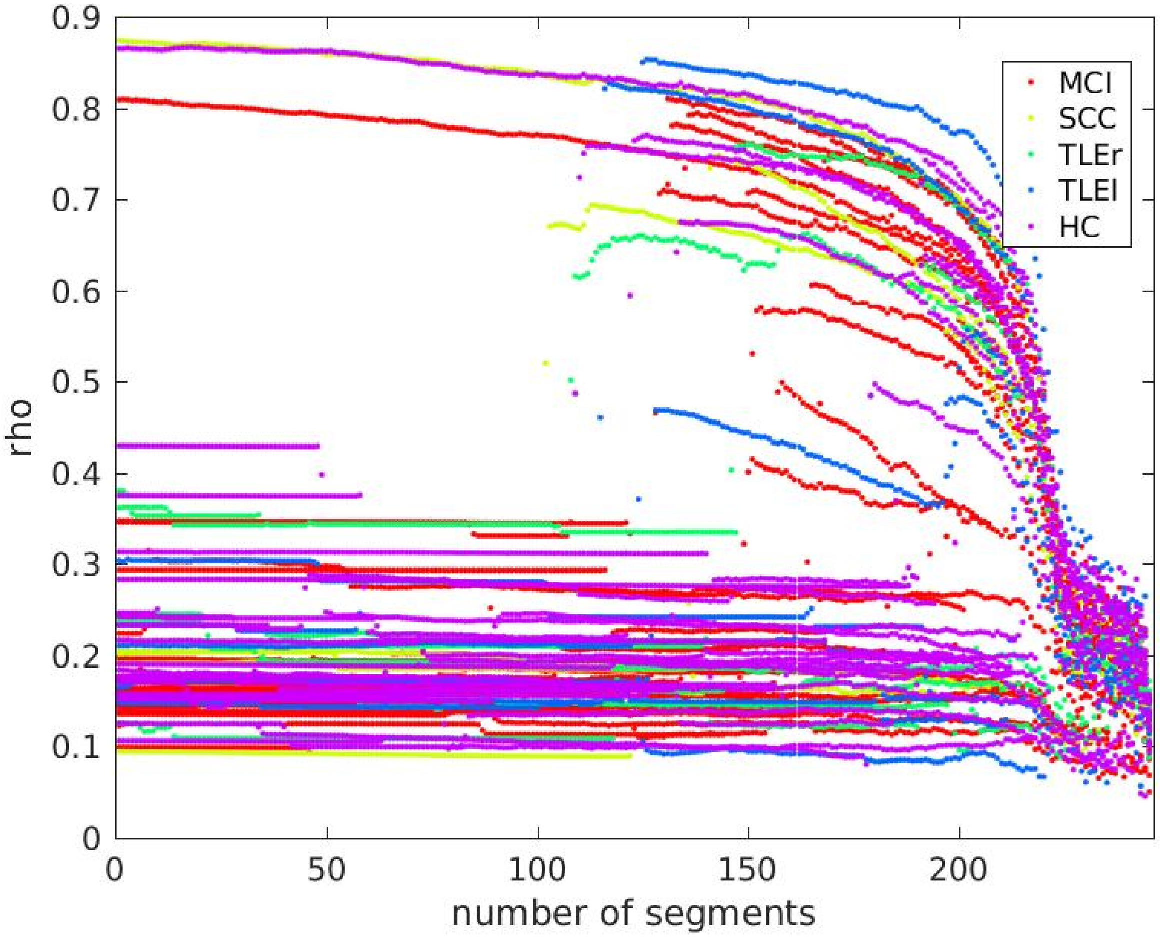

Figure S35. Scatter plot of test-retest reliabilities for spectrum vs. number of excluded segments of size 500ms. The x-axis represents the number of segments that were cut out from the end of the signal. Dots represent values of individual participants. Colors indicate groups MCI= mild cognitive impairment, SCC=subjective cognitive complaints, TLEr= temporal lobe epilepsy with focus on the right hemisphere, TLEl= temporal lobe epilepsy with focus on the left hemisphere, HC=healthy controls.

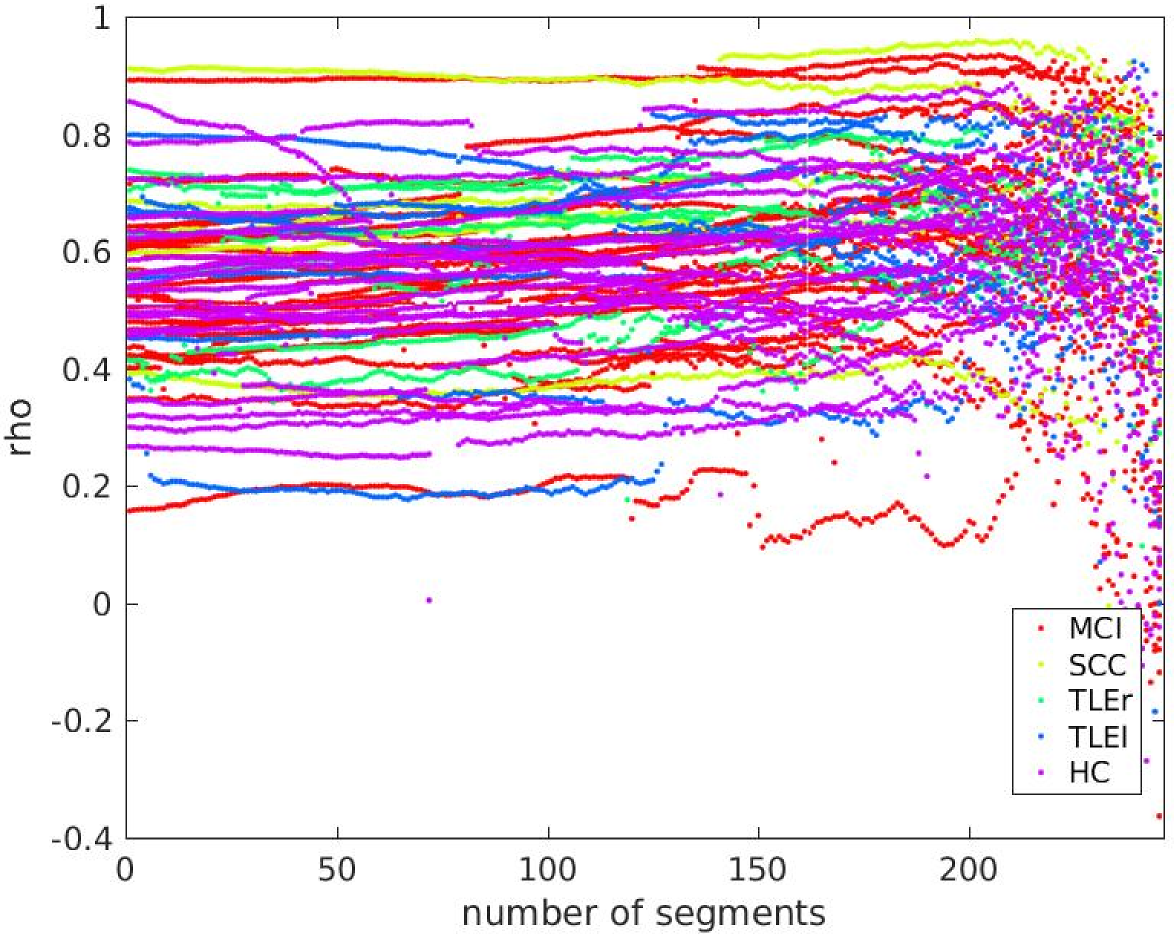

Figure S36. Scatter plot of test-retest reliabilities for direct causality vs. number of excluded segments of size 500ms. The x-axis represents the number of segments that were cut out from the end of the signal. Dots represent values of individual participants. Colors indicate groups MCI= mild cognitive impairment, SCC=subjective cognitive complaints, TLEr= temporal lobe epilepsy with focus on the right hemisphere, TLEl= temporal lobe epilepsy with focus on the left hemisphere, HC=healthy controls.

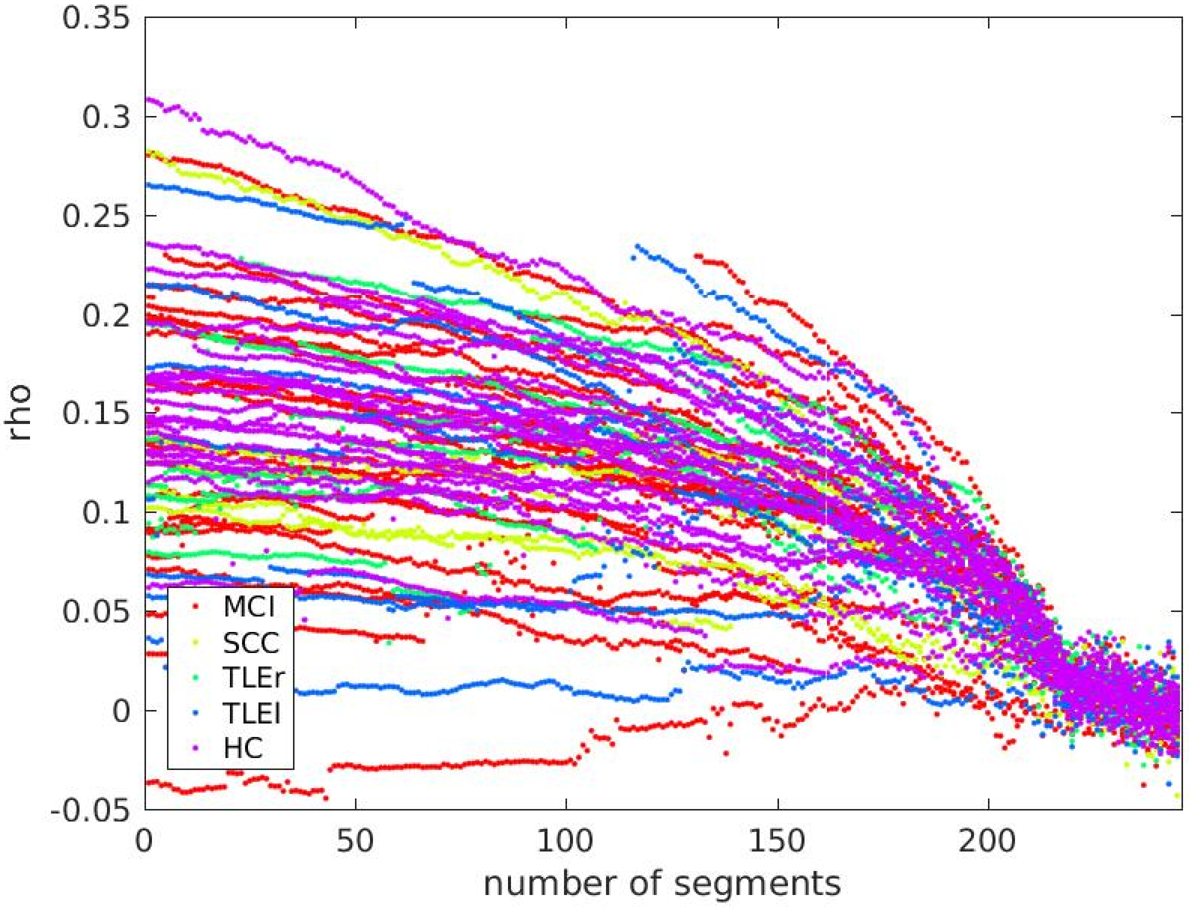

Figure S37. Scatter plot of test-retest reliabilities for transfer function vs. number of excluded segments of size 500ms. The x-axis represents the number of segments that were cut out from the end of the signal. Dots represent values of individual participants. Colors indicate groups MCI= mild cognitive impairment, SCC=subjective cognitive complaints, TLEr= temporal lobe epilepsy with focus on the right hemisphere, TLEl= temporal lobe epilepsy with focus on the left hemisphere, HC=healthy controls.

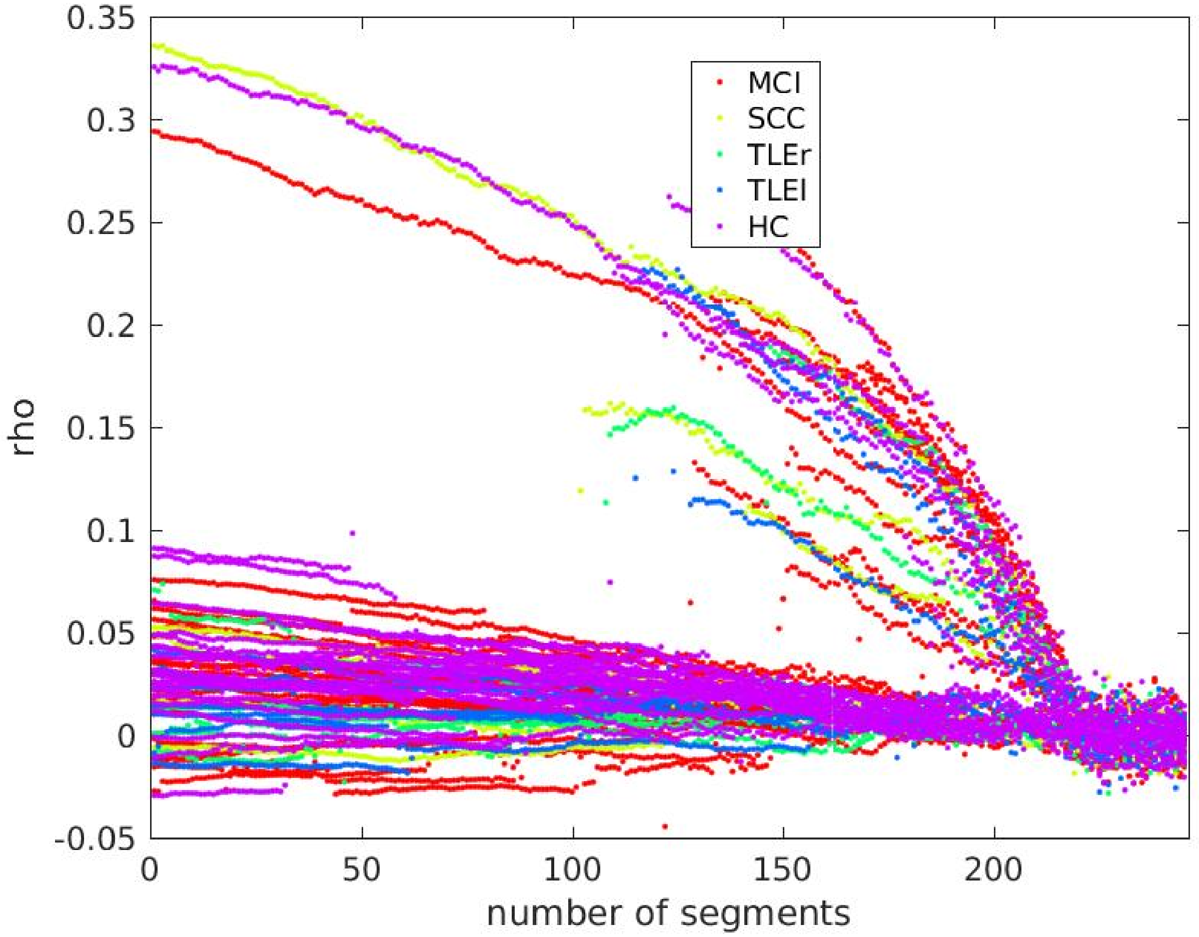

Figure S38. Scatter plot of test-retest reliabilities for transfer function polynomial vs. number of excluded segments of size 500ms. The x-axis represents the number of segments that were cut out from the end of the signal. Dots represent values of individual participants. Colors indicate groups MCI= mild cognitive impairment, SCC=subjective cognitive complaints, TLEr= temporal lobe epilepsy with focus on the right hemisphere, TLEl= temporal lobe epilepsy with focus on the left hemisphere, HC=healthy controls.

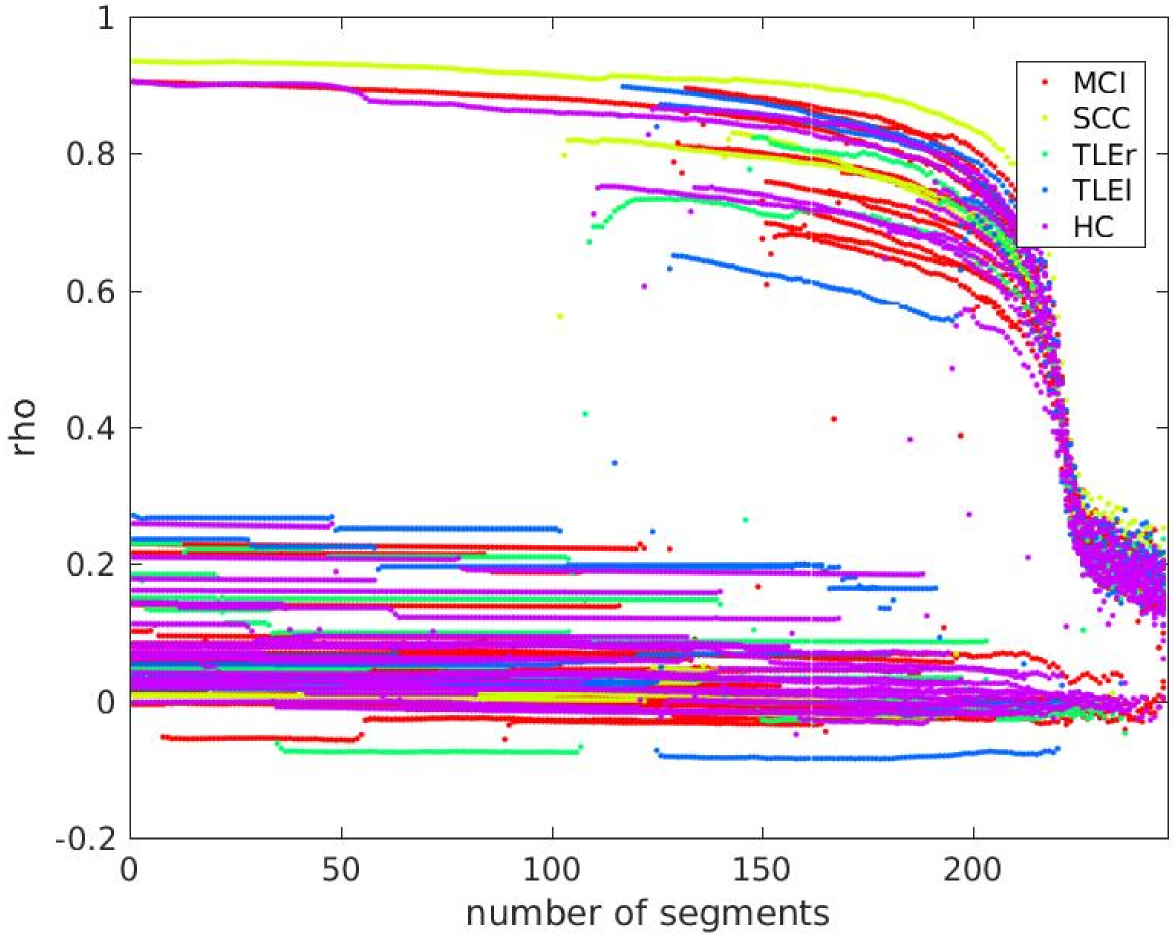

Figure S39. Scatter plot of test-retest reliabilities for real valued coherence vs. number of excluded segments of size 500ms. The x-axis represents the number of segments that were cut out from the end of the signal. Dots represent values of individual participants. Colors indicate groups MCI= mild cognitive impairment, SCC=subjective cognitive complaints, TLEr= temporal lobe epilepsy with focus on the right hemisphere, TLEl= temporal lobe epilepsy with focus on the left hemisphere, HC=healthy controls.

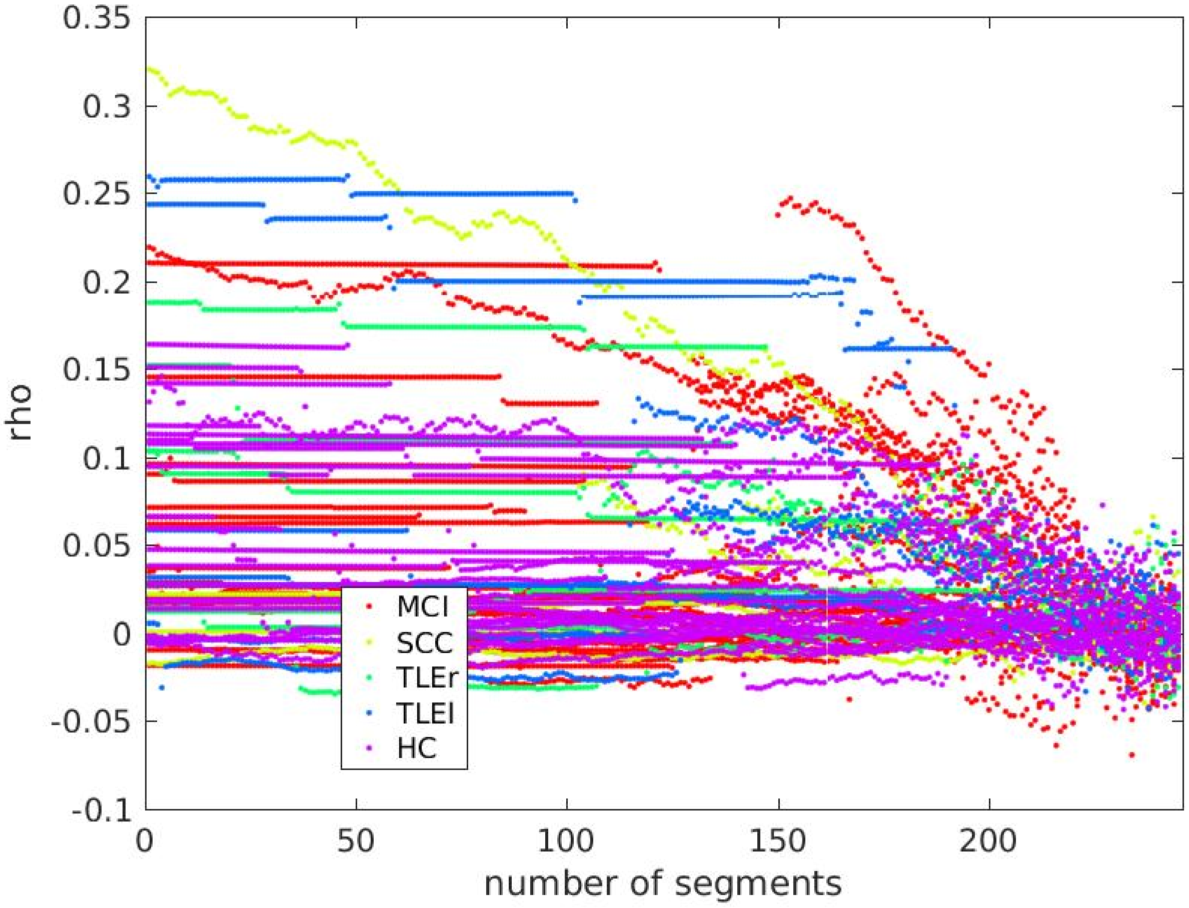

Figure S40. Scatter plot of test-retest reliabilities for complex coherence vs. number of excluded segments of size 500ms. The x-axis represents the number of segments that were cut out from the end of the signal. Dots represent values of individual participants. Colors indicate groups MCI= mild cognitive impairment, SCC=subjective cognitive complaints, TLEr= temporal lobe epilepsy with focus on the right hemisphere, TLEl= temporal lobe epilepsy with focus on the left hemisphere, HC=healthy controls.

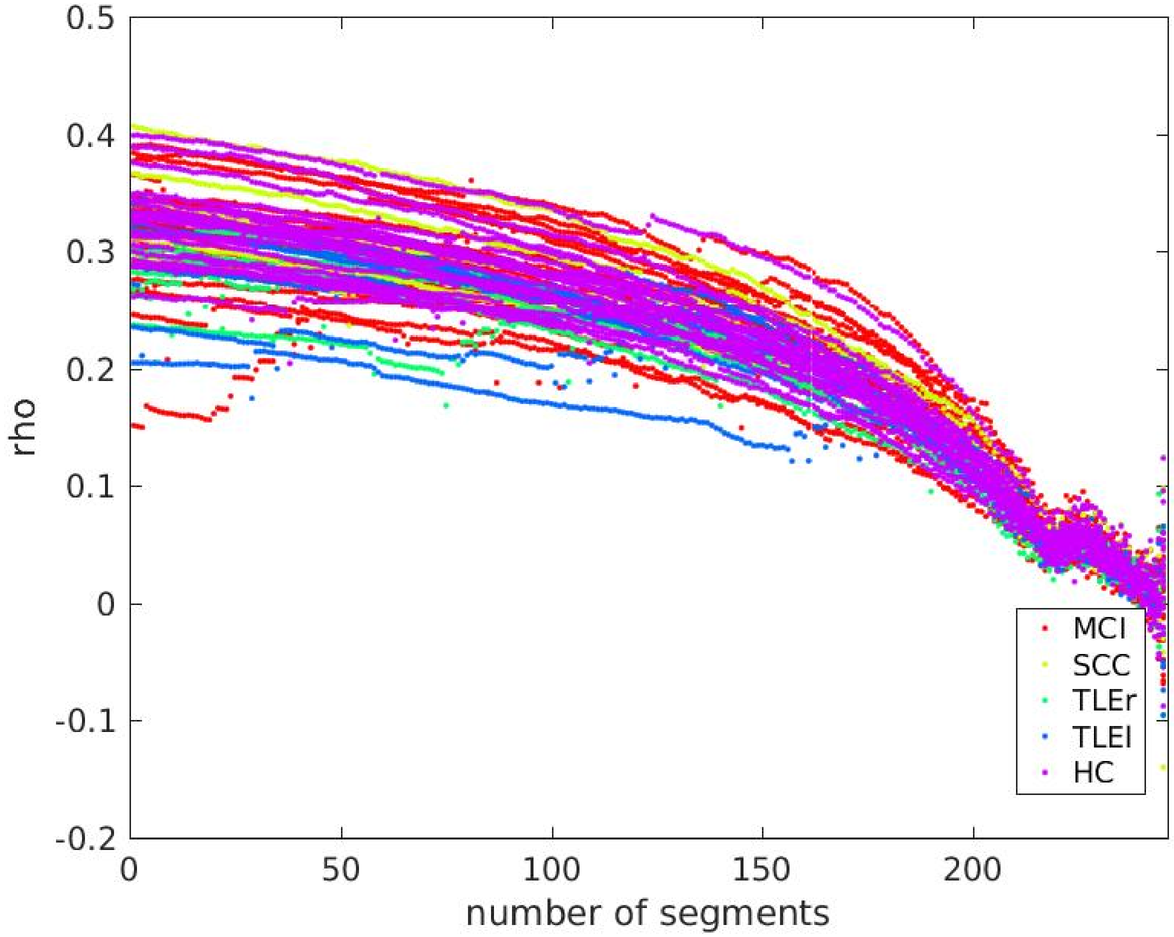

Figure S41. Scatter plot of test-retest reliabilities for partial coherence vs. number of excluded segments of size 500ms. The x-axis represents the number of segments that were cut out from the end of the signal. Dots represent values of individual participants. Colors indicate groups MCI= mild cognitive impairment, SCC=subjective cognitive complaints, TLEr= temporal lobe epilepsy with focus on the right hemisphere, TLEl= temporal lobe epilepsy with focus on the left hemisphere, HC=healthy controls.

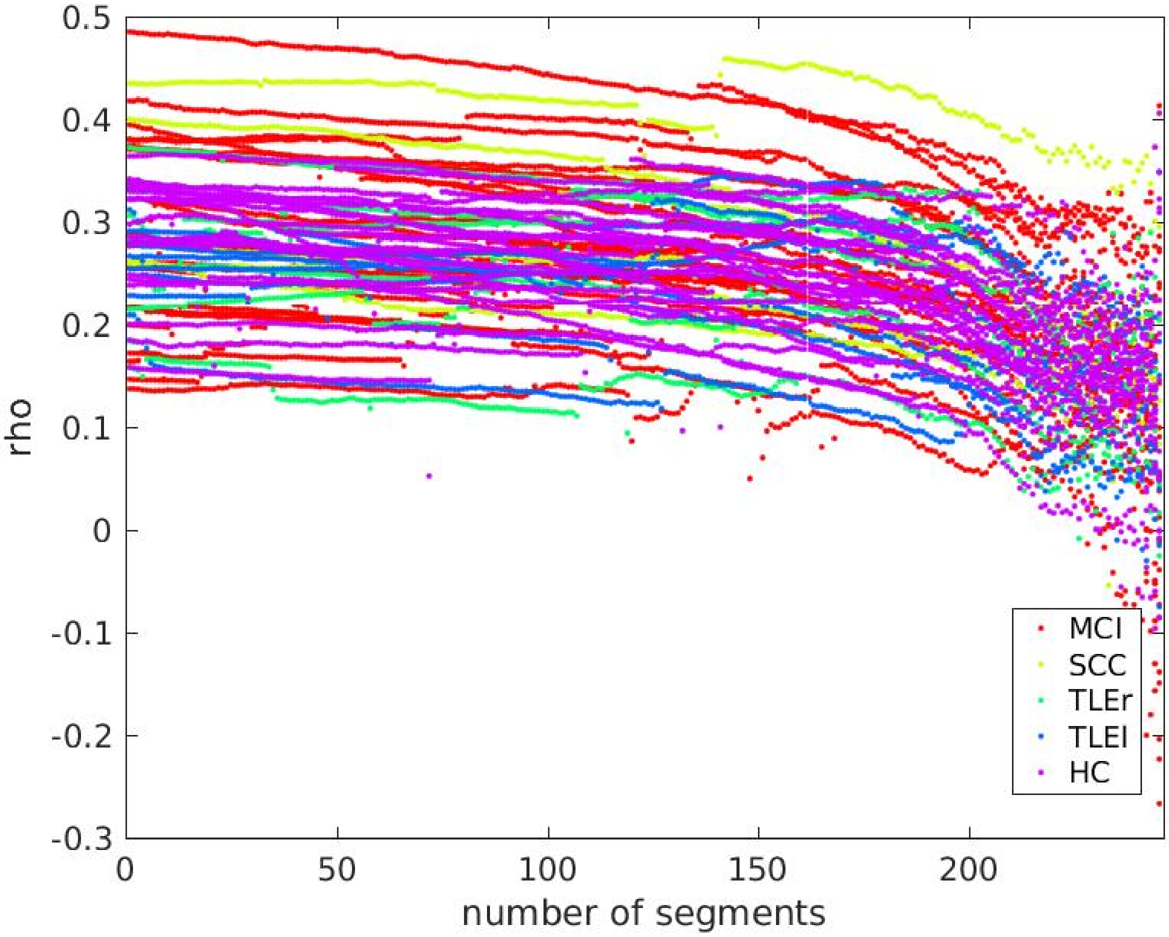

Figure S42. Scatter plot of test-retest reliabilities for partial directed coherence vs. number of excluded segments of size 500ms. The x-axis represents the number of segments that were cut out from the end of the signal. Dots represent values of individual participants. Colors indicate groups MCI= mild cognitive impairment, SCC=subjective cognitive complaints, TLEr= temporal lobe epilepsy with focus on the right hemisphere, TLEl= temporal lobe epilepsy with focus on the left hemisphere, HC=healthy controls.

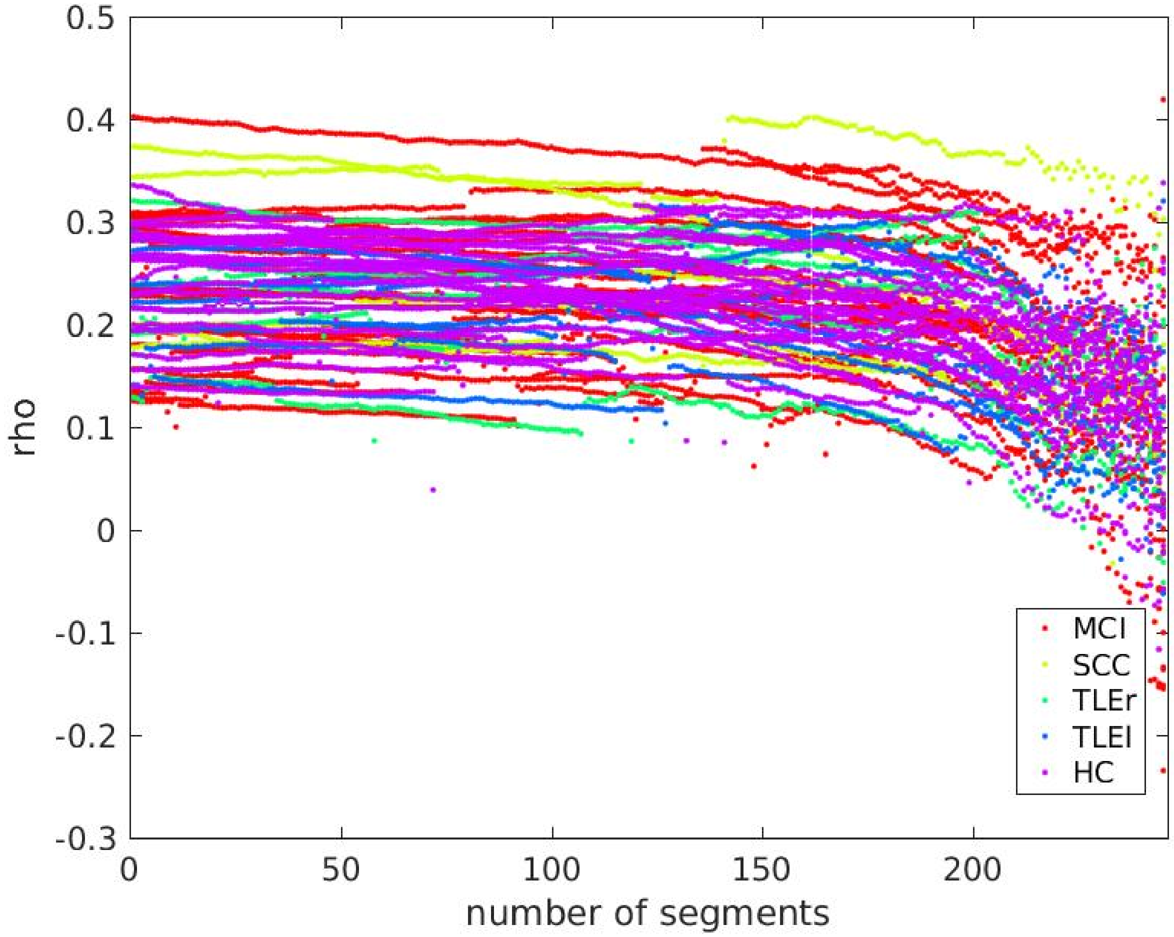

Figure S43. Scatter plot of test-retest reliabilities for partial directed coherence factor vs. number of excluded segments of size 500ms. The x-axis represents the number of segments that were cut out from the end of the signal. Dots represent values of individual participants. Colors indicate groups MCI= mild cognitive impairment, SCC=subjective cognitive complaints, TLEr= temporal lobe epilepsy with focus on the right hemisphere, TLEl= temporal lobe epilepsy with focus on the left hemisphere, HC=healthy controls.

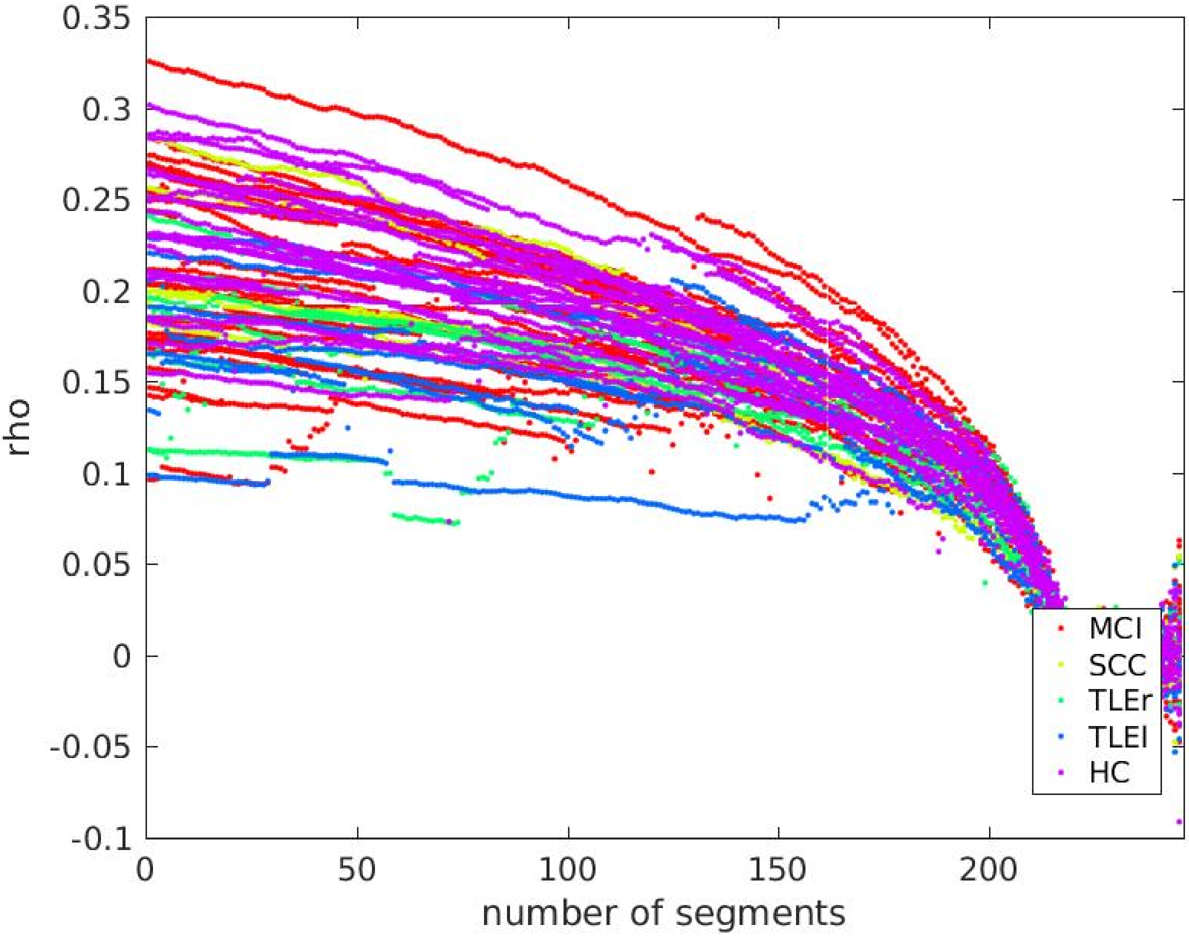

Figure S44. Scatter plot of test-retest reliabilities for generalized partial directed coherence vs. number of excluded segments of size 500ms. The x-axis represents the number of segments that were cut out from the end of the signal. Dots represent values of individual participants. Colors indicate groups MCI= mild cognitive impairment, SCC=subjective cognitive complaints, TLEr= temporal lobe epilepsy with focus on the right hemisphere, TLEl= temporal lobe epilepsy with focus on the left hemisphere, HC=healthy controls.

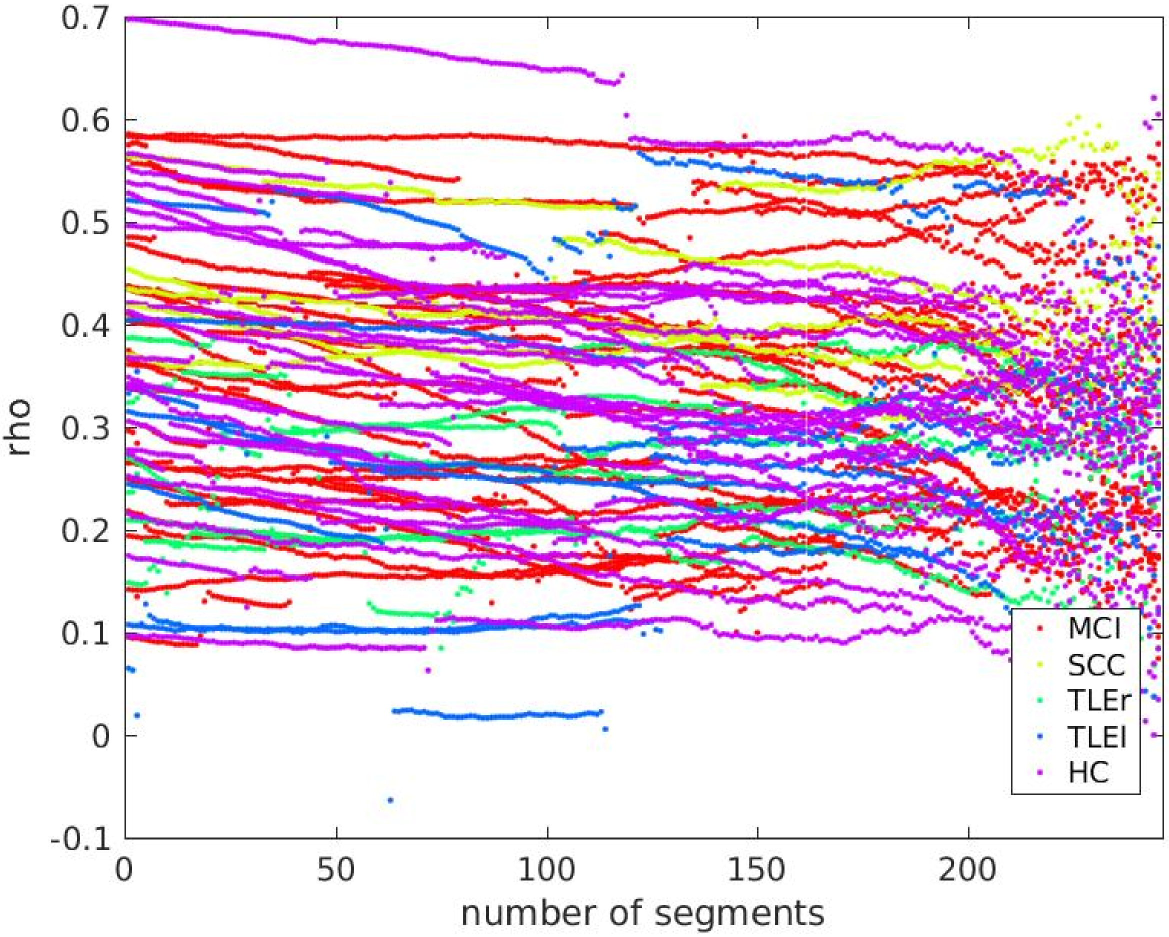

Figure S45. Scatter plot of test-retest reliabilities for directed transfer function vs. number of excluded segments of size 500ms. The x-axis represents the number of segments that were cut out from the end of the signal. Dots represent values of individual participants. Colors indicate groups MCI= mild cognitive impairment, SCC=subjective cognitive complaints, TLEr= temporal lobe epilepsy with focus on the right hemisphere, TLEl= temporal lobe epilepsy with focus on the left hemisphere, HC=healthy controls.

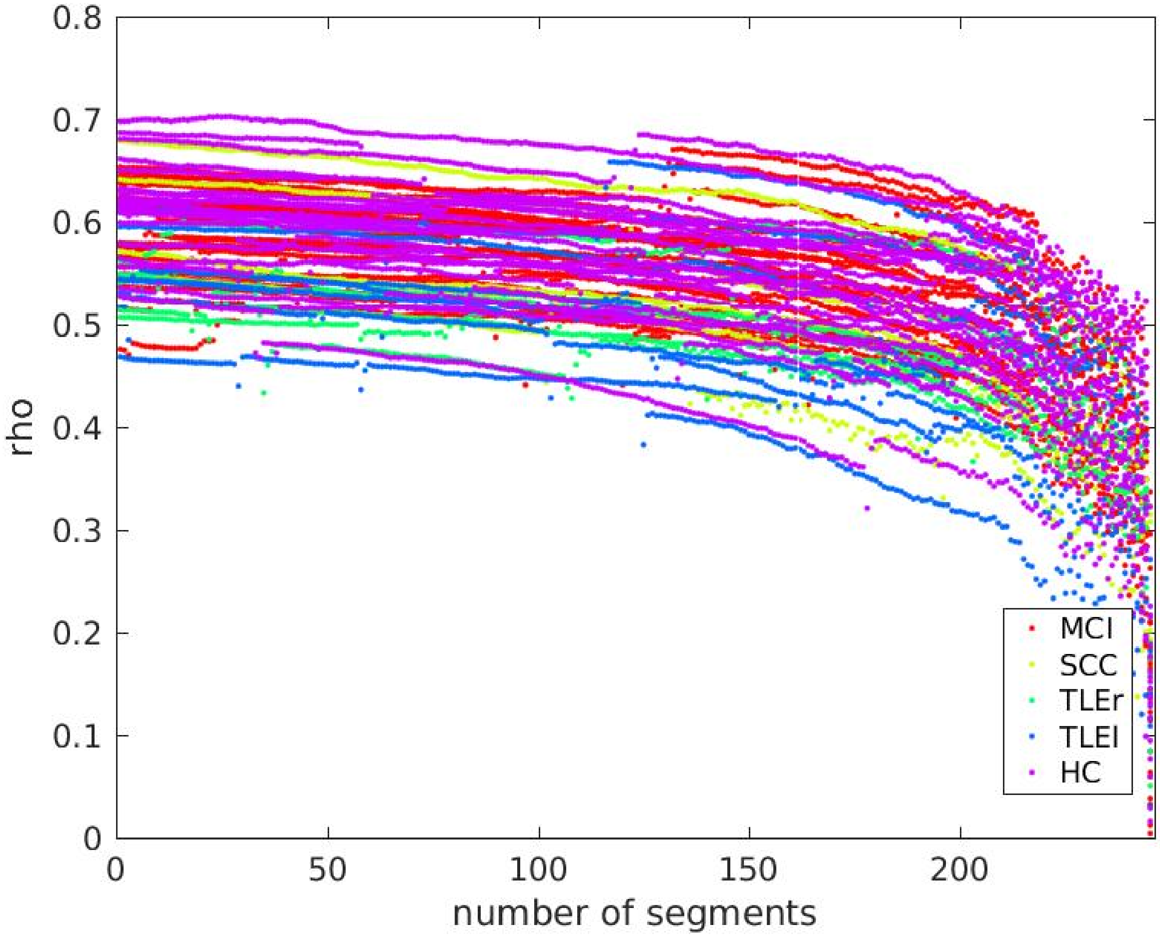

Figure S46. Scatter plot of test-retest reliabilities for direct directed transfer function vs. number of excluded segments of size 500ms. The x-axis represents the number of segments that were cut out from the end of the signal. Dots represent values of individual participants. Colors indicate groups MCI= mild cognitive impairment, SCC=subjective cognitive complaints, TLEr= temporal lobe epilepsy with focus on the right hemisphere, TLEl= temporal lobe epilepsy with focus on the left hemisphere, HC=healthy controls.

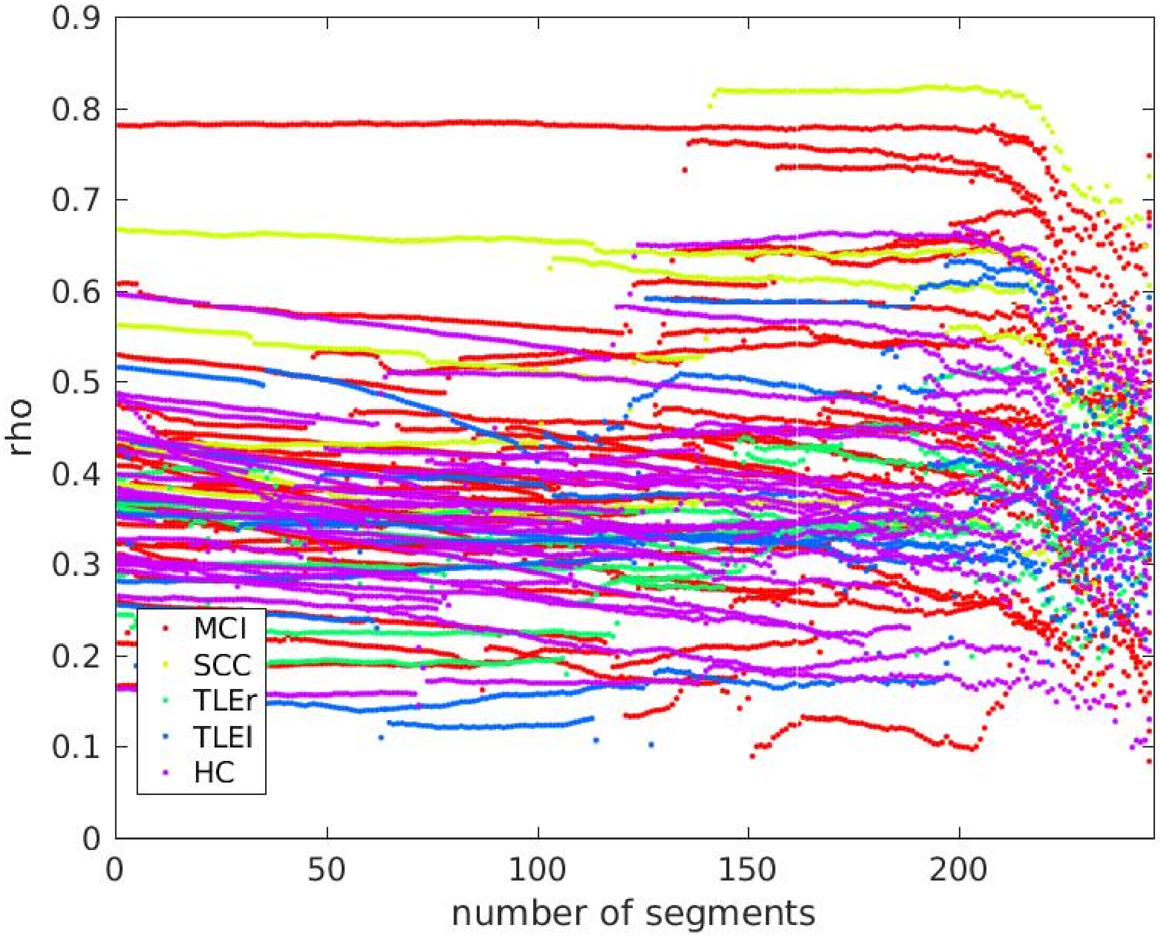

Figure S47. Scatter plot of test-retest reliabilities for Geweke's Granger causality vs. number of excluded segments of size 500ms. The x-axis represents the number of segments that were cut out from the end of the signal. Dots represent values of individual participants. Colors indicate groups MCI= mild cognitive impairment, SCC=subjective cognitive complaints, TLEr= temporal lobe epilepsy with focus on the right hemisphere, TLEl= temporal lobe epilepsy with focus on the left hemisphere, HC=healthy controls.

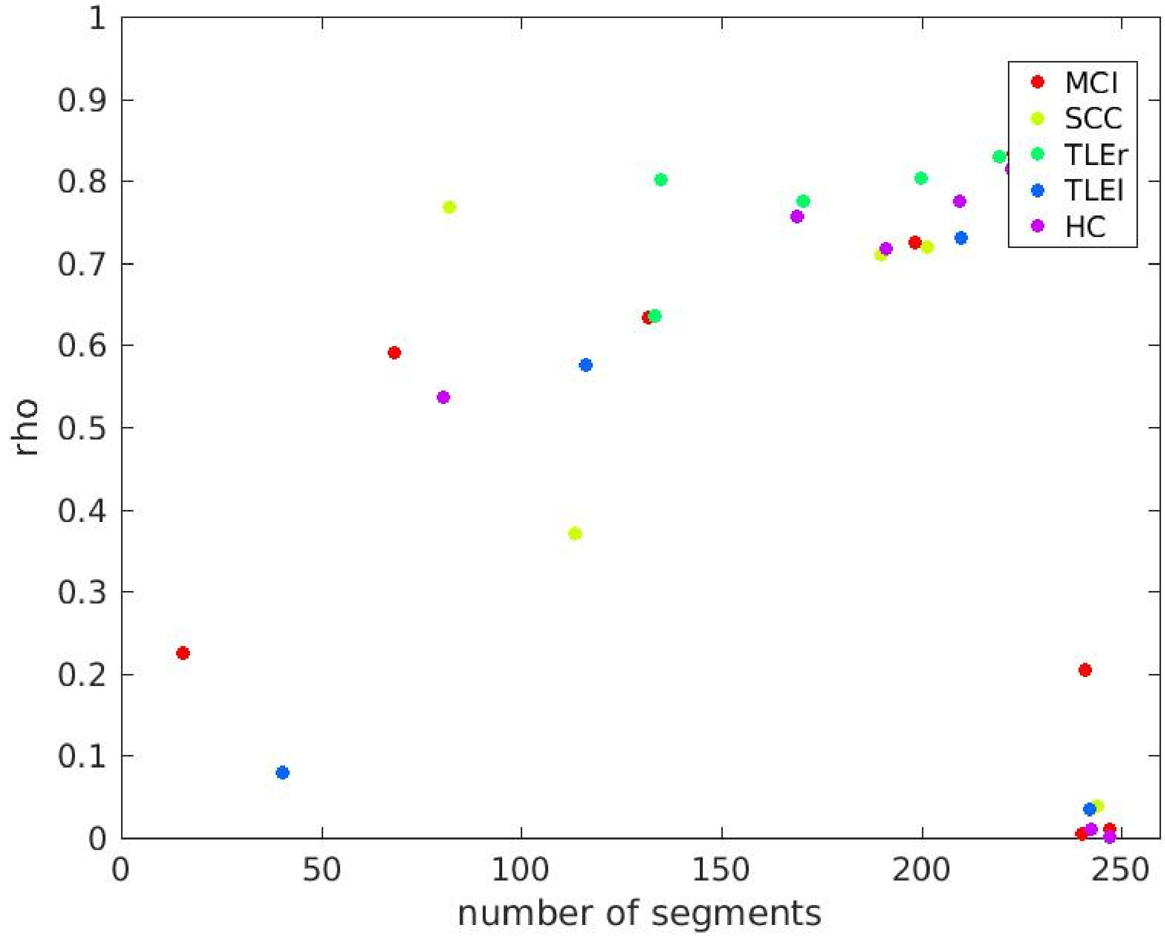

Figure S48. Scatter plot of test-retest reliabilities for spectrum vs. number of artefact-free segments of size 500ms. Calculation of test-retest reliabilities was based on segments without artefacts, only. The x-axis represents the average number of segments across two sessions included in the analysis. Colours indicate groups MCI= mild cognitive impairment, SCC=subjective cognitive complaints, TLEr= temporal lobe epilepsy with focus on the right hemisphere, TLEl= temporal lobe epilepsy with focus on the left hemisphere, HC=healthy controls.

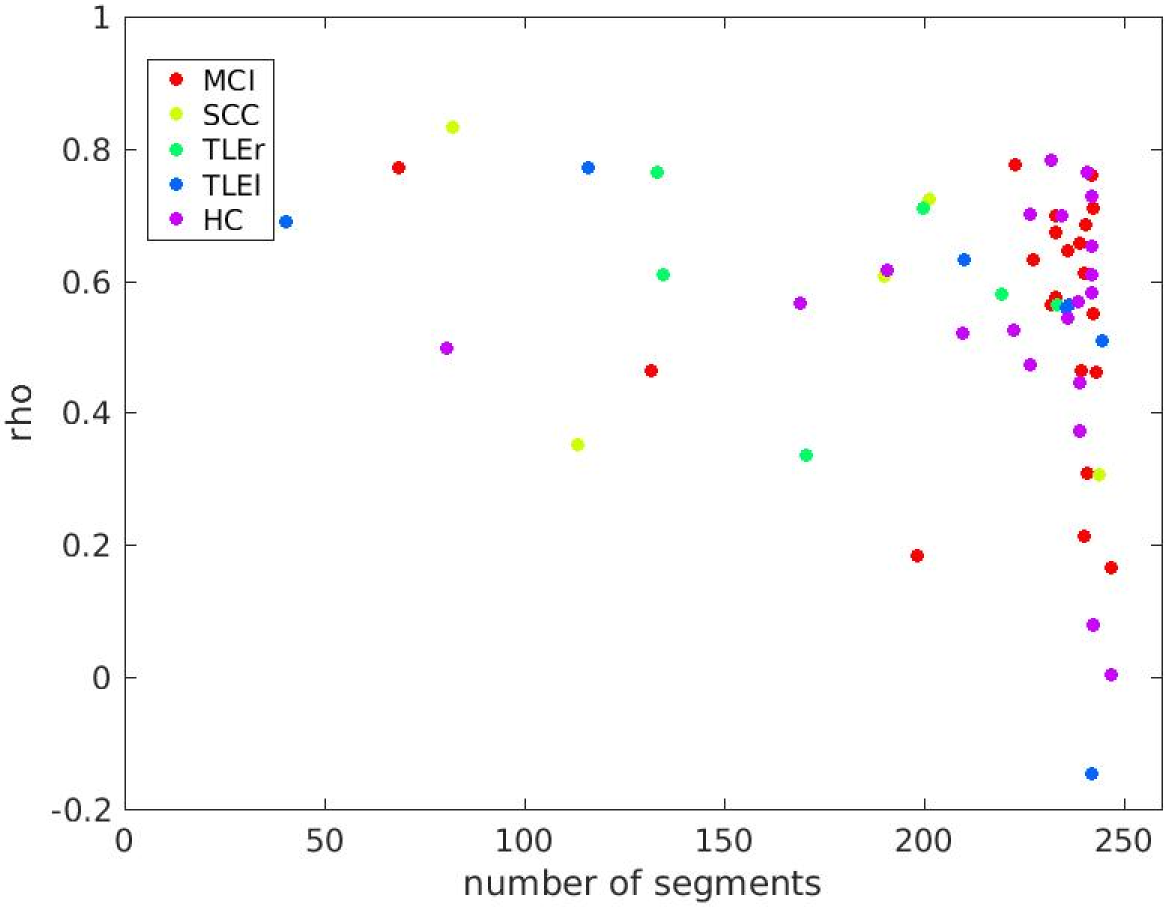

Figure S49. Scatter plot of test-retest reliabilities for direct causality vs. number of artefact-free segments of size 500ms. Calculation of test-retest reliabilities was based on segments without artefacts, only. The x-axis represents the average number of segments across two sessions included in the analysis. Colours indicate groups MCI= mild cognitive impairment, SCC=subjective cognitive complaints, TLEr= temporal lobe epilepsy with focus on the right hemisphere, TLEl= temporal lobe epilepsy with focus on the left hemisphere, HC=healthy controls.

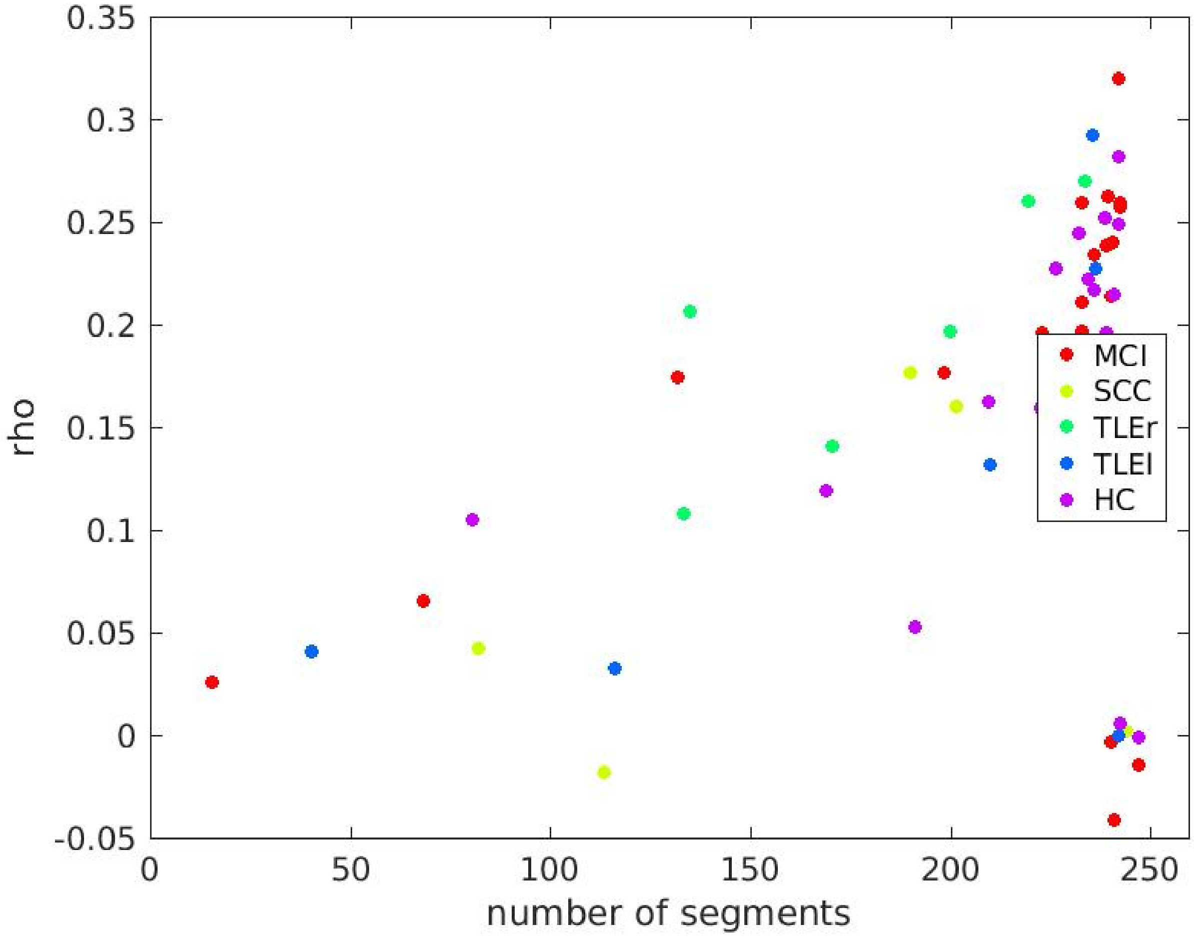

Figure S50. Scatter plot of test-retest reliabilities for transfer function vs. number of artefact-free segments of size 500ms. Calculation of test-retest reliabilities was based on segments without artefacts, only. The x-axis represents the average number of segments across two sessions included in the analysis. Colours indicate groups MCI= mild cognitive impairment, SCC=subjective cognitive complaints, TLEr= temporal lobe epilepsy with focus on the right hemisphere, TLEl= temporal lobe epilepsy with focus on the left hemisphere, HC=healthy controls.

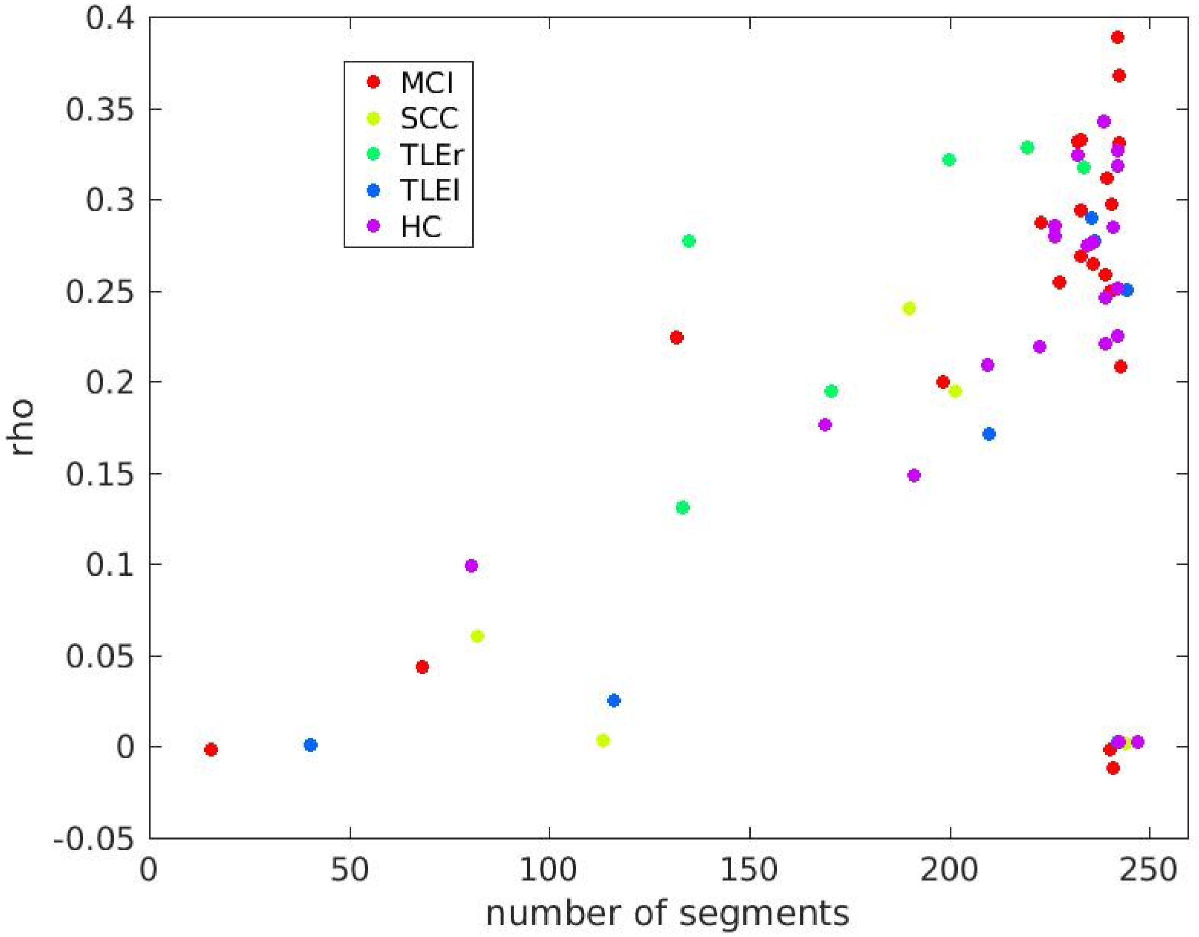

Figure S51. Scatter plot of test-retest reliabilities for transfer function polynomial vs. number of artefact-free segments of size 500ms. Calculation of test-retest reliabilities was based on segments without artefacts, only. The x-axis represents the average number of segments across two sessions included in the analysis. Colours indicate groups MCI= mild cognitive impairment, SCC=subjective cognitive complaints, TLEr= temporal lobe epilepsy with focus on the right hemisphere, TLEl= temporal lobe epilepsy with focus on the left hemisphere, HC=healthy controls.

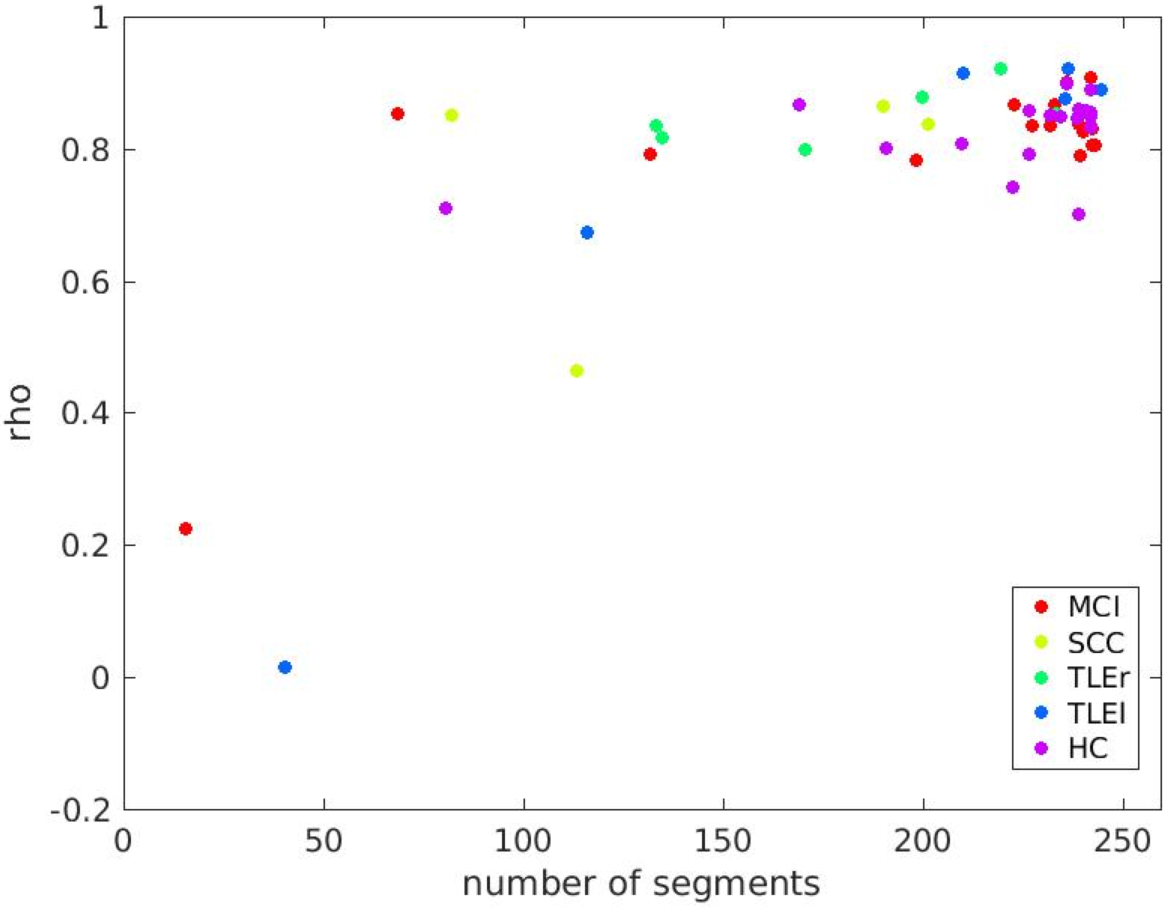

Figure S52. Scatter plot of test-retest reliabilities for real valued coherence vs. number of artefact-free segments of size 500ms. Calculation of test-retest reliabilities was based on segments without artefacts, only. The x-axis represents the average number of segments across two sessions included in the analysis. Colours indicate groups MCI= mild cognitive impairment, SCC=subjective cognitive complaints, TLEr= temporal lobe epilepsy with focus on the right hemisphere, TLEl= temporal lobe epilepsy with focus on the left hemisphere, HC=healthy controls.

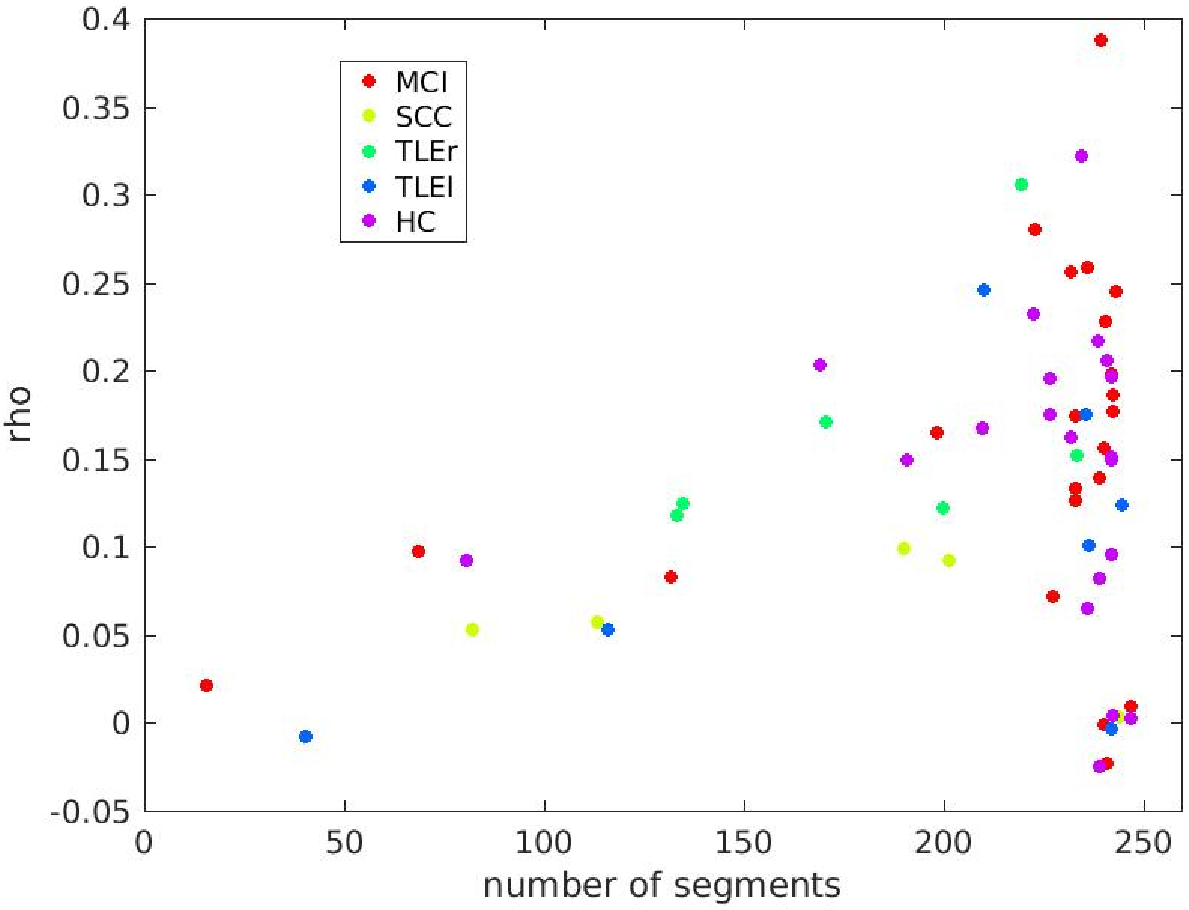

Figure S53. Scatter plot of test-retest reliabilities for complex coherence vs. number of artefact-free segments of size 500ms. Calculation of test-retest reliabilities was based on segments without artefacts, only. The x-axis represents the average number of segments across two sessions included in the analysis. Colours indicate groups MCI= mild cognitive impairment, SCC=subjective cognitive complaints, TLEr= temporal lobe epilepsy with focus on the right hemisphere, TLEl= temporal lobe epilepsy with focus on the left hemisphere, HC=healthy controls.

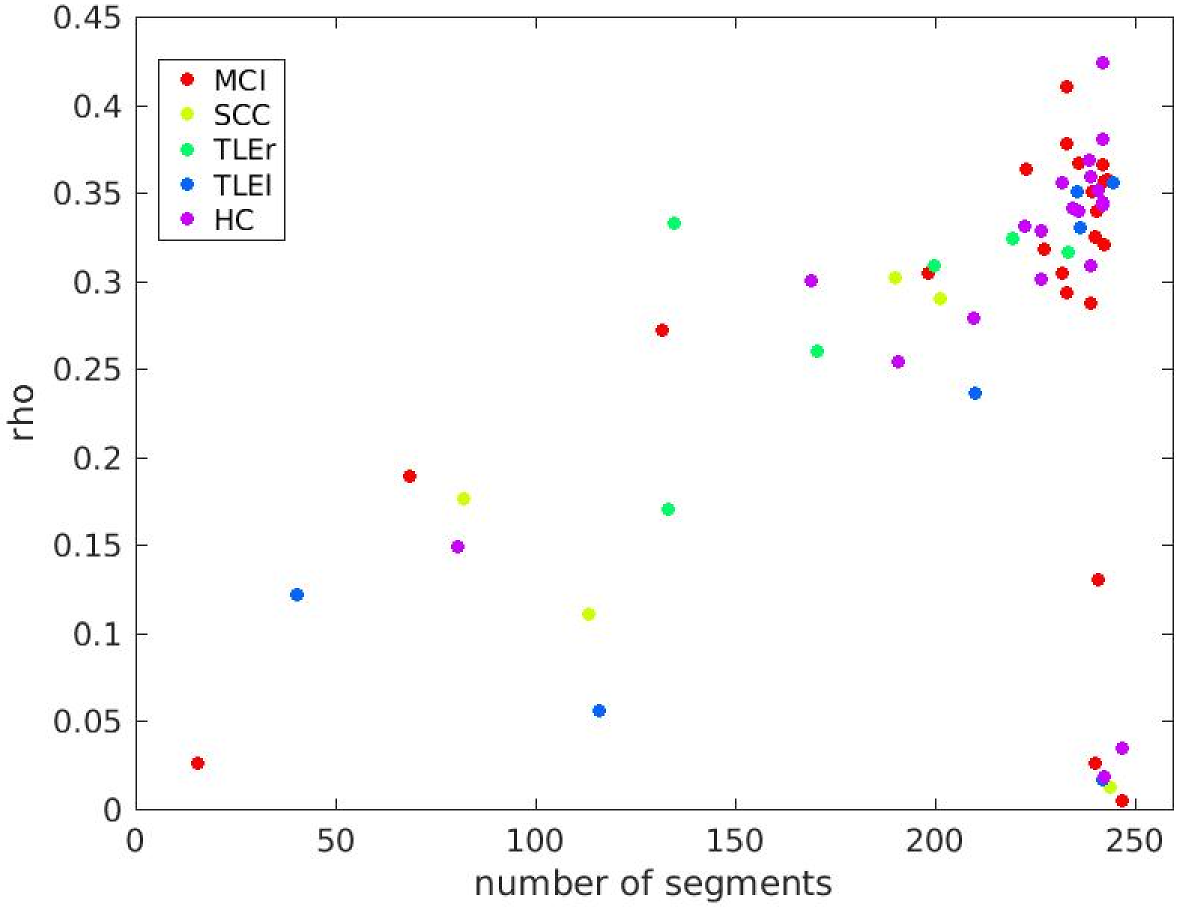

Figure S54. Scatter plot of test-retest reliabilities for partial coherence vs. number of artefact-free segments of size 500ms. Calculation of test-retest reliabilities was based on segments without artefacts, only. The x-axis represents the average number of segments across two sessions included in the analysis. Colours indicate groups MCI= mild cognitive impairment, SCC=subjective cognitive complaints, TLEr= temporal lobe epilepsy with focus on the right hemisphere, TLEl= temporal lobe epilepsy with focus on the left hemisphere, HC=healthy controls.

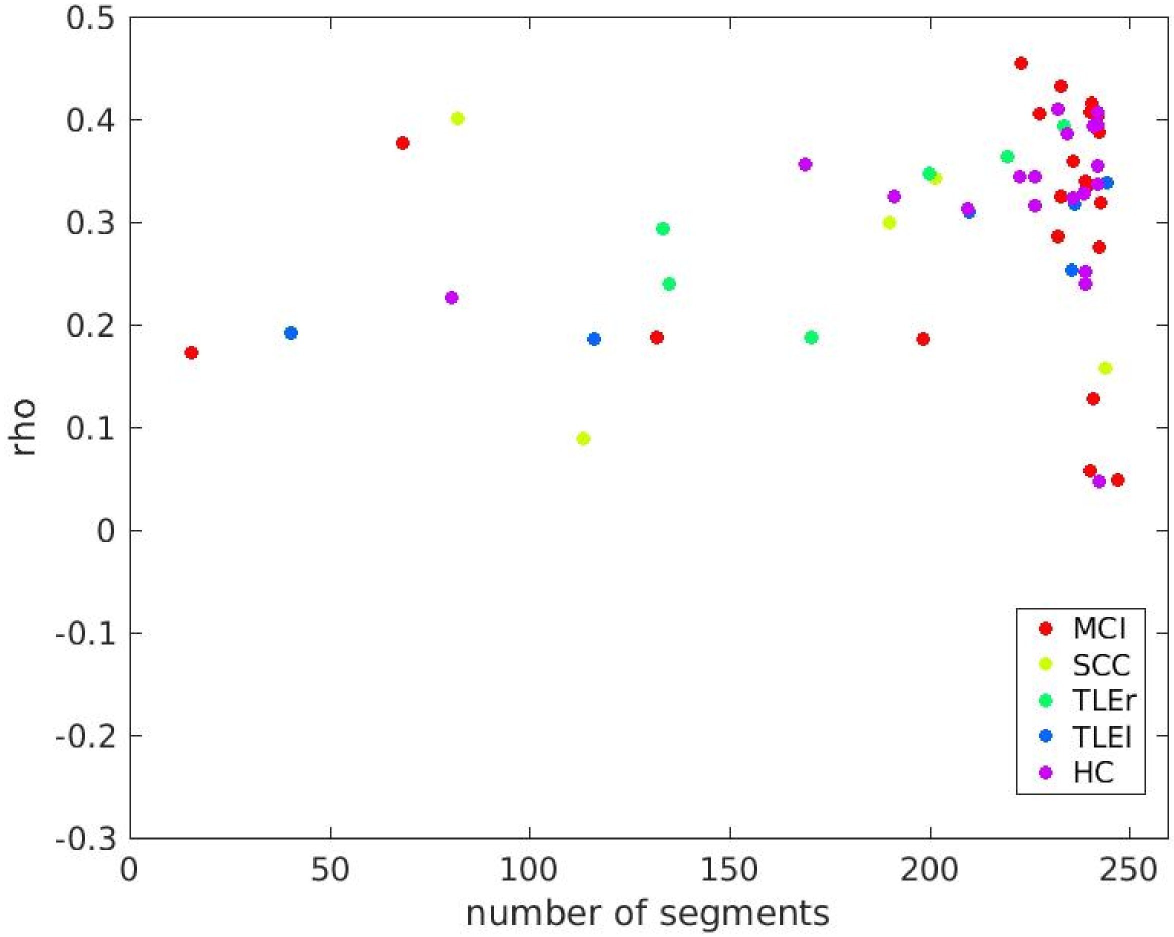

Figure S55. Scatter plot of test-retest reliabilities for partial directed coherence vs. number of artefact-free segments of size 500ms. Calculation of test-retest reliabilities was based on segments without artefacts, only. The x-axis represents the average number of segments across two sessions included in the analysis. Colours indicate groups MCI= mild cognitive impairment, SCC=subjective cognitive complaints, TLEr= temporal lobe epilepsy with focus on the right hemisphere, TLEl= temporal lobe epilepsy with focus on the left hemisphere, HC=healthy controls.

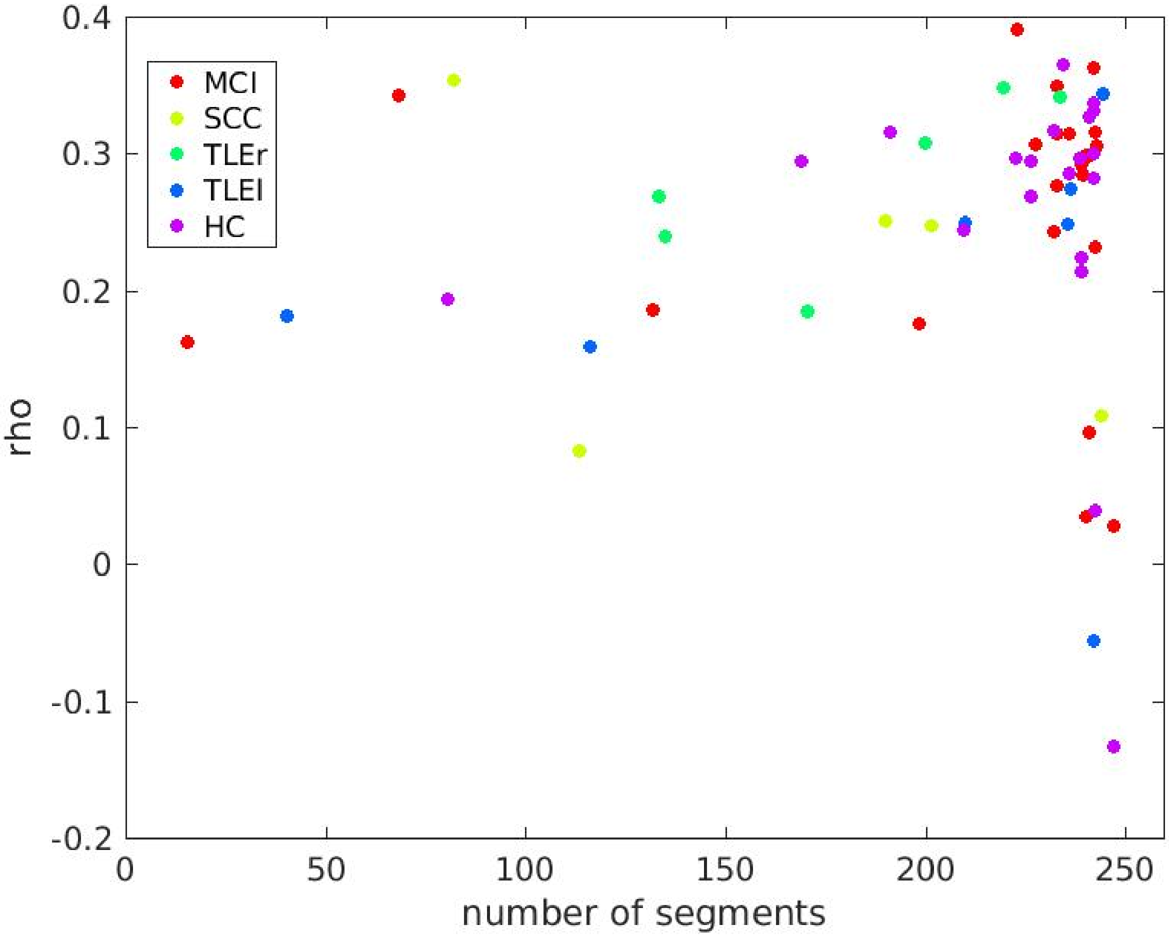

Figure S56. Scatter plot of test-retest reliabilities for partial directed coherence factor vs. number of artefact-free segments of size 500ms. Calculation of test-retest reliabilities was based on segments without artefacts, only. The x-axis represents the average number of segments across two sessions included in the analysis. Colours indicate groups MCI= mild cognitive impairment, SCC=subjective cognitive complaints, TLEr= temporal lobe epilepsy with focus on the right hemisphere, TLEl= temporal lobe epilepsy with focus on the left hemisphere, HC=healthy controls.

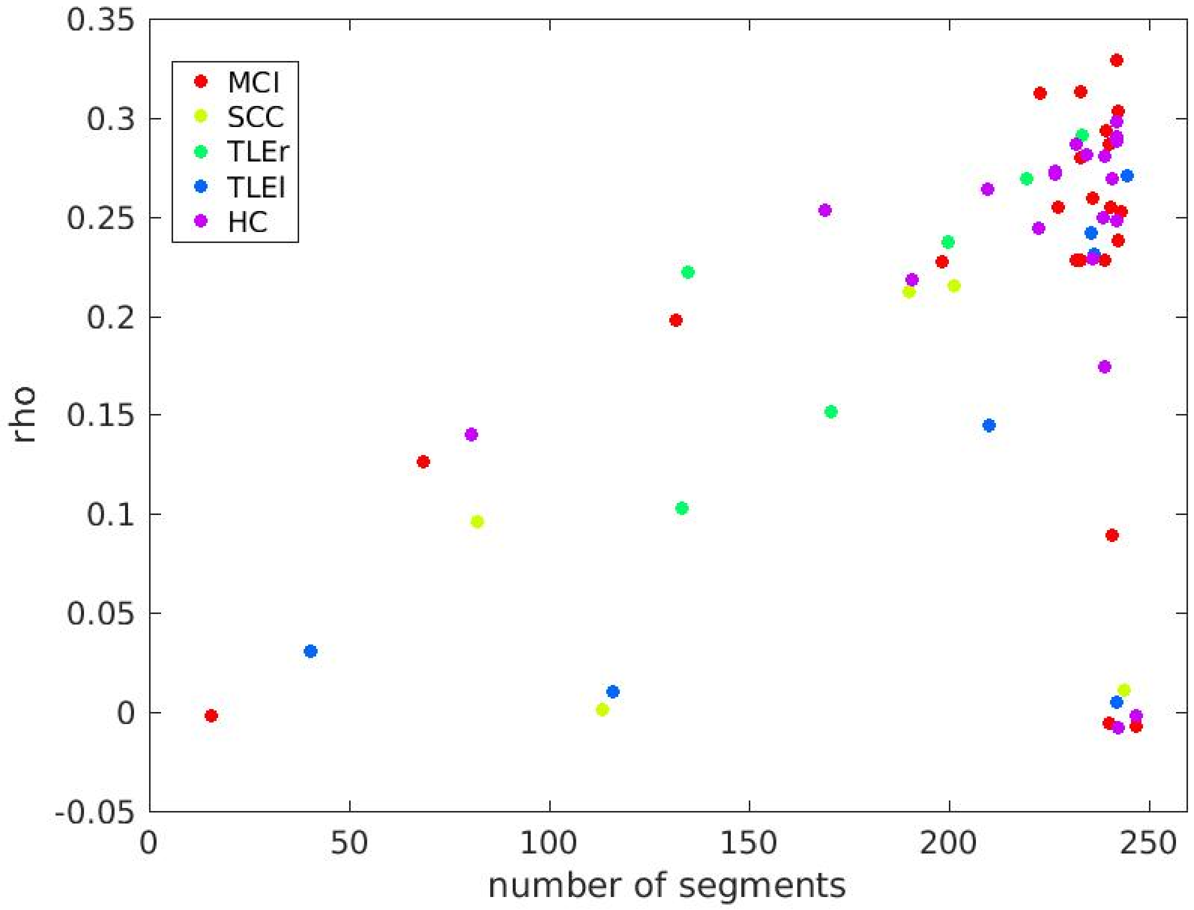

Figure S57. Scatter plot of test-retest reliabilities for generalized partial directed coherence vs. number of artefact-free segments of size 500ms. Calculation of test-retest reliabilities was based on segments without artefacts, only. The x-axis represents the average number of segments across two sessions included in the analysis. Colours indicate groups MCI= mild cognitive impairment, SCC=subjective cognitive complaints, TLEr= temporal lobe epilepsy with focus on the right hemisphere, TLEl= temporal lobe epilepsy with focus on the left hemisphere, HC=healthy controls.

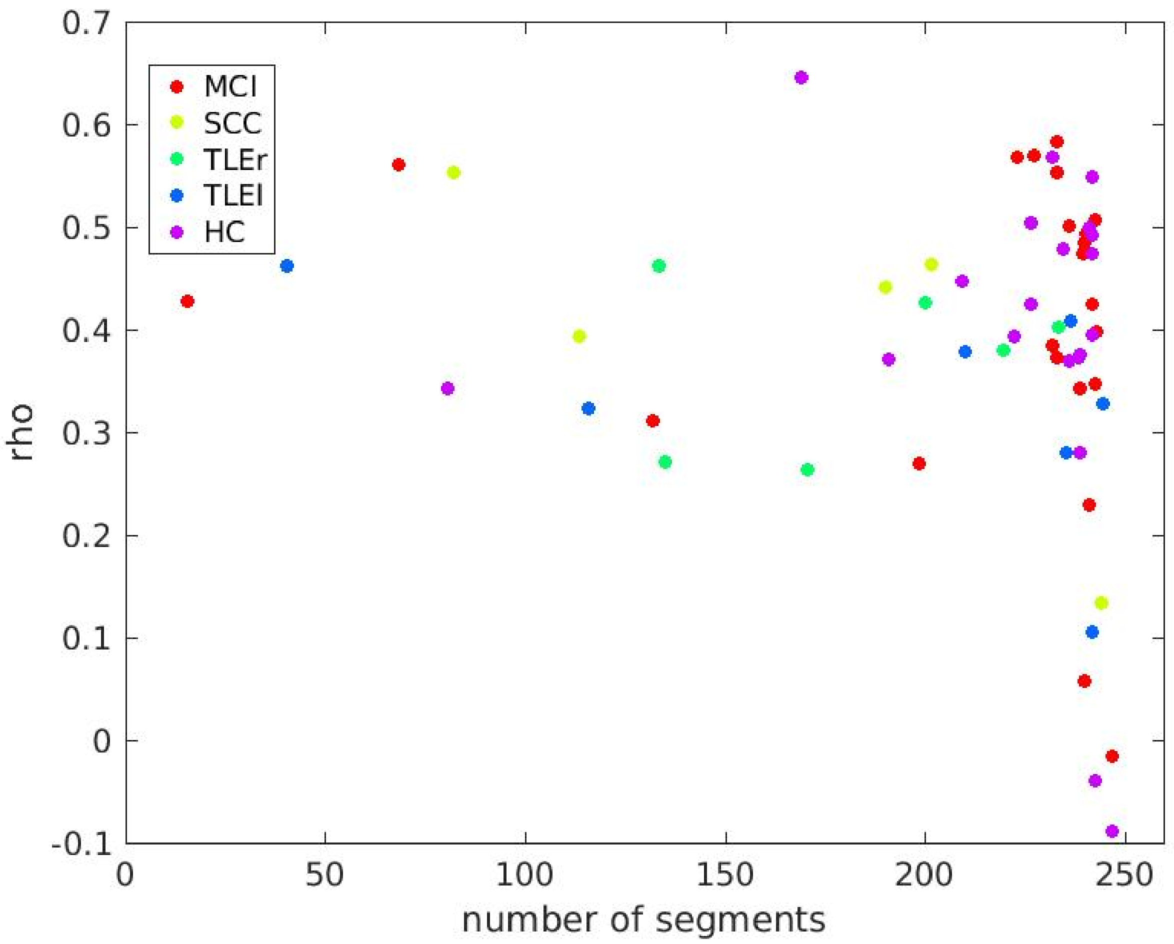

Figure S58. Scatter plot of test-retest reliabilities for directed transfer function vs. number of artefact-free segments of size 500ms. Calculation of test-retest reliabilities was based on segments without artefacts, only. The x-axis represents the average number of segments across two sessions included in the analysis. Colours indicate groups MCI= mild cognitive impairment, SCC=subjective cognitive complaints, TLEr= temporal lobe epilepsy with focus on the right hemisphere, TLEl= temporal lobe epilepsy with focus on the left hemisphere, HC=healthy controls.

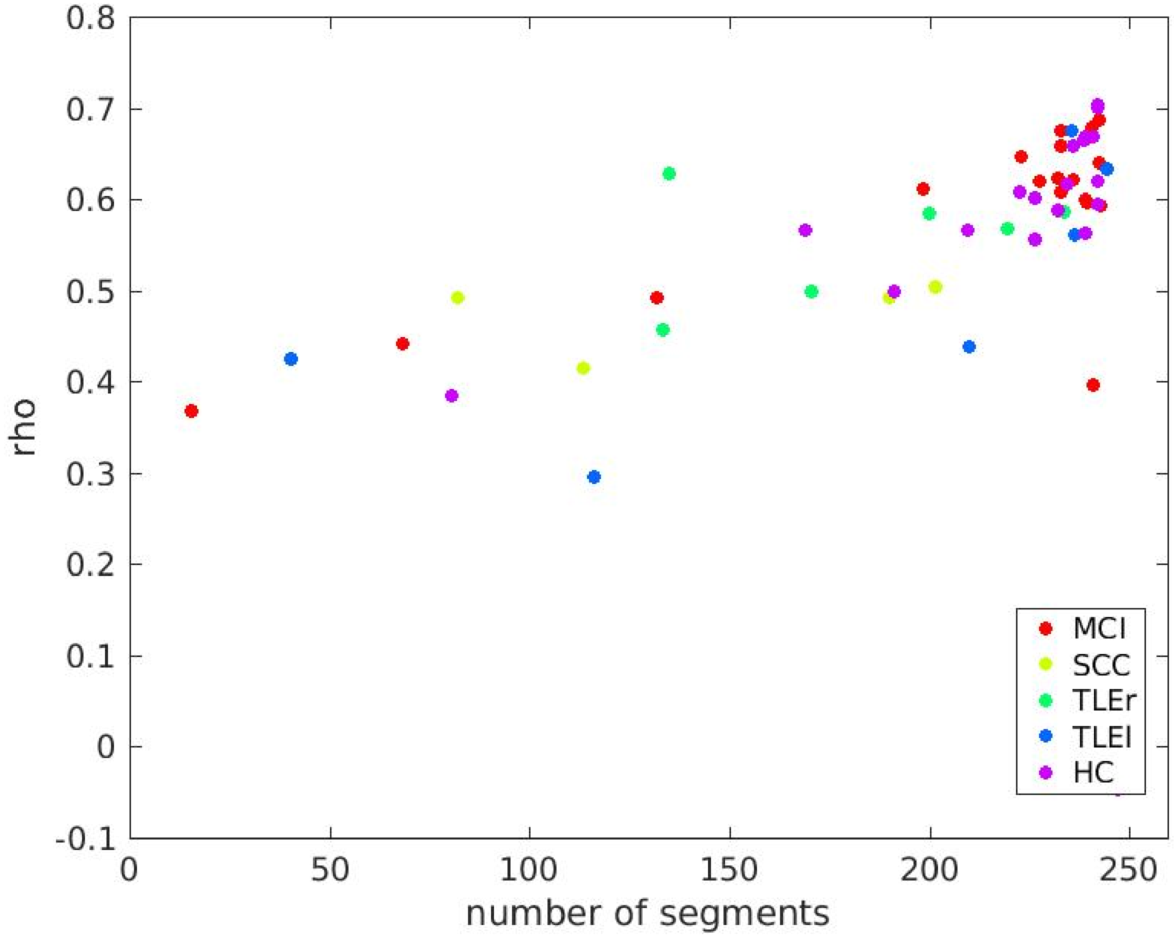

Figure S59. Scatter plot of test-retest reliabilities for direct directed transfer function vs. number of artefact-free segments of size 500ms. Calculation of test-retest reliabilities was based on segments without artefacts, only. The x-axis represents the average number of segments across two sessions included in the analysis. Colours indicate groups MCI= mild cognitive impairment, SCC=subjective cognitive complaints, TLEr= temporal lobe epilepsy with focus on the right hemisphere, TLEl= temporal lobe epilepsy with focus on the left hemisphere, HC=healthy controls.

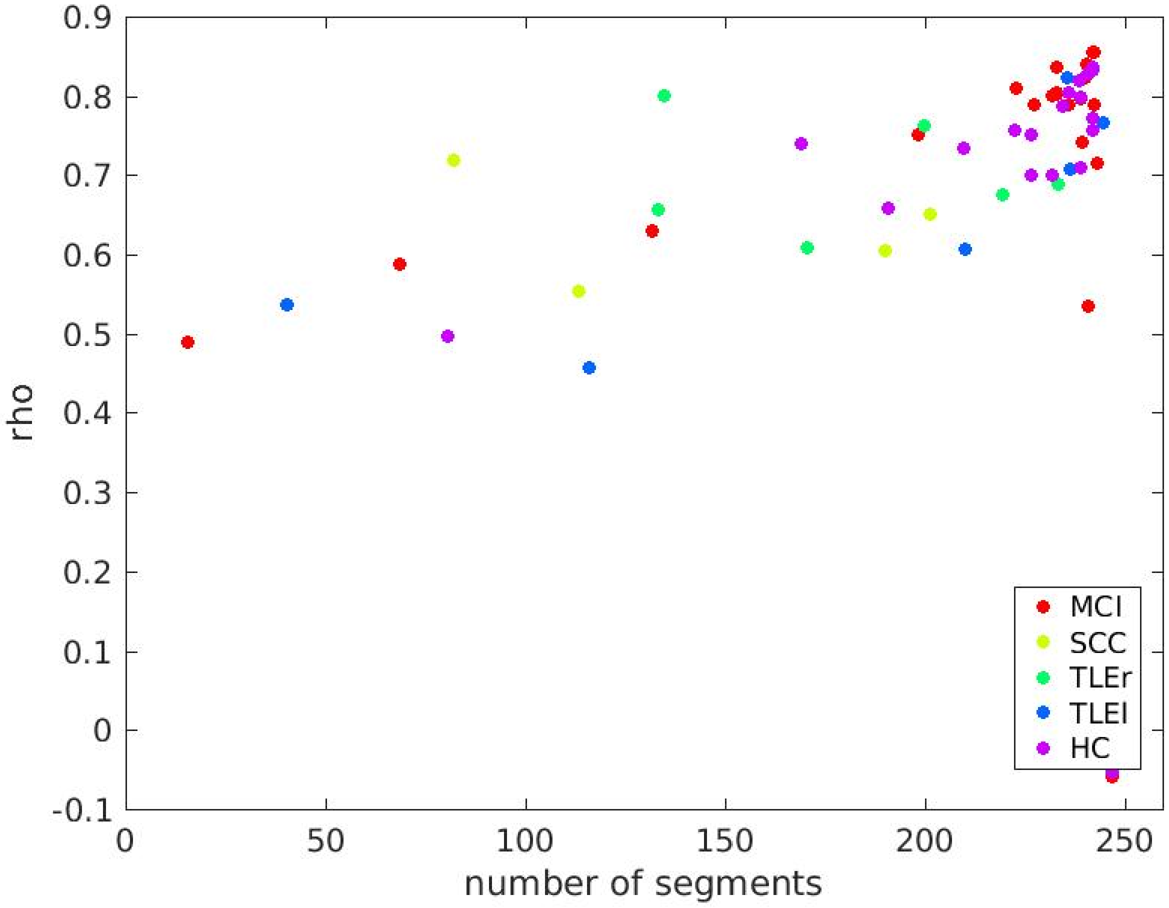

Figure S60. Scatter plot of test-retest reliabilities for full frequency directed transfer function vs. number of artefact-free segments of size 500ms. Calculation of test-retest reliabilities was based on segments without artefacts, only. The x-axis represents the average number of segments across two sessions included in the analysis. Colours indicate groups MCI= mild cognitive impairment, SCC=subjective cognitive complaints, TLEr= temporal lobe epilepsy with focus on the right hemisphere, TLEl= temporal lobe epilepsy with focus on the left hemisphere, HC=healthy controls.

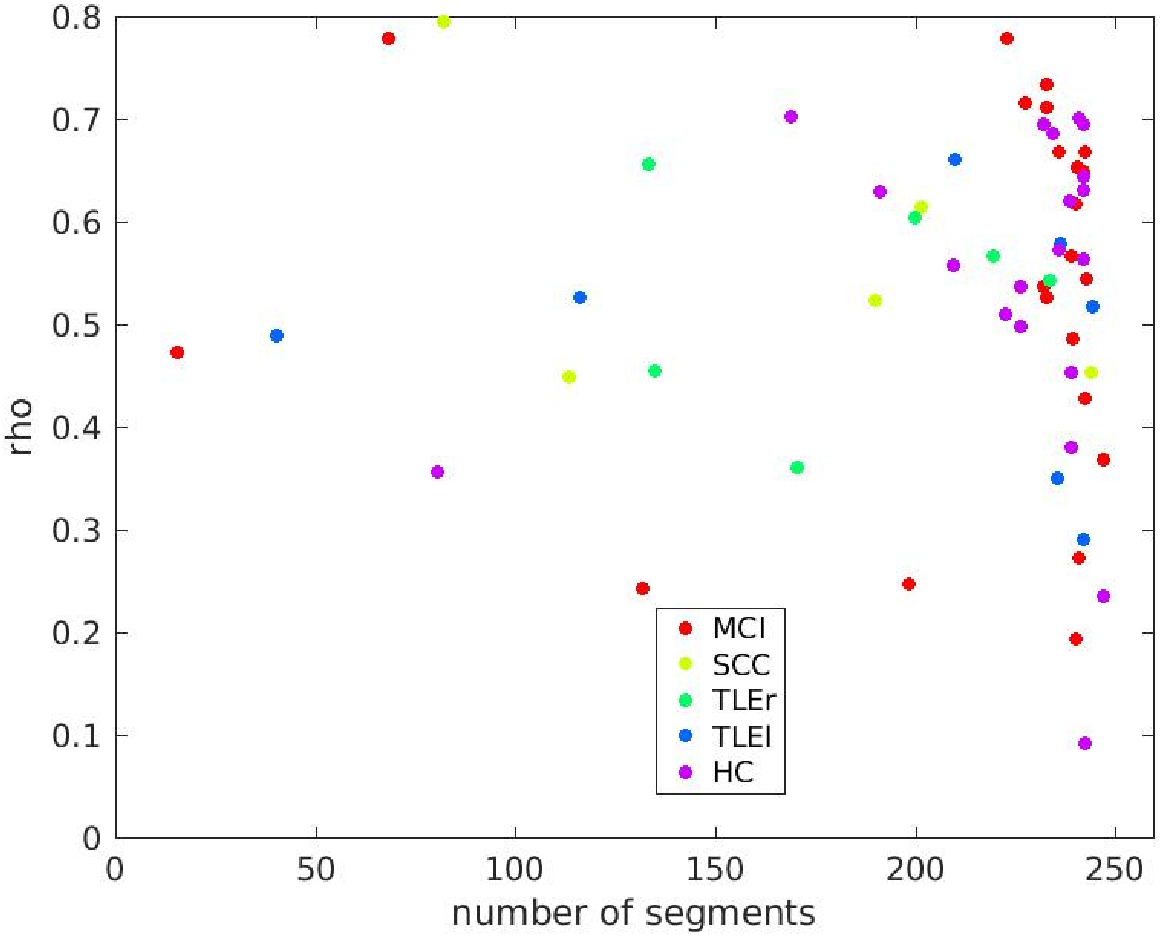

Figure S61. Scatter plot of test-retest reliabilities for Geweke's Granger causality vs. number of artefact-free segments of size 500ms. Calculation of test-retest reliabilities was based on segments without artefacts, only. The x-axis represents the average number of segments across two sessions included in the analysis. Colours indicate groups MCI= mild cognitive impairment, SCC=subjective cognitive complaints, TLEr= temporal lobe epilepsy with focus on the right hemisphere, TLEl= temporal lobe epilepsy with focus on the left hemisphere, HC=healthy controls.
